# Three genetic–environmental networks for human personality

**DOI:** 10.1038/s41380-019-0579-x

**Published:** 2019-11-21

**Authors:** Igor Zwir, Coral Del-Val, Javier Arnedo, Laura Pulkki-Råback, Bettina Konte, Sarah S. Yang, Rocio Romero-Zaliz, Mirka Hintsanen, Kevin M. Cloninger, Danilo Garcia, Dragan M. Svrakic, Nigel Lester, Sandor Rozsa, Alberto Mesa, Leo-Pekka Lyytikäinen, Ina Giegling, Mika Kähönen, Maribel Martinez, Ilkka Seppälä, Emma Raitoharju, Gabriel A. de Erausquin, Daniel Mamah, Olli Raitakari, Dan Rujescu, Teodor T. Postolache, C. Charles Gu, Joohon Sung, Terho Lehtimäki, Liisa Keltikangas-Järvinen, C. Robert Cloninger

**Affiliations:** 1 Department of Psychiatry, Washington University School of Medicine, St. Louis, MO USA; 2 Department of Computer Science, University of Granada, Granada, Spain; 3 Department of Psychology and Logopedics, University of Helsinki, Helsinki, Finland; 4 Department of Psychiatry, Martin-Luther-University Halle-Wittenberg, Halle, Germany; 5 Department of Epidemiology, and Institute of Health and Environment, School of Public Health, Seoul National University, Seoul, Korea; 6 Unit of Psychology, Faculty of Education, University of Oulu, Oulu, Finland; 7 Anthropedia Foundation, St. Louis, MO USA; 8 Department of Psychology, University of Gothenburg, Gothenburg, Sweden; 9 Blekinge Centre of Competence, Blekinge County Council, Karlskrona, Sweden; 10 Department of Clinical Chemistry, Fimlab Laboratories, and Finnish Cardiovascular Research Center—Tampere, Faculty of Medicine and Health Technology, Tampere University, Tampere, Finland; 11 University Clinic, Ludwig-Maximilian University, Munich, Germany; 12 Department of Clinical Physiology Tampere University Hospital, and Finnish Cardiovascular Research Center—Tampere, Faculty of Medicine and Health Technology, Tampere University, Tampere, Finland; 13 The Glenn Biggs Institute of Alzheimer’s and Neurodegenerative Disorders, Long School of Medicine, University of Texas Heath San Antonio, San Antonio, TX USA; 14 Department of Clinical Physiology and Nuclear Medicine, Turku University Hospital, Turku, Finland; 15 Centre for Population Health Research, Turku University Hospital, University of Turku Hospital, Turku, Finland; 16 Research Centre of Applied and Preventive Cardiovascular Medicine, University of Turku, Turku, Finland; 17 Department of Psychiatry, School of Medicine, University of Maryland, Baltimore, MD USA; 18 Rocky Mountain Mental Illness, Research, Education, and Clinical Center for Veteran Suicide Prevention, Denver, CO USA; 19 Division of Biostatistics, School of Medicine, Washington University, St. Louis, MO USA; 20 Department of Psychological and Brain Sciences, and School of Medicine, Department of Genetics, School of Arts and Sciences, Washington University, St. Louis, MO USA

**Keywords:** Genetics, Neuroscience, Psychology

## Abstract

Phylogenetic, developmental, and brain-imaging studies suggest that human personality is the integrated expression of three major systems of learning and memory that regulate (1) associative conditioning, (2) intentionality, and (3) self-awareness. We have uncovered largely disjoint sets of genes regulating these dissociable learning processes in different clusters of people with (1) unregulated temperament profiles (i.e., associatively conditioned habits and emotional reactivity), (2) organized character profiles (i.e., intentional self-control of emotional conflicts and goals), and (3) creative character profiles (i.e., self-aware appraisal of values and theories), respectively. However, little is known about how these temperament and character components of personality are jointly organized and develop in an integrated manner. In three large independent genome-wide association studies from Finland, Germany, and Korea, we used a data-driven machine learning method to uncover joint phenotypic networks of temperament and character and also the genetic networks with which they are associated. We found three clusters of similar numbers of people with distinct combinations of temperament and character profiles. Their associated genetic and environmental networks were largely disjoint, and differentially related to distinct forms of learning and memory. Of the 972 genes that mapped to the three phenotypic networks, 72% were unique to a single network. The findings in the Finnish discovery sample were blindly and independently replicated in samples of Germans and Koreans. We conclude that temperament and character are integrated within three disjoint networks that regulate healthy longevity and dissociable systems of learning and memory by nearly disjoint sets of genetic and environmental influences.

## Introduction

An individual’s unique pattern of behaviors, feelings, and thoughts is the expression of his or her personality, which is a strong predictor of the physical, mental, and social aspects of current and future health across the lifespan [1–3]. Personality is defined briefly as the way a person learns to adapt to experience, or, more specifically, as the dynamic organization within the individual of the psychobiological systems by which a person both shapes and adapts uniquely to an ever-changing internal and external environment [4].

Different genetic and neurobiological systems are involved in regulating distinct aspects of personality, which are traditionally described as temperament and character [5–8]. Temperament refers to innate biological predispositions that influence automatic emotional reactivity and habits; it is moderately stable throughout the lifespan, but can develop with aging and behavioral conditioning [1, 9, 10]. Put another way, temperament refer to the form or style of automatic behavior: *How* do you act and express yourself spontaneously [11, 12]? In contrast, character refers to mental self-government (i.e., what people make of themselves intentionally and creatively) [13], which develops in a saltatory manner throughout life [6, 14]. Learning to self-govern can be further partitioned by the basic questions that distinguish self-control and self-actualization. For self-control, we must answer the question “*What* do you intend to do?”, which involves the executive (intrapersonal) and legislative (interpersonal) functions of intentional self-control based on more-or-less logical calculation and analysis of personal goals, facts, beliefs, and social conventions. For self-actualization, we must answer a question about meaning in a transpersonal context: “*Why, Where, When* are you going to do it?”, which involves the judicial (transpersonal) functions of self-aware evaluation and intuitive appraisal of values and relationships as a theory or narrative with contextual insight into its meaning (why) at a particular place (where) and time (when) [4, 15]. Thus the architecture of human personality may correspond to the structure of human learning with its distinct systems for procedural, semantic, and self-aware learning and memory [16, 17]. Unfortunately, remarkably little is known about how the underlying genetic and environmental influences for these dissociable systems are integrated to express the complex phenotypes that we recognize as the self-organized profiles that describe personality and learning.

Human personality provides a highly instructive example of the challenges that must be faced in efforts to identify the molecular mechanisms involved in the causes and development of complex phenotypes [18–20]. In previous work, we found that the genetic variants associated with personality do not operate independently; rather they are organized as clusters of particular single-nucleotide polymorphisms (SNPs) that co-occur in subgroups of subjects, which we call SNP sets [18–20]. The SNP sets were each comprised of SNPs in many coding and noncoding genes that are distributed throughout the genome [21, 22], presumably due to the need for multiple genes to act in concert, not independently [20]. In general, geneticists must expect that each gene affects many traits and many genes affect each trait because evolutionary selection operates on whole organisms, not individual genes or traits [23]. Our specific findings confirmed consistent prior evidence from studies of twins and their families and from genome-wide association studies [24] that many genes act in concert with one another to influence human personality [25–28]. Thus the high heritability of personality expected from twin studies [25–28] was not missing, but was distributed into several disjoint components in which the subjects had distinct genotypic and phenotypic features [18–20].

Specifically, we found three clusters of people who were distinguished by heritable configurations of the temperament traits related to individual differences in procedural learning of habits and skills [19]. The three clusters were also characterized by individual differences in automatic behavioral activity and emotional expression, and corresponded closely to temperament clusters described as “easy”, “difficult”, and “slow to warm-up” [8, 11, 29] (Supplementary Information, see Tables S1, S2, and reference [8] for review). People in our “reliable” cluster resembled children with an “easy temperament” and adults who were conscientious extraverts because they were well controlled in activity and were warm and calm emotionally; put another way, they were high in Reward Dependence (i.e., sentimental, friendly, and approval seeking), low in Novelty Seeking (i.e., deliberate, thrifty, and orderly), low in Harm Avoidance (i.e., optimistic, confident, outgoing, and vigorous), and high in Persistence (i.e., determined). People in our “sensitive” temperament cluster resembled children with a “difficult temperament” and adults who are neurotic and unstable because they were under-controlled in activity and emotionally hypersensitive: in other words, they were high in Harm Avoidance (i.e., pessimistic, fearful, shy, and fatigable), high in Novelty Seeking (i.e., impulsive and extravagant), and high in Reward Dependence (i.e., sentimental and friendly), so they frequently had approach-avoidance conflicts, rejection sensitivity, and disorganized attachments. People in our “antisocial” temperament cluster resembled children with a “slow to warm-up” temperament and adults who are socially detached, careless, and impulsive: that is, they were low in Reward Dependence (i.e., cold, detached, and independent), low in Persistence (i.e., easily discouraged), and high in Novelty Seeking (i.e., extravagant, rule-breaking but not inquisitive), which is frequently associated with maladaptive antisocial conduct. Furthermore, the genes associated with each of these three temperament profiles were largely unique to that profile.

Likewise, we identified five heritable clusters of people with distinct profiles of character traits involving various combinations of high versus low scores on Self-directedness, Cooperativeness, and Self-transcendence [18]. Three of these profiles were usually associated with physical, mental, and social well-being, and were described as resourceful (i.e., high in Self-directedness only), organized (i.e., high in both Self-directedness and Cooperativeness, but not Self-transcendence), or creative (i.e., high in all three character traits). The other two were usually associated with poor physical, mental, and social functioning, and were described as “dependent” (high in Cooperativeness but low in Self-directedness and Self-transcendence) or “apathetic” (low in all three character traits). As with temperament, the genes associated with each of the five character profiles were largely different.

We found that personality depends on sets of genes that regulate and coordinate the dynamic functions required for people to learn to adapt to changing circumstances, including molecular processes for neurodevelopment, neuroplasticity, neurogenesis, neurotransmission, stress reactivity, energy metabolism, neuroprotection, resilience, and healthy longevity [18, 19]. The genes we found to be associated with personality were nearly always expressed in the brain, but these brain functions depended on interactions with variability in genes regulating pathways that are particularly important in brain but involve general housekeeping functions that occur in most or all cell types, such as the regulation of energy metabolism, circadian rhythmicity, and cellular repair [30, 31].

Furthermore, we found that the genes that encode variability in human temperament are enriched in highly conserved molecular pathways, the Ras-MEK-ERK and PI3K-AKT-mTOR pathways, which are activated in experimental animals by stress reactivity and associative conditioning (i.e., psychobiological system 1 for classical and operant conditioning) [32–35]. For example, the responses of these pathways to patterns of reward and punishment regulate neuroplasticity in the striatum, thereby modulating the integration of frontocortical and mesolimbic signaling during associative conditioning in humans and other amniotes (i.e., reptiles, birds, and mammals) (see Supplementary Information, Vignette 1) [36–38].

In contrast, the genes we found encoding human character are associated with two brain systems for higher cognitive processes involving intentional self-control or self-awareness [16, 17, 39]. Specifically, a second network (psychobiological system 2) involves specialized bipolar neurons in the anterior insular cortex, frontal operculum, and anterior cingulate cortex that are present in great apes and humans, but not in other primates [40, 41]. These Von Economo neurons are functionally connected to temporal and parietal neocortical regions in brain circuits that support saliency detection, resolution of emotional conflicts, and social cooperation for mutual benefit in great apes and humans [40–43]. This system also supports intentional self-control of voluntary behavior and purposeful use of symbols with further development of the inferior parietal cortex as a convergence area for touch, hearing, and vision in humans (see Supplementary Information, Vignette 2) [40, 44]. Another network (psychobiological system 3) involves regions of late-myelinating neocortex in frontal, parietal, and temporal regions found only in humans, and is associated with the emergence of human capacities for self-awareness, insight (i.e., immediate, accurate, and deep intuitive understanding), creative imagination, altruism, and autobiographical memory (see Supplementary Information, Vignette 3) [40, 44–46]. These three brain networks normally interact in a coordinated manner [47–49], but they are dissociable developmentally [17, 46, 50] and functionally [17, 48, 49, 51–54].

Our previous findings also suggest the hypothesis that different molecular processes may regulate associative learning, intentionality, and self-awareness. Specifically, we uncovered largely disjoint sets of genes regulating these three distinct learning processes in different clusters of people with unhealthy temperament profiles (network 1 with associative conditioning), organized character profiles (network 2 with intentionality), and creative character profiles (network 3 with self-awareness), respectively [18, 19]. However, little is known about the molecular processes by which temperament and character are organized into integrated networks, how such integrated networks are associated with specific genetic and environmental influences, or how crucial such integration is for health and well-being.

Our computational approach to genome-wide association study (GWAS) is an extension of the efforts of many geneticists to analyze GWAS in terms of sets of multiple markers that are significantly and reproducibly related to complex phenotypes, rather than single markers that neglect the complex interactions among multiple variables [18, 19, 21], or polygenic risk scores that depend on multiple markers neither significantly nor consistently related to the phenotype [55–58] (See Supplementary Information for a review). There are a wide range of possible models that may account for the missing heritability in GWAS by consideration of interactions among multiple genetic, cultural, and environmental variables [59–62]. At one extreme, polygenic risk scores are computed as the sum of the effects of many genes acting independently in a linear regression model [20]. In contrast, the omnigenic model assumes a more structured architecture in which there are core genes with large effects on phenotype [63, 64], as well as peripheral genes distributed throughout the genome with weak individual effects [65].

Rather than assume any particular model, in this study we have used unsupervised learning to uncover the genetic, environmental, and phenotypic architecture of complex personality clusters and then replicate our findings in independent samples from different cultures. As in our prior work [18, 19], we use data-driven methods to uncover the joint organization of deep networks of temperament and character profiles, as well as the genetic and environmental architecture of their joint relationship. We hypothesize that the genetic networks associated with the joint relations of temperament and character networks may reflect the three different systems of learning and memory that emerged separately in the long phylogeny of human beings as incrementally more conscious and flexible ways of adapting to changing external environmental conditions and internal aspirations [40]. We test this hypothesis in a data-driven analysis so that our hypotheses do not bias or restrict our results.

## Subjects and methods

### Description of the samples

Our discovery sample was the Young Finns Study, an epidemiological study of 2149 healthy Finnish subjects. All subjects had thorough standardized genotypic, environmental, and phenotypic assessments, including administration of the Temperament and Character Inventory (TCI) [10, 66]. We replicated the results in two independent samples of 902 healthy adults from Germany [67] and 1052 from Korea [68, 69] in which comparable genotypic and phenotypic features were available [18, 19].

### Personality assessment

All subjects completed the TCI to assess seven heritable dimensions of personality [13] (see Supplementary Information, Table S1).

### Personality health indices

People at risk of poor adaptive functioning were identified as the bottom decile of the sum of TCI Self-directedness and Cooperativeness [3]. In contrast, people with strong adaptive functioning were identified as the top decile of the product of all three TCI character traits, as in prior work [3, 70]. We confirmed the validity of these indicators of health in our discovery sample with independent measures of positive affect balance, perceived social support, physical behaviors (exercise, smoking, and diet), and objective laboratory findings for ideal health recommended by the American Heart Association [71–73] (Supplementary Tables S3A, B). Our indices provided a consistent measure of health status in all three samples.

### Environmental assessments

The environmental variables measured for the Finns included reports by the main caretaker (usually the mother) of parental tolerance (i.e., acceptance of the child), emotional warmth (i.e., nurturance of the child), strict disciplinary style, as well as the parent’s education, income, and the family’s urban/rural residency during childhood in 1980 and 1983 [18, 19], and stressful life events and urban versus rural residency during adulthood in 2001 [18, 19]. Comparable environmental data about the German and Korean subjects were not available.

### Computational procedures

Our machine-learning approach [74, 75] uses the nonnegative matrix factorization (NMF) method, which identifies multidimensional patterns within different types of data. Our deep unsupervised NMF process uncovers naturally occurring associations between patterns across different types of data. This algorithm is described in Supplementary Fig. S1 and elsewhere [18, 19].

Our web server application for phenotype–genotype many-to-many relations analysis (PGMRA) in GWAS is published [76] and online at http://phop.ugr.es/fenogeno.

### Replications

PGMRA was used to uncover the complex genotypic–phenotypic associations in two replication samples (Germans and Koreans) independent of information about the discovery sample. The process used in the discovery sample was blindly and independently repeated in each replication sample without assuming homogeneity within or across samples [20]. We accounted for ethnicity in each sample by using the first three principal components for ancestral stratification of SNP genotypes, as in prior work [18, 19]. Then matching of genotypic–phenotypic associations across samples was identified using parsimonious models that balance accuracy with model complexity, thereby avoiding overfitting [77]. Models were learned independently in diverse samples to provide a stringent test of reproducibility despite complexity that might result from possible genetic, ethnic, cultural, and environmental heterogeneity [18–20].

## Results

### Identifying phenotypic networks of temperament and character

We identified 44 fine-grained sets of subjects with distinct configurations of TCI character subscales regardless of their temperament (i.e., character sets or biclusters) using PGMRA, as we have previously reported [18]. The character sets varied in personality features (TCI subscales), numbers of subjects, and health status (well- or ill-being, Supplementary Table S4). The character sets were grouped into five deep clusters (i.e., character profiles) by recurrently applying PGMRA for convolutive NMF, minimizing the Cophenetic correlation coefficient [18]. These five clusters were labeled as Resourceful (i.e., self-directed only), Organized (i.e., self-directed and cooperative), Creative (i.e. self-directed, cooperative, and self-transcendent), Dependent (i.e., cooperative only), or Apathetic (low in all three character scales) using traditional labels [18] (Supplementary Table S1).

Likewise we identified 55 fine-grained sets of subjects with distinct configurations of TCI temperament subscales (i.e., temperament sets) using PGMRA, as we have previously reported [19] (Supplementary Table S4). The temperament sets were grouped in three deep clusters (i.e., temperament profiles) by recurrently applying PGMRA for convolutive NMF, minimizing the Cophenetic correlation coefficient [19]. These distinct temperament profiles were labeled as Reliable (i.e., low in Novelty Seeking and Harm Avoidance, high in Reward Dependence and Persistence), Sensitive (high in Harm Avoidance, Novelty Seeking, and Reward Dependence), or Antisocial (High in Novelty Seeking, and low in Reward Dependence and Persistence) using traditional labels [19].

**Fig. 1:**
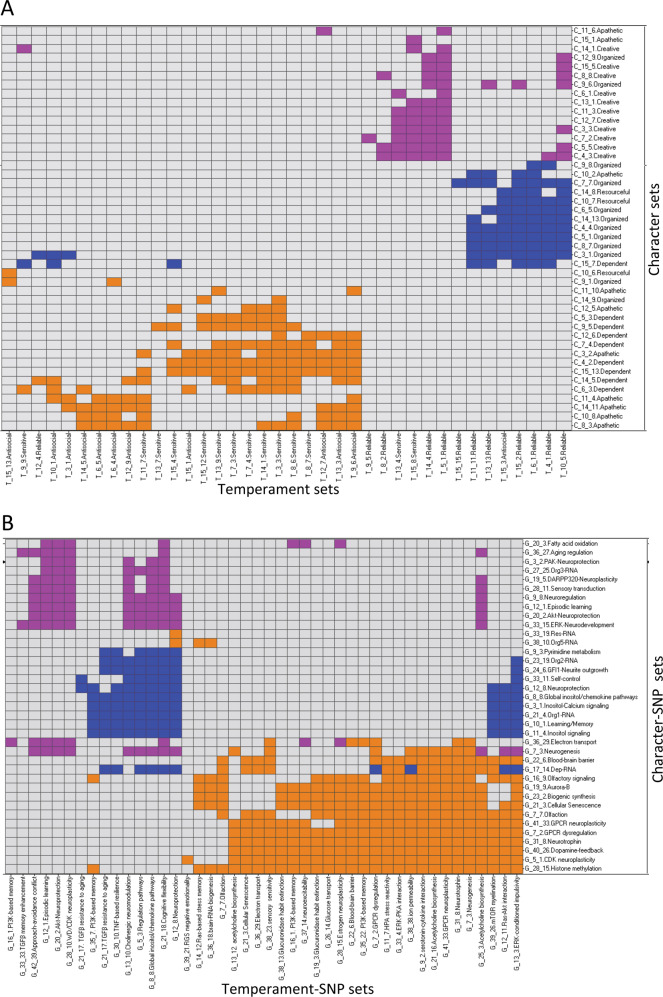
The phenotypic architecture of personality. **a** Relationships among Temperament and Character Sets are naturally partitioned into three subnetworks using NMF (bidirectional-clustering) techniques: Creative-Reliable (violet), Organized-Reliable (blue), and Emotional-Unreliable (orange). **b** Relationships among SNP sets associated with Temperament and Character Sets composing the three networks shown in **a**: self-awareness (violet), self-control (blue), and emotional reactivity (orange)

Next we tested for associations among the temperament and character sets sharing the same individuals. We found 265 deep phenotypic associations among 39 temperament sets and 46 character sets by recurrently applying PGMRA for convolutive learning. These associations were statistically significant by Fisher’s exact test (5E−14 < *p* < 1E−05, Supplementary Table S4 and Fig. S1) and by a permutation test (empirical *p* < 5E−04). To assess the phenotypic architecture of these relationships, we identified subgroups of subjects with distinct joint temperament–character features using PGMRA. These associations were organized as three deep phenotypic networks that were nearly disjoint (i.e., shared few subjects between different temperament–character associations), as shown in Fig. 1a.

**Fig. 2:**
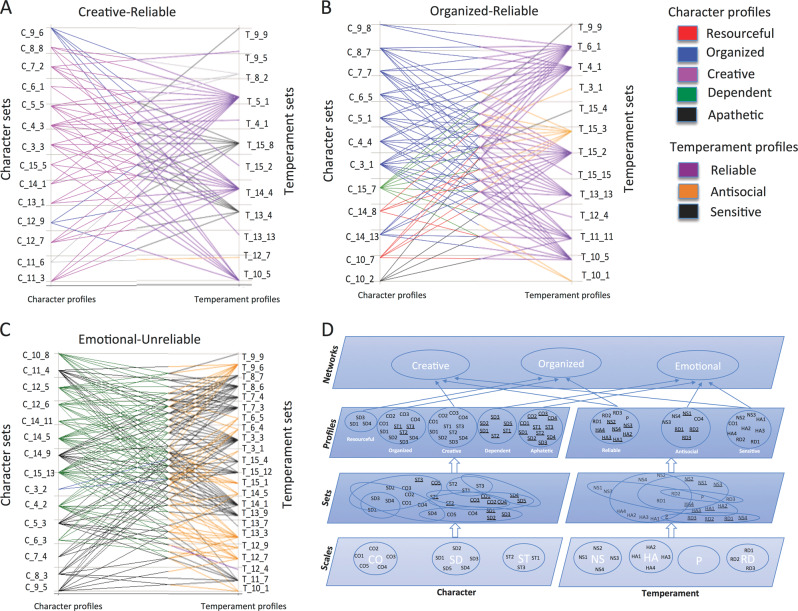
Relationships among Temperament and Character Sets composing the three networks shown in Fig. 1. **a** The Creative-Reliable subnetwork (light violet, violet) contains primarily Character Sets with creative subjects, who also display a reliable temperament. **b** The Organized-Reliable subnetwork (blue, violet) primarily contains Character Sets with organized subjects, who also display reliable temperament. **c** The Emotional-Unreliable subnetwork (green, black, and orange) contains Character Sets with dependent and apathetic subjects, who also display sensitive and antisocial temperaments. **d** The hierarchical organization of personality: temperament and character scales and subscales, sets of subscales (see Table S3), profiles, and networks

Despite the three phenotypic networks being nearly disjoint from one another, the associations among the temperament and character sets within each network were highly complex. In other words, within each network the same character set was associated with multiple temperament sets, and different character sets could be associated with the same temperament set (Fig. 2). The networks are represented at different levels of granularity in Fig. 2d. In order to make terminology clear, the deep organization of the personality networks is displayed in Fig. 2d as a hierarchy of deep descriptive complexity ascending from (i) separate scales and subscales of temperament and of character, (ii) sets of multiple subscales of temperament and of character, (iii) profiles of temperament sets and of character sets, and (iv) joint networks of temperament and character profiles.

Specifically, the healthiest phenotypic network is primarily comprised of subjects with a Creative character profile associated with a Reliable temperament profile (66% of 56 character–temperament set combinations) (see Figs. 1a and 2a, Supplementary Fig. S2A, D). Therefore, we called it the Creative-Reliable network, but it also includes some subjects with Organized characters (2 of 14 character sets in network) and/or Sensitive temperaments (3 of 12 temperament sets in network) as a result of the complex relations within the network (see Fig. 2a). The second phenotypic network was largely comprised of individuals with an Organized character profile associated with a Reliable temperament profile (72% of 72 character–temperament set combinations) (see Figs. 1a and 2b, Supplementary Fig. S2B, D). It was named the Organized-Reliable network but included some individuals with a Resourceful character (2 of 12 character sets in network) and/or antisocial temperament (3 of 13 temperament sets in network) (see Fig. 2b). The least healthy phenotypic network was named the Emotional-Unreliable network because it was comprised of emotionally reactive, injudicious, and maladapted individuals with Dependent characters (8 of 15 character sets in network) or Apathetic characters (6 of 15 characters sets in network) associated with Sensitive temperaments (12 of 23 temperament sets in network) or Antisocial temperaments (11 of 23 temperament sets in network) (see Figs. 1a and 2c, Supplementary Fig. S2C, D). Both the dependent and apathetic character profiles were usually combined with the sensitive temperament: 82% of 72 combinations for the dependent profile, and 58% of 45 combinations for the apathetic character profile (see Fig. 2c).

The three networks were similar in terms of numbers of constituent sets and subjects: there were 674, 801, and 603 subjects for networks 1 (emotional-unreliable), 2 (organized-reliable), and 3 (creative-reliable), respectively (Supplementary Table S5). In total 4.6% of subjects were not assigned to a network because they did not reach the threshold for significant association with a particular network. A prototypical vignette for each network is presented in Supplementary Information.

**Fig. 3:**
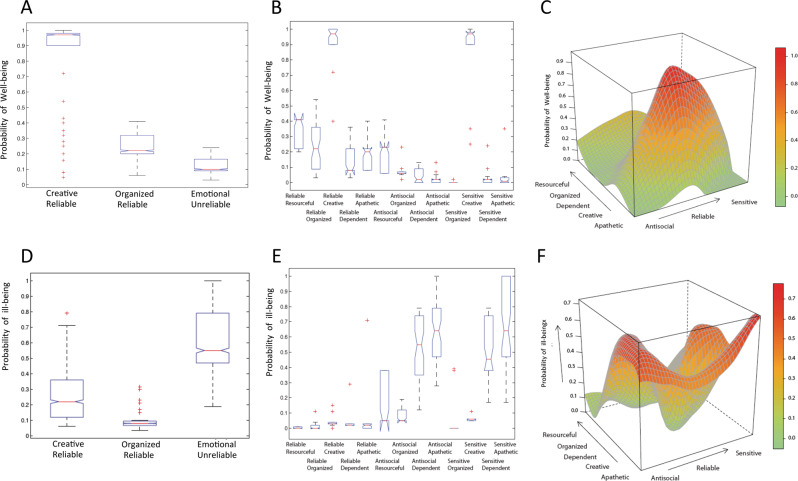
Evaluation of the probability of health measured for Temperament and Character associations in the three phenotypic subnetworks using ANOVA statistics (*p* value < 1E−20), and *T*-test among the three subnetworks. The three networks, Creative-Reliable, Organized-Reliable, and Emotional-Unreliable, significantly differ from each other (*p* value < 1E−04) in their probability of well-being (**a**) and ill-being (**d**). **b**, **e** Evaluation of probability of health in Temperament and Character Sets and their relationships with SNP Sets using ANOVA statistics. **b** Well-being and **e** Ill-being evaluated for Temperament and Character Sets with respect to their profiles. **c**, **f** Surfaces representing the health function of the uncovered relationships between Temperament and Character Sets. The probability of health (*z*-axis; red high; green: low) was calculated based on the distribution of the status of subjects within each relationship, and the surface was plotted interpolating the relation domains. The order adopted for plotting relationships are calculated based on clustering shared subjects in Character (*x*-axis) and in Temperament (*y*-axis) Sets using Hypergeometric statistics (see “Method” section). (Close-located sets in an edge share more subjects than those located far away.) **c** Well-being surface. **f** Ill-being surface

The health status of subjects also strongly distinguished the networks. The Creative-Reliable network had the highest levels of well-being, but also had a slightly higher risk of ill-being than the Organized-Reliable network (Fig. 3a–d, Supplementary Fig. S3). As expected, the Emotional-Unreliable network had the highest level of ill-being and the lowest level of well-being (*p* < 1.06E−23, ANOVA, Fig. 3a, d–f). Overall, the three networks differed significantly from each other in the well-being and ill-being indices (ANOVA, *p* < 2.29E−26).

We observed that the effect size on the well-being of subjects in different temperament–character configurations did not depend on the strength of the temperament–character association or number of subjects in their intersection. We also estimated the effect size of differences between the sets in each of the three networks by comparing their means and standard deviations for well-being and ill-being. The differences in the means of well-being for the sets in the creative-reliable (0.87 ± 0.21), organized-reliable (0.23 ± 0.012), and emotional-unreliable (0.022 ± 0.030) networks were highly significant (One-Way ANOVA, *F* = 7388.04, df = 2, *p* < 0.0001). The differences in the means for ill-being for the sets in the creative-reliable (0.63 ± 0.14), organized-reliable (0.018 ± 0.039), and emotional-unreliable (0.62 ± 0.21) networks were also highly significant (one-way ANOVA, *F* = 2651.63, df = 2, *p* < 0.0001). For well-being, the differences between the means of the sets in each pair of networks were two to four times their standard deviations, which was significant (*p* < 0.01) for each pair using Tukey’s range HSD test. For ill-being, the differences between the means of the sets that made up the creative-reliable and emotional-unreliable networks was four standard deviations, larger (15 standard deviations) for the differences between the means of organized-reliable and emotional-unreliable networks, and smaller (0.33 standard deviation) for the differences between the means of creative-reliable and organized-reliable networks. Although the differences in mean levels of ill-being between networks varied in size, they were all significant (*p* < 0.01) by Turkey’s range HSD test. In brief, there were large differences in the size of the differences in means of each pair of networks for both well-being and ill-being, except that the greater risk of ill-being in the creative-reliable network compared with the organized-reliable network was small, as illustrated in Fig. 3.

### Identifying genetic networks associated with the phenotypic networks

We identified 66 SNP sets associated with at least one joint temperament–character association (Fig. 1b and Supplementary Tables S6 and S7). Fourteen SNP sets were previously deeply related to both character and temperament sets [18, 19]. Another 24 SNP sets were previously directly associated with character sets and indirectly with other temperament sets. Another 28 SNP sets were directly associated with temperament sets and indirectly with other character sets. Thus there were 66 SNP sets associated with at least one joint temperament–character relationship.

**Fig. 4:**
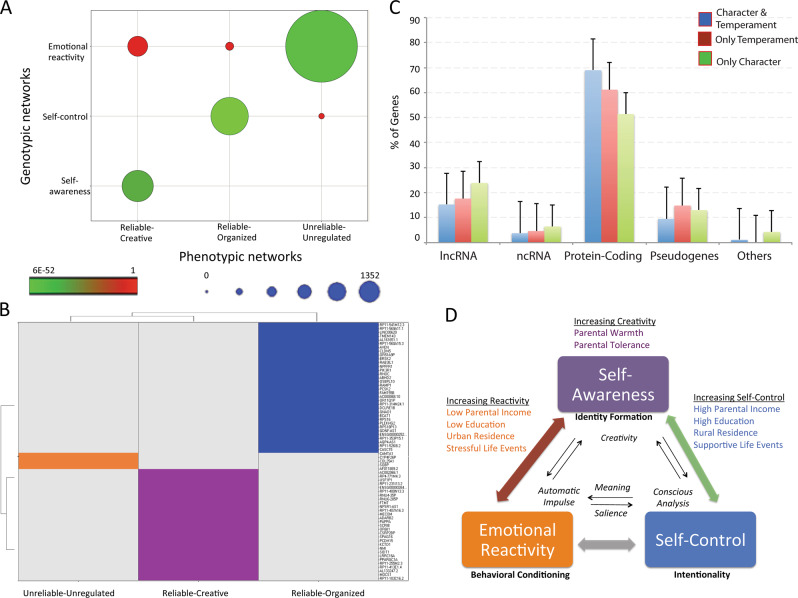
**a** Correlation between the phenotypic (Fig. 1a) and the genotypic (Fig. 1b) networks (*p* < 6E−52, Hypergeometric statistics). Color codes indicate low (red) to high (green) statistical significance. The size of the circles indicates the number of coincident phenotypic–genotypic relationships. **b** Relationships among key genes associated with Temperament and Character Sets that discriminate the three networks shown in Fig. 1a, b: self-awareness (violet), self-control (blue), and emotional reactivity (orange). (See AND/OR relationships in Fig. S5.) **c** Types of genetic variants mapped by SNP sets associated with character: specific molecular consequences [genes related only to character sets (green) were less often protein coding and more often RNA genes than those also associated with temperament sets (red color), or genes related to both character and temperament exhibit higher proportion of protein-coding genes. See subtypes in Fig. S4B]. **d** Relationships among environmental sets associated with Genotypic subnetworks (Fig. 1). Environmental sets can belong to one or more networks

Nearly disjoint clusters of these 66 SNP sets distinguished the three phenotypic networks, as shown in Fig. 1b. In other words, the SNP sets associated with joint character–temperament relations were organized as three genotypic networks (Figs. 1b and 4a). Based on their distinguishing molecular processes (Supplementary Tables S6 and S7), the genotypic networks were labeled “emotional reactivity”, “self-control”, and “self-awareness” to describe the basic functional process of each briefly.

Each of the phenotypic networks was strongly associated with only one of the genotypic networks, as shown in Fig. 4a: the Creative-Reliable network with the genotypic network for self-awareness, the Organized-Reliable network with the genotypic network for intentional self-control, and the Emotional-Unreliable network with the genotypic network for emotional reactivity and associative conditioning. There was little genotypic overlap among the three phenotypic networks: that is, few of the SNP sets associated with character (0–3%) or temperament (4.6–10.6%) were shared by any of the possible combinations of the three phenotypic networks (Supplementary Table S8).

In addition to the SNP sets associated with the integrated phenotypic network, we identified 759 genes that were associated with one or more of the three phenotypic sets in a robust way (i.e., the gene was present in multiple network-associated SNP sets). Another 213 genes were associated with the phenotypic networks, but were recognized in only one SNP set. All 972 genes are listed in Supplementary Tables S9 and S10 along with the gene’s name, type, known functions, and associated phenotypic network. The types of genes and their chromosomal locations are shown in Fig. 4b, c, Supplementary Figs. S4 and S5, Tables S9 and S10.

Among the 759 genes robustly associated with one or more of the three phenotypic networks, we found that 67.2% were unique to a single-phenotypic network: 265 for the Creative-Reliable network, 211 for the Organized-Reliable network, and 34 for the Emotional-Unreliable network. Among all 972 genes associated with one or more of the three phenotypic networks, we found that 72.4% were unique to a single-phenotypic network: 327 for the Creative-Reliable network, 273 for the Organized-Reliable network, and 106 for the Emotional-Unreliable network. Examples of the various types of genes that are unique to each of the phenotypic networks are displayed with their names in Fig. 4b. The full list of genes and their functions is available in Supplementary Fig. S5, Supplementary Tables S9 and S10.

### Identifying environments that distinguished the phenotypic networks

We also tested sets of environmental variables for their ability to distinguish the three phenotypic networks. The Environmental sets are clusters of subjects with particular measured environmental experiences. There were 22 Environmental sets directly and deeply associated with the temperament and/or character components of the three networks (Table 1 and Supplementary Table S11). Another 24 Environmental sets were indirectly related with the networks as a result of gene–environment interactions (i.e., with network-associated SNP sets as mediators) (Table 1). Eight environmental sets were both directly and indirectly related to the three networks (Table 1, Supplementary Fig. S6 and Table S11).

**Table 1:** Environmental sets related to the three phenotypic networks. Environmental sets are described by their features, and direct and indirect associations with the Temperament and Character phenotypes within each network. *see Supplementary Table S11 for additional description of the variables

Direct associations	Description				Temperament associations			Character associations		
					Reliable-creative	Reliable-organized	Emotional-unregulated	Reliable-creative	Reliable-organized	Emotional-unregulated
Environment sets										
E_10_1	High parental education 1983	High parental education 1980						Yes		
E_14_7	High parental education 1983	High parental education 1980						Yes		
E_5_2	Rural residency 1980	Rural residency 1983						Yes		
E_6_2	Rural residency 1980	Rural residency 1983						Yes		
E_6_4	High parental income 1980	High parental income 1983						Yes		
E_7_5	Rural residency 2101				Yes				Yes	Yes
E_14_14	High income 1980								Yes	Yes
E_11_4	Rural residency 2101								Yes	
E_13_13	Rural residency 2101								Yes	
E_8_1	Rural residency 1980	Rural residency 1983							Yes	
E_15_15	Stressful life events								Yes	
E_13_6	Rural residency 1980	High parental income 1983	High parental income 80			Yes			Yes	
E_15_14	Rural residency 1980	High parental income 1983	High income 80			Yes			Yes	
E_8_6	Rural residency 2101					Yes			Yes	
E_10_2	Stressful life events					Yes				
E_11_7	Stressful life events					Yes				
E_9_5	Stressful life events					Yes				
E_9_7	High parental income 1980	High parental income 1983				Yes				
E_13_4	High Parental Income 1980									Yes
E_14_10	Urban residency 1980	High parental income 1983								Yes
E_3_2	Stressful life events	Low parental income 1980	Rural residency 2101							Yes
E_8_8	Parental tolerance 1983	Parental tolerance 1980	Parental emotional warmth 83							Yes
Indirect associations										
E_13_9	Low parental income 1980	Low parental income 1983			Yes			Yes		Yes
E_14_12	Low parental income 1980	Low parental income 1983						Yes		Yes
E_8_5	Low parental income 1980	Low parental income 1983			Yes			Yes		Yes
E_10_2	Stressful life events				Yes			Yes		
E_13_10	High parental education 1983	High parental education 1980			Yes			Yes		
E_13_11	Stressful life events							Yes		
E_14_1	Parental tolerance 1980				Yes			Yes		
E_14_5	Stressful life events				Yes			Yes		
E_15_15	Stressful life events				Yes			Yes		
E_15_5	High parental emotional warmth 1983	High parental emotional warmth 1980			Yes			Yes		
E_6_1	Rural residency 2101				Yes			Yes		
E_6_2	Rural residency 1980	Rural residency 1983			Yes			Yes		
E_10_6	Parental tolerance 1980	Parental tolerance 1983	High parental emotional warmth 1983		Yes					
E_11_7	Stressful life events				Yes					
E_15_4	Low parental income 1980				Yes					
E_9_5	Stressful life events				Yes					
E_15_2	High parental education 1983	High parental education 1980			Yes	Yes		Yes	Yes	
E_7_2	Stressful life events					Yes		Yes		Yes
E_14_3	High parental income 1983					Yes			Yes	
E_14_2	Rural residency 2101								Yes	
E_15_8	Rural residency 2101								Yes	
E_4_3	Urban residency 1980	Urban residency 1983							Yes	
E_5_1	Urban residency 1980	Urban residency 1983							Yes	
E_7_5	Rural residency 2101								Yes	
E_11_11	Low parental income 1980	Low parental income 1983								Yes
E_14_10	Urban residency 1980	High parental income 1983								Yes
E_3_2	Stressful Life Events	Low Parental Income 1980	Rural Residency 2101							Yes
E_5_4	Urban residency 2101	Low parental education 1980	Low parental education 1983	Low parental income 1980						Yes
E_5_5	Rural residency 2101									Yes
E_7_4	Low parental income 1980	Low parental income 1983								Yes
E_8_7	Stressful life events									Yes
E_9_6	Low parental income 1980	Low parental income 1983								Yes

Environmental sets were associated with 18.5% of the 265 temperament–character relationships. They were directly associated with 7.6% of 265 temperament–character relationships on average in any network, but most direct relations occurred with only the Organized-reliable network (26%, Supplementary Table S12). Environmental sets were, on average, indirectly associated with 10.9% of all temperament–character relationships, and there were substantial numbers of associations with each phenotypic network: 5.3% with the creative-reliable network, 11.0% with the organized-reliable network, and 13.3% with the emotional-unreliable network (Supplementary Table S12).

We found the environmental sets distinguished the three phenotypic sets in a nearly disjoint fashion, as shown in Fig. 4d and Supplementary Fig. S6. The features of the environmental sets are detailed in Table 1, Fig. 4d, and Supplementary Table S11.

### Genotypic–phenotypic relationships influence health

The probabilities of well-being and ill-being were evaluated in each of the phenotypic networks using phenotypic information alone (Fig. 3, Supplementary Fig. S3) and together with genotypic information (Supplementary Fig. S7). Combining genotypic and phenotypic information provided more information than the phenotypes alone for both well-being (Fig. 3b, c vs Supplementary Figs. S3A, S7A) and ill-being (Fig. 3e, f vs. Supplementary Figs. S3B and S7B). When health indices were based on the joint genotypic–phenotypic relationship instead of only the phenotype, all possible combinations of temperament and character profiles were strongly distinguished by their probabilities of well-being (ANOVA, *p* value < 4.75E−31) and ill-being (ANOVA, *p* value < 1.35E−49). Therefore health status provided a general characteristic of the phenotypic networks that was shared by all subjects and component sets. Consequently, health status provided a continuous metric by which to compare the effects of genotypic and environmental sets on the phenotypic networks.

We evaluated the effects of genotypic sets and environmental sets on the three networks to provide rough benchmarks of their relative contributions from a linear modeling perspective despite the limitations of such approaches. We estimated the effects of genotypic sets alone, environmental sets alone, and their joint effects in separate linear regressions on the phenotype specified as three phenotypic networks ordered by the values 1, 2, and 3 in correspondence to their mean levels of well-being. To standardize the unit of measurement for the different types of sets (genotypic, phenotypic, and environmental), we used the average well-being and/or ill-being status of their constituent sets of individuals. This provided a general and continuous characteristic of personality that could be compared for all subjects in each network (Supplementary Information: Section 10 and Tables S13 and S14). We used the *R*^2^ to compare the results obtained by the regressions, which were estimated by the average of tenfold cross-validation (Supplementary Information, Section 10). For ill-being, genotypic effects alone produced an *R*^2^ of 0.58, environmental effects alone an *R*^2^ of 0.27, and their joint effects an *R*^2^ of 0.68, showing the importance of both genotypic and environmental influences on ill-being. In contrast, for well-being, genotypic effects alone produced an *R*^2^ of 0.81, environmental effects alone an *R*^2^ of 0.23, and together an *R*^2^ of 0.82, so environmental influences did not add substantially to the explanatory effects of the genotypes for well-being. The details about specifying the phenotypic, genotypic, and environmental variables in terms of health status of genotypic sets, environmental sets, and phenotypic sets (temperament sets, character sets) is provided in the Supplementary Information (Section 10, Tables S13 and S14). Significance testing of the full model is detailed in Table S14.

### Replication of results in two independent samples

We tested the replicability of our findings in the Finnish sample by carrying out the same analyses of the genotypic and phenotypic architecture blindly in the German and Korean samples, thereby allowing for possible heterogeneity within and across independent samples from different cultures (Supplementary Information, Section 9). We evaluated the matching between each replication sample and the discovery sample of all aspects of the complex genotypic–phenotypic architecture (genotypic sets, phenotypic sets, and their relations) by a permutation test. Of the associations between genotypic sets and the phenotypic character sets identified in the Finnish sample, 84% were identified in the German sample and 94% in the Korean sample [18]. The associations between genotypic sets and the phenotypic temperament sets identified in the Finnish sample closely matched those observed in the Korean sample (89%) and in the German (76%) sample [19]. Finally, the replication between genotypic–phenotypic relationships when both temperament and character were considered were also replicated in the Korean (80%) and German samples (64%) (Supplementary Tables S15 and S16). The replication of the genotypic–phenotypic relations was reduced in the Germans (Supplementary Fig. S8), as expected because they were screened to exclude individuals with psychopathology, including personality disorders, as shown elsewhere [18, 19].

The strong replication across samples of all aspects of the complex phenotypic architecture of the networks (i.e., the matching of the temperament sets, character sets, and their relations in each replication sample to that of the discovery sample) was confirmed by a permutation test in both Koreans (1.0E−14 < *p* < 1.0E−02, Fisher’s exact test, Supplementary Table S17) and Germans (1.0E−11 *p* < 1.0E−02, Fisher’s exact test, Supplementary Table S18).

## Discussion

We have uncovered three robust findings about human personality for the first time. First, human personality is organized as a hierarchy of deep descriptive complexity ascending from (i) separate scales and subscales of temperament and of character, (ii) sets of multiple subscales of temperament and of character, (iii) profiles of temperament sets and of character sets, and finally to (iv) joint networks of temperament and character profiles. The genetic building blocks of the phenotypic hierarchy encode the temperament profiles and character profiles, not their constituent sets of personality subscales and not the three learning networks. Second, the three temperament–character networks are nearly disjoint phenotypes despite the marked complexity of temperament–character relationships within each of the three networks. Third, nearly disjoint sets of genetic and environmental variables strongly distinguish the three phenotypic networks. The strong association of both genetic and environmental variables with these integrated networks is surprising because the measured impact of the same environmental variables on the constituents of the integrated networks is weak, as we previously reported [18, 19]. Overall, we found that genes encode temperament profiles and character profiles separately, and then these are integrated by genetic–environmental interactions into complex adaptive networks.

These findings together suggest that the major role of environmental influences on personality development is the organization of the relations among temperament and character, not on the temperament and character profiles themselves that are largely genetically independent. Despite the novelty and surprising nature of these results, they are robust because we have strongly replicated the phenotypic and genotypic findings regardless of cultural and environmental differences among three independent samples of Finns, Germans, and Koreans. The replication was done in an unbiased fashion and avoided overfitting by balancing accuracy with model complexity while allowing for heterogeneity within and across independent samples, which is not necessarily the case for other GWAS methods [20] (see Supplementary Information, Polygenic Risk Scores). These findings have several important implications for both research and clinical practice.

### Importance of both genetic and environmental influences

Although genetic influences on temperament and character are substantial and robust, the impact of measured environmental influences has been weak [78–80]. The heritability of temperament and of character in the Finnish sample was 48% and 57%, respectively, whereas the influence of the environmental covariates did not exceed 3% when temperament and character were considered separately, as previously reported [18, 19]. Here we explored wider four-dimensional sets of interactions among temperament, character, genes, and environments that allowed for both direct associations and gene–environmental interactions associated with the phenotypic networks. This data-driven approach allowed us to identify many gene–environment interactions associated with the phenotypic networks, which would not have been detected in linear models of environmental influences on individual personality traits [20]. In contrast, prior work focused on individual traits rather than integrated profiles that characterize the organization of self-regulatory processes within a well-adapted person. This difference suggests that the major role of environmental influences is to interact with genetic predisposition in the activation of learning processes that result in integrated adaptive networks. For example, parental warmth and tolerance usually nurtures creativity in children (Fig. 4d), particularly those with genotypic sets for self-awareness and reliable temperaments (e.g., E_10_6 in Table 1), but it can also enable dependence in individuals with genotypic sets for emotional reactivity (e.g., E_8_8 in Table 1).

Specifically, we found that environmental variables had substantial influence on the risk of ill-being in particular; specifically, environmental and genotypic variables together explained an additional 10% of the phenotypic variance than did genotypic variables alone for ill-being (Supplementary Table S13). In contrast, genotypic variables had even larger effects on both ill-being (58%) and well-being (81%). Likewise well-being was most often high in people in the creative-reliable phenotypic network (see Fig. 3), which had the fewest proportion of associations with environmental sets (directly 1.8%, indirectly 5.3%) (Supplementary Table S12). Ill-being was frequently high in the other two networks; the organized-reliable network had the most environmental associations (directly 26%, indirectly 11%) and the emotional-unreliable network was intermediate (directly 0%, indirectly 13.3%) (Supplementary Table S12). These findings indicate people with creative-reliable profiles are usually resilient and healthy regardless of external conditions (Supplementary Table S3), whereas others are more vulnerable to stressful life events and social influences on disease and mortality, such as limited education and socioeconomic opportunity (Table 1) [18, 81, 82].

Both our work on personality [18, 19] and other work on the human exposome [83] converge on the idea that both environmental perturbations and the emotional reactivity of unregulated temperament networks lead to ill-health with multiple chronic noncommunicable disorders as a result of impaired regulation of gene co-expression, which has been called regulatory decoherence [84]. That is, environmental perturbations and the unhealthy character profiles found in the emotional-unreliable network impair the orchestration of gene co-expression, as has been most directly supported in other independent research by reduced correlations among sets of particular mRNA transcripts [84] and related psychophysiological processes that promote health, such as heart rate variability [85], rather than by variation in the presence or absence of particular genetic markers.

What are the molecular mechanisms by which genes related to personality may regulate the co-expression of genes that are far apart in the genome in coherent ways that are adaptive and beneficial for health? An important clue is that most of the genes associated with temperament are protein coding whereas those for character have a preponderance of noncoding RNA genes, particularly long noncoding (lnc) RNAs [18, 19]. LncRNAs have important functions that influence complex patterns of adaptive functioning and health by transcriptional and posttranscriptional regulation of gene expression, coordination of the co-expression of sets of genes, and chromatin remodeling [86–89]. A reasonable next step for further investigation is to examine patterns of co-expression of the personality related genes (both coding and noncoding) we have identified in blood and other tissues [84]. Then putative patterns of coherence versus decoherence can be tested for association with indices of health versus ill-being under a variety of conditions of physical, psychological and social stress, or other environmental perturbations. The indices of health may involve metabolic states, psychophysiological processes like heart rate variability, or other indices of the physical, mental, and social aspects of health. The indices of ill-being may involve disorders of various types (physical, mental, or social) with various patterns of comorbidity. Such investigations may clarify the roles of coding and noncoding genes in health and disease, as well as the mechanisms of associations of personality with ill-being and well-being via direct effects on gene expression and co-expression versus indirect effects via life style choice or environmental perturbation.

### Implications of three systems of learning for health promotion

We hypothesized that the disjoint sets of genes and environments associated with integrated temperament–character networks may correspond to the stepwise emergence of three different systems of learning and memory in the phylogeny of human beings [40, 44]. In other words, the integrated phenotypic networks are made up of people who express the prototypical features of each of three major systems of learning and memory present in modern human beings: associative conditioning for emotional reactivity in the Emotional-Unreliable network, intentional self-regulation in the organized-reliable network, and self-awareness of autobiographical memory in the creative-reliable network. Put another way, the three phenotypic networks are nearly disjoint prototypes of major systems of learning and memory, which are known to be neurologically and developmentally dissociable [17, 46].

Our findings that people with the creative-reliable profile were healthier than others confirm earlier work about how to describe a healthy human personality [70, 90–93]. We found that people with creative characters and reliable temperaments have greater well-being, including objective indicators of healthy longevity, such as optimal cardiovascular health, when compared with others. We also found that the genes for personality are expressed in most organ systems, not only the brain (Supplementary Fig. S9), so the physical, mental, and social aspects of health are expected to be strongly interdependent. Consequently, norms for healthy functioning need to consider the importance of self-transcendent functions, such as spontaneous creativity [94], altruism [95], and generosity [96], which are sometimes neglected [70, 97].

Our finding that healthy human functioning involves the integration of three systems of learning with qualitatively distinct properties [5] has strong implications for the modeling and use of artificial intelligence (AI) [98]. There is growing evidence that effective promotion of health and well-being depends on coordinated change in all three networks for self-awareness, intentional self-control, and behavioral conditioning, rather than any of these alone [99–102]. Optimizing the utility of AI for health promotion is likely to require it to recognize and address all three human learning systems in an integrative manner that facilitates its interaction with human beings in ways that are personalized to be satisfying, meaningful, and harmonious.

### Strengths and limitations

The strengths of this research are the use of unrestrictive data-driven methods, and replication of genotypic and phenotypic findings independently in samples that vary in their cultural and environmental features. A potential weakness of all clustering methods is that the number of clusters and their content are uncertain, but the methods adopted here addressed this carefully, as shown by the robust replication in independent samples [18–20]. We have shown that our methods are valid even in the presence of epistasis, pleiotropy, and heterogeneity, which cannot be accounted for by polygenic models that assume the independence of genes and environmental influences [20] (Supplementary Fig. S10). Our findings document the robustness of unsupervised machine-learning methods that allow the deconstruction and reintegration of the complex architecture of human personality.

Nevertheless, there are limitations to our study. We did not have comparable environmental data in the Korean and German samples, so only the genotypic and phenotypic findings were replicated here. The estimates of effect size used linear regressions on sets of variables, which captured interactions within the sets, but not between them. Also our findings are based on cross-sectional associations, so no causal inferences are justified.

## Conclusions and recommendations

Our data-driven findings support the hypothesis that human temperament and character are integrated as three nearly disjoint phenotypic networks regulated by complex interactions among nearly disjoint sets of genetic and environmental influences. Furthermore, these phenotypic networks are comprised of people who may express the prototypical features of three major systems of learning and memory that have previously been distinguished by their stepwise emergence in phylogeny and by their dissociable brain circuitry.

Our results show that the well-being of modern human beings depends on the maturation and integration of functions that interact to support healthy longevity, creative self-awareness, and self-transcendent functions such as moderation and altruism. In other words, physical, mental, social, and spiritual aspects of health cannot be separated because of the reciprocal interactions among the functions that support the well-being of the whole person. Mental or physical health cannot be adequately assessed, treated, or promoted as a set of separate diseases or traits.

## Supplementary information

Supplementary Information

Supplementary Figure S1

Supplementary Figure S2

Supplementary Figure S3

Supplementary Figure S4

Supplementary Figure S5

Supplementary Figure S6

Supplementary Figure S7

Supplementary Figure S8

Supplementary Figure S9

Supplementary Figure S10

Supplementary Table S1

Supplementary Table S2

Supplementary Table S3A

Supplementary Table S3B

Supplementary Table S4

Supplementary Table S5

Supplementary Table S6

Supplementary Table S7

Supplementary Table S8

Supplementary Table S9

Supplementary Table S10

Supplementary Table S11

Supplementary Table S12

Supplementary Table S13

Supplementary Table S14

Supplementary Table S15

Supplementary Table S16
