# Supplementary material for: Three genetic–environmental networks for human personality
Source: Mol Psychiatry. 2019 Nov 21;26(8):3858–75. doi: 10.1038/s41380-019-0579-x (PMC8550959; doi:10.1038/s41380-019-0579-x)
Supplement: Supplementary file 1 — Supplementary Information [file 41380_2019_579_MOESM1_ESM.pdf]

Supplementary Information

1. Description of the TCI scales and subscales
2. Comparability of TCI assessment in 3 samples
3. Pre-selection of SNPS
(i) The analysis of the covariate effect
(ii) Pre-discovery analysis of covariate effect performed by Plink
(iii) Post-discovery analysis of covariate effect performed by SKAT
4. Advantages of SNP-set analysis over alternative tests of single and multiple markers
5. The PGMRA method: Summary
6. The PGMRA algorithm
(i) Identify optimal genotypic and/or phenotypic sets (Implemented in the PGMRA web server [1]).
Mathematical description of the NMF
Transforming NMF k factors into sub-matrices or biclusters which are interpreted as SNP or phenotypic sets.
Decomposing the data into a multilevel family of sub-matrices
Learning the W and H matrices of FNMF
Optimally assembling the families of sub-matrices.
(ii) Perform a statistical analysis of SNP sets (accessed via the PGMRA web server [1]).
(iii) Calculate the frequency of subject status within a SNP set
(iv) Discover and encode relations among SNP sets into topologically organized networks
(v) Identify optimal and significant phenotypic sets (Implemented in the PGMRA web server [1]).
(vi) Identify optimal genotype-phenotype and/or phenotype-phenotype latent architectures
(vii) Bioinformatics analysis of the SNP set-related genes, their molecular consequences and pathways
7. Handling of missing values
8. Replicability of results
(i) Statistical replicability of sets of markers
(ii) Justification of the statistical replicability of sets of markers
9. Comparison between the PGMRA and the Polygenic Risk Score models
10. Estimation of relative contributions of genotypic and environmental effects using linear regression models
11. Prototypical vignettes for each network

1. Description of the TCI scales and subscales

Descriptors of high and low scorers on each of the subscales of the 7 TCI higher-order dimensions are presented in Table S1. All subscales were quantified as the average score per item in that subscale (so that 1 indicated an extreme low score, 3 a score near average, and 5 an extreme high score). For purposes of pattern recognition of profiles, medians for each subscale were calculated and those above the median were rated high and those below were rated low.

2. Comparability of TCI assessment in three samples

The Finnish longitudinal study involved 2,149 subjects (assessed in 1997, 2001, 2007, 2012), who completed the original TCI with 240 items using a 5-point Likert scale instead of the original true-false answer format [2, 3]. The averages of the scales and subscales scores across the four assessment occasions were utilized. The German subjects answered the same 240 TCI items using the original true-false format. Strong concordance between alternative formats in German and other languages has been previously demonstrated [4]. The Korean subjects used a short form made up of the 140 items with the strongest correlation with the full scales and using the same 5-point Likert format used in Finland [5, 6].

3. Pre-selection of SNPS

Our samples were treated as representative of the general population for initial analysis because when recruited they were not subdivided experimentally into unhealthy cases and healthy controls. To select an initial subset of SNPs and discard non-relevant SNP observations, we first cleaned the SNP data in the sample as described in [7]. Then, we selected SNPs with a high threshold (p-value<0.01) from a logistic or continuous regression calculated by Plink against the empirical temperament phenotypic index

described below (see below item (a) and Calculation of cluster rankings). The quality control (QC) of the genotypic data was performed following the steps detailed in references, removing consequently all the SNPs satisfying the following conditions: SNP call rate < 95% in either datasets, Hardy-Weinberg (HWE) p-value < 10E-6, Minor Allele Frequency (MAF) < 1%, and >1 discordant genotypes in either sample duplicates. To select the subject status for the regression, and because we did not have cases and controls, we calculated 3 indicators of variability in personality that could be estimated consistently in all three samples we analyzed (see below). Then, a subset of SNPs were pre-selected to reduce the large search space using the Plink software suite [8], taking sex and ancestry as covariates (see below), and using a generously inclusive threshold (p-value < 0.01) for association with temperament.

(a) Derivation of the empirical temperament/character index: First, we calculated a purely empirical (i.e., agnostic and data-driven) indicator of temperament functioning. We clustered subjects corresponding to the 12 temperament subscales and assigned each subject the number of the cluster to which they belonged (as described in the next paragraph). The result was a single empirical index of temperament cluster membership that served as a comprehensive measure of variability in temperament. We repeated process but clustering subjects corresponding to the 13 character subscales and deriving a single empirical index of character.

To calculate the cluster rankings we applied hierarchical agglomerative clustering (Statistical Toolbox, Matlab 2007b) with a complete linkage method and correlation similarity measurement to group SNP, phenotypic, or environmental sets by their shared subjects using hypergeometric statistics. The function that controls the vertical order in which a row is plotted (Spotfire Decision Site 9.1.2) in a hierarchical clustering is defined as follows.

Given two sub-clusters within a cluster (there are always exactly two sub-clusters considered at each step), both sub-clusters are weighted and the sub-cluster with the highest weight is placed above the other sub-cluster. This function is systematically applied until a single cluster containing all rows is obtained. To calculate the weight w_3 of a new cluster C_3 formed from two sub-clusters C_1 and C_2 with a weight of w_1 and w_2, and each containing n_1 and n_2 rows, the following expression is used:

$$w_3 = \frac{n_1 \times w_1 + n_2 \times w_2}{(n_1 + n_2)} \qquad \textit{\textbf{(eqn. 1)}}$$

The weight of a sub-cluster with a single row is calculated as the average value of its columns.

(b) Derivation of Dichotomous Indices of Well-being and Ill-being: Second, we calculated a partly theoretically-based indicator based on extensive prior work showing that high values on product of each of the three TCI character scales (that is, SDxCOxST) was associated empirically with positive health whereas low scores on the sum of Self-directedness (SD) and Cooperativeness (CO) was associated empirically with ill health. Specifically, we calculated well- and ill-being indices (see below second item) where subjects in the first and bottom deciles of SDxCOxST and SD+CO, respectively (see Tables S2A, B) were labeled as class '1' whereas the remaining subjects were labeled as class '0'. Thus, being extremely high or low was distinguished from being intermediate for pre-selection purposes.

(c) Derivation of Semi-quantitative Indices of Well-being and Ill-being: Third, we used the partly theoretical indicators to identify intermediate variation along with extreme variability in a semi-quantitative manner. As an extension of (b), we distinguished six equal-sized classes instead of a dichotomous classification (see Tables

S2A,B, e.g. 0-16% labeled as 1, 16-32% as 2, …). Extreme values 1 and 6 from analysis (c) contained most of the same subjects as were detected in the dichotomous (c) classification.

The empirical index calculated in (a) characterizes all the subjects, not only the extreme subjects. Moreover, this index is a single quantitative index, whereas the well and ill-being indices mixed individuals with either extreme high or extreme low well-being. Finally, the index in (a) can be re-calculated for Environmental variables and used as a measurement inherent to the sample, whereas the well- and ill-being indices are all based on Character subscales because prior research had established character is the self-regulatory component of personality, which largely accounts for differences between people with healthy (mature) and unhealthy (immature) adaptation.

(d) Application of the Indices: We used the empirical temperament index (i.e., procedure (a)) in the classification of SNP sets by SKAT. Note that SKAT for single SNPs provides a regression similar to that of Plink for individual SNPs [7]. Then, and independently, we tested the correlations between results when well- and ill-being indices were utilized in Plink and SKAT. The minimum p-value of all indices from (b) and (c) had a very strong correlation with the index from (a) (p value < E-20, RMSE 0.03). Thus our empirical temperament index and the well- and ill-being measurements are highly correlated indicators of personality variability, so that the empirical indicator was able to identify about 95% of the pre-selected SNPs associated with temperament.

(i) The analysis of the covariate effect

We accounted for ethnicity by using first three principal components (PCs) that account for ancestral stratification of SNP genotypes and sex [9, 10]. We took stratification into account both before and after the machine learning process. First, we accounted for covariates when selecting the initial set of individual input SNPs by using Plink [8]. Second, after selecting the constituents of the SNP sets, each SNP set was evaluated by SKAT [11, 12],

which accounts for covariates when sets of SNPs are assessed.

(ii) Pre-discovery analysis of covariate effect performed by Plink

Population stratification requires a covariate study before performing the statistical analyses. The magnitude of the effects of the population stratification depends on sample size, heritability, linkage disequilibrium structure and the number of causal variants [6]. Confounding bias occurs when a risk factor is also associated with the marker, as is observed when both disease and allelic frequency are correlated through ethnicity. This problem appears more markedly in the context of GWAS since these studies require a very large sample and are often carried out in different countries. Population stratification results in the inflation of p-values. This inflation can be detected and corrected for when testing for alleles that are associated with disease. The accepted way to correct for this effect is the use of covariates in the statistical analysis. It is important to limit the number of covariates because too many covariates can decrease the power of true detection [13].

In this study we performed an analysis of covariates based on Quantile-Quantile Plots (QQ Plots) and Lambda genomic control (λ) values to ensure the quality of adjustment [14, 15]. The QQ Plot is a graphical technique for determining if two data sets come from populations with a common distribution. If two sets come from a population with the same distribution, the points should fall approximately along a 45-degree reference line that is also plotted [16]. The λ is used to calculate the genomic inflation factor. The expected λ value is 1. If the value is greater than 1, then this may be evidence for some systematic bias that needs to be corrected in the study.

We tested the stratification effect by adjusting for gender and for the first 3 PCs (see preceding section on PCA analysis). Then, we calculated the lambda values and ran the QQ Plots (see [14, 15]As expected, the experiment without covariates ($\lambda = 1.363$) differs

from that one that considers all the covariates (λ = 1.094). It has been shown by different studies that the use of the first 3 PCs is usually sufficient to correct population stratification in GWAS studies [10].

(iii) Post-discovery analysis of covariate effect performed by SKAT

SKAT is a SNP-set level test for association between a set of rare (or common) variants and dichotomous or quantitative phenotypes. SKAT aggregates individual score test statistics of SNPs in a SNP set and efficiently computes SNP-set level p-values while adjusting for covariates, such as PCs to account for population stratification . For post-discovery analysis, we used 3 PCs previously described and gender as covariates (see [14, 15]SKAT also properly accounts for multiple comparisons [1, 11, 12].

4. Advantages of SNP-set analysis over alternative tests of single and multiple markers

In order to understand the novel design and methods of analysis of our study, it is useful to place it in the context of its strong advantages over the fundamental limitations of earlier methods in terms of their reproducibility, interpretability, and power. A long record of peer-reviewed work documents the advantages of SNP-set analysis in GWAS over alternative analyses of individual and multiple SNPs, particularly in adequately handling complex phenotypic-genotypic relationships influenced by epistasis and genetic variants in linkage disequilibrium (LD).

Analyses of associations with individual SNPs are often limited by poor reproducibility; that is, many of the highly ranked SNPs in the discovery phase are false positives and cannot be replicated or otherwise validated. This is largely due to the restricted power to detect SNPs with small effects that are truly associated with the outcome. Unfortunately, the individual SNPs that are genotyped on uncustomized GWAS platforms often show only modest effects with any particular phenotype. One explanation for this is that the true causal SNP is rarely genotyped but there are typed

SNPs that are in linkage disequilibrium (LD) with the causal SNP. In this case, when individual-SNP analysis is used, the typed SNPs in LD with the causal SNP will each show only weak or moderate effects because each typed SNP serves as an imperfect surrogate for the causal SNP.

Therefore, it could be advantageous to consider the joint effect of multiple SNPs in analysis [17] because it is probable that several of these markers are in LD with the causal SNP and could capture the true effect more effectively than could analysis of individual SNPs. Finally, individual-SNP analysis considers only the average and/or marginal effect of each SNP and therefore fails to accommodate epistatic effects even when in LD with the causal genes. Epistatic interactions between SNPs can contribute to disease susceptibility such that individual SNPs may show little individual effect but their joint interactions may have a much larger effect. Even exhaustive analysis of individual SNPs will not be able to detect such effects because the large number of potential interactions exceeds the statistical power of even extremely large samples [18]. As an alternative strategy for analysis, it has been proposed to group SNPs together into SNP sets along the genome and to perform genome-wide tests for SNP sets instead of individual SNPs. SNP-set-based analysis uses information from multiple correlated SNPs that are grouped on the basis of prior biological knowledge. As a result analysis of SNP sets has the possibility to provide improved reproducibility, interpretability, and increased power, especially when the effects of individual SNPs are weak or moderate [19, 20].

There have been several earlier attempts to describe multi-marker tests (that is, tests of multiple SNPs and/or other biological markers) to overcome the limitations of tests based on individual SNPs. The first class of multi-SNP test was based on individual-SNP analysis using the most significant p-value as the p-value for the set of loci, and then correcting for having done multiple tests [20]. However, such tests still rely

strongly on individual-SNP analysis, and when the individual SNPs are not in high LD with the causal variant, they may have low power and they cannot accommodate complex genetic effects and interactions.

Likewise extension of multi-marker tests for multiple SNPs or haplotypes via multivariate regression [21] often offer little benefit over methods based on individual-SNP analysis because they have a large number of degrees of freedom [22]. It has been proposed to compare pairwise genetic similarity with pairwise trait similarity to solve the problem of many degrees of freedom [23-25]. However, these tests still have major limitations: they assume that all variants have the same direction of effect, i.e., all the minor alleles for each SNP increase risk or all minor alleles decrease risk[25], or require expensive permutation analyses that may be impractical for some GWAS settings, or do not allow for easy covariate adjustment [25].

A second class of multi-marker tests consists of methods try to leverage explicit population-genetics models to pinpoint the causal locus. Many involve reconstructing the sample phylogeny to guide the analysis and infer the causal mutation [26, 27]. If the population-genetics model assumed is realistic and correct, such problem-specific methods should have high power. However, it is difficult to validate the assumed models, and most procedures are computationally intensive, such that in real applications the models need to be simplified. Once again, these models usually fail to allow for covariate adjustment. Computational efficiency and ease of covariate adjustment give a practical advantage to the logistic kernel-machine regression test over population-genetic modeling.

An alternative analytical strategy was proposed by Dr. Lin. She proposed to group SNPs together into SNP sets on the basis of proximity to genomic features such as genes or haplotype blocks, and then to test the joint effect of each SNP set. Testing of each

SNP set uses the logistic kernel-machine-based test[28], which is based on a statistical framework that allows for flexible modeling of epistatic and nonlinear SNP effects. This flexibility and the ability to naturally adjust for covariate effects are important features of our own test, which is an extension of Lin's approach that give it major advantages over individual SNP tests and existing multimarker tests. The logic behind our extension of Lin's analysis strategy is that we can extract information found in the joint relations of multiple SNPs to improve the power to detect true effects.

Consequently, the choice of the basis for grouping multiple SNPs can influence the power of the approach. Lin and her colleagues focused on grouping SNPs on the basis of their proximity to a known gene and noted that this allowed them to reduce multiple comparisons and to harness local LD structure in order to improve the power for capturing untyped SNPs. Using genes as the genomic features of interest allowed them [11, 12] to map approximately 310K SNPs to 18K SNP sets.

However, it may be that the causal SNP lies far from a known gene, in which case groupings based on genes (and, by extension, pathways) will fail to capture the effect of interest. To augment coverage of gene-desert regions of the SNP sets identified by Lin and others [11, 12], SNPs can be grouped on the basis of additional genomic features, such as evolutionarily conserved regions. Such groupings again may allow us to harness local correlation. A moving window approach will be useful for capturing all genotyped SNPs, but direct interpretation of SNP-set analysis results are more difficult. Groupings via haplotype blocks are attractive because they make explicit use of the LD information. Use of haplotype blocks will allow for comprehensive coverage of the entire genome and will remove the need to explicitly predefine genomic features of interest. Beyond harnessing local LD structure to boost power, another important feature of Dr Lin’s approach is the ability to model the joint effect of multiple, independent, causal signals as well as

possible epistatic effects.

Practically, however, finding a SNP-set formation strategy that optimize this approach can be difficult. It was suggested in [25] that using a gene or moving-window strategy can certainly capture multi-SNP and epistatic effects among SNPs that are located close to one another on the genome, but identification of such signals among SNPs that are distantly placed will not be possible. Indeed they suggested that a potential strategy would be to use prior biological knowledge. In particular, WU et al pointed out that if multiple SNPs are expected to affect the disease risk, it is reasonable to expect them to lie within genes in the same pathway or in genes with similar function; hence, forming SNP sets on the basis of pathways can potentially capture such effects. Unfortunately, WU et al concluded that a systematic approach for identifying such grouping structures at the genome-wide level is not obvious and that to avoid bias in our testing procedure, any grouping strategy must be made without consideration of the case-control status of the subjects in the data set. Wu et al said that groupings must be made with the use of information from external sources, prior studies, or unsupervised statistical methods [11, 12], and that SNP-set formation strategies will improve with advances in our knowledge of the genome and genomic structures.

5. The PGMRA method: Summary

Our approach implemented the suggestions that emerged from Dr Lin's work in order to uncover sets of multiple SNPs that may be correlated even at long distances on the same chromosome or on different chromosomes. We extended the approach in [11, 12] by using an "unsupervised statistical method" termed Phenotype-Genotype Many-to-many Relations Analysis (PGMRA, Figure S1). However, we used a purely data-driven method without biasing the search by using external sources, prior studies, or knowledge of the genome and genomic structures, such as genes, pathways, or additional genomic features, such as

evolutionarily conserved regions. Indeed, our SNP sets can share SNPs but not subjects, as expected because they involve the same SNPs but with different allele values (both alleles of a SNP can act as risk alleles in different genetic contexts) in different subjects [7]. Each SNP set was composed of a particular group of subjects described by a particular set of homozygotic and/or heterozygotic alleles; subjects and/or SNPs may be present in more than one set [1, 29, 30]. These SNP sets and their relations with one another characterize the genetic architecture of disease-associated SNPs in all subjects, including cases and controls.

Given a genotype database from a GWAS represented as a matrix of [SNPs x subjects] and a corresponding phenotype represented as a matrix of [features x subjects], the method for dissecting the architecture of a disease is composed of 6 steps. These steps are described for example elsewhere [7], where a SNP set is a sub-matrix or bicluster [1] harboring subjects described by a set of SNPs sharing similar allele values [1, 11], and the features are the TCI subscales. More generally, PGMRA uses a Generalized Factorization Method (GFM) to dissect a GWAS into SNP sets [1, 11] based on the Fuzzy Nonnegative Matrix Factorization method (FNMF) [1] algorithm (Figure S1B-C). FNMF is based on the bioNMF method [31], and uses it as a default basic factorization method (Figure S1D). FNMF allows detection of outliers and overlap among sub-matrices [1]. The GFM applies FNMF recurrently to generate multiple matrix partitions in each domain of knowledge (genotype and phenotype) using various initializations with different maximum numbers of sub-matrices k (where $2 \leq k \leq n$ and n is the number of subjects), and thus avoids any assumptions about the ideal number of sub-matrices.

Notably, the PGMRA method identifies local partitions of datasets, which provides substantial advantages over classical clustering approaches. Averaging and comparing groups would be expected to miss real differences if such differences are

localized in different locations, that is, in different subgroups of people with distinctive features (Figure S1F). In contrast to classical clustering techniques, such as hierarchical clustering [32] and k-means clustering [33], we used biclustering techniques that do not require subjects in the same bicluster to perform similarly over all features exhibiting changes. Classical clustering methods derive a global model whereas biclustering algorithms produce a local model in which signals emerge only in particularly relevant dimensions.

To guarantee that sub-matrices converge to the same solution and, given the non-deterministic nature of NMF and its dependence on the initialization of the *W* and *H* vectors, PGMRA runs the analysis 40 times for any *k* maximum number of allowed submatrices with different random initializations of the vectors to select those that that best approximate the input matrix [34]. To estimate the precision of sample statistics of the SNP sets (variance of the *W* and *H* vectors) we use a leave-one-out technique (jackknifing) 1000 times on the SNP domain, which produces more than 90% support for all identified sets with an average variance of approximately ±5% of their corresponding *W* and *H* vectors [31]. Finally, we modified the sampling technique to ensure the occurrence of the remaining sets after a leave-one-set-out procedure [35]. This sampling and estimation method was applied in the current study sample reported here with more than 90% of support.

By incorporating *a posteriori* the status of the subjects (see Table S2B) to SNP and phenotypic sets, the method is able to calculate the well-being and ill-being probabilities of such sets and their associations (see ***eqn. 5***, Map a disease risk in [7]). For example, as noted previously, the well-being status was defined by the top decile of *SDxCOxST* and the ill-being status was defined by the bottom decile of *SD*+CO based on independent prior empirical work. *Q* is the weights given by epidemiologic risk of a disease in each

SNP or phenotypic set (e.g., 0.01, 0.1 and 1 for cases, relatives and controls, respectively) [1], which here is ignored since subjects were from the general population and so generally considered more-or-less healthy. Once this procedure occurs, the method becomes semi-supervised, and posterior statistical significance of the SNP sets, and phenotype sets can be calculated using kernel-based and multivariate statistical analysis [1, 11]. We used conservative risk estimation for the associations between a SNP set and a clinical, which was calculated as the maximum value of both sets.

PGMRA co-clusters SNP sets with phenotype sets into associations calculating the probability of intersected subjects using coincident tests based on hypergeometric statistics (PI_{hyp}, [30, 36], equivalent to Fisher's exact test). The significance of the genotypic-phenotypic associations was tested by generating a permutation test, as described below and in [1, 37, 38]. All optimal relations had empirical p-value ≤ value < 5E-03. The probability of well- or ill-health for an association is calculated by the maximum probability of each related set (i.e., most conservative risk evaluation). These associations are organized into multilocus networks connected by sharing subjects and/or features (e.g., SNPs, symptoms), where shared SNPs between two SNP sets may differ in the allele values and have distinct genomic consequences [38-40]. This framework constitutes a knowledge base and characterizes the architecture of the phenotype. Further methodological descriptions of PGMRA are available in [1, 29, 35, 38, 41-43], and its web server application is online at http://phop.ugr.es/fenogeno [1]. Fast parallel software implementations were run at the Center for High Performance Computing (CHPC) facility at WUSM.

6. The PGMRA algorithm

PGMRA uncovers a deep architecture (Figure S1A) containing multiple sub-networks each uncovered by the NMF method used as a deep autoencoder[44] (Figure S1D) in a particular domain of knowledge (genetics, clinical symptoms, TCI, voxels in

neuroimages). Our implementation of the NMF, termed Fuzzy Nonnegative Matrix Factorization method (FNMF), learns and is optimized as described below and elsewhere[1, 38]. The nodes of one sub-network learned in a particular domain of knowledge (e.g., genetics) by a deep FNMF autoencoder (consensus clustering, see above) are connected by shared subjects and/or features (SNPs in genetics, subscales in TCI). Two sub-networks, learned from different domains of knowledge (e.g., genetics and TCI), are assembled by calculating the probability of intersection of their nodes and selecting those optimal connections based on multiobjective and multimodal optimization techniques (see above). These sub-networks constitute the pooling set of deeper layers of the network[45, 46]. Overall, the full network integrates different domains of knowledge into interpretable associative networks. The method utilized in this manuscript and described in (Figure S1B-C) is unsupervised because we want to extract new knowledge. However, it can be easily extended to a semi-supervised approach by adding a labeling and a classification layer [47] (Figure S1D). It should be noted that each layer has its own learning process (see below) and, instead of the weights in a neural network model, their outputs are interpretable relationships (Figure S1A,E).

Given a genotype database from a GWAS represented as a matrix [SNPs x subjects], the full method for dissecting the architecture of a disease is composed of 8 steps (Figure S1), where a SNP set is a sub-matrix [1], here also termed biclusters [34, 48], comprised of a subgroup of subjects described by a particular subgroup of SNPs sharing distinct allele values [1, 11]. In the current study, the phenotype database is composed of TCI subscales of temperament [TCI temperament scores x subjects] and TCI subscales of character [TCI character scores x subjects], and its analysis is approached in the same way as described for the genotype. Temperament, character and genotypic sets are independently learned from each other, but *a posteriori* associated into relationships.

As a convenient guide for readers, we will describe each of the steps in the PGMRA analysis as implemented in the PGMRA webserver and illustrated in Figure S1 in the following 7 sections (i to vii).

(i) Identify optimal genotypic and/or phenotypic sets

Mathematical description of the NMF (Figure S1D,E): We consider a dataset consisting of a collection of n subject samples, which we use to characterize a domain of genotypic (SNPs) or phenotypic (TCI) states of interest. Here, we illustrate the NMF by the genotypic type of data, but can be extended to any other type of data. The data are represented as an *m* x *n* matrix X, whose rows contain either the allele values of the *m* SNPs in the *n* subject samples. Using the NMF, we find a manageable number of factors *k*, positive local and linear combinations of the *n* subjects and the *m* SNPs, which can be used to distinguish the genetic profiles of the subtypes contained in the dataset. Mathematically, this corresponds to finding an approximate factoring, $X_{mxn} \sim W_{mxk} \times H_{kxn}$, where both matrices have only positive entries and hence are biomedicalli meaningful [1, 34, 49, 50]. *W* is an *m* x *k* matrix that defines decomposition model whose columns specify how much each of the subjects contributes to each of the *k* factors. *H* is a *k* x *n* matrix whose entries represent the SNP allele values of the *k* factors for each of the *n* subject samples. In our implementation either a subject or SNP can belong to more than one factor [1, 29, 30].

Transforming NMF k factors into sub-matrices or biclusters which are interpreted as SNP or phenotypic sets: The original bioNMF method [34, 48] uses the non-smooth variant of the NMF algorithm (nsNMF). This variant achieves an easier interpretation of the factors (*k*) due to the intuitive sparse, non-overlapped part-based representation of the data. Once the *W* and *H* matrices are calculated, the method selects the most representative features and observations (subjects) for each factor in order to build the biclusters. The bioNMF algorithm defines the factor-specific rows or columns as those rows or columns in the *H*

and *W* matrices, respectively, that show high coefficients for a given factor, as well as low coefficients for the other factors. Given a certain factor *k*, i.e., the lth column of *W*, all features in the dataset can be properly sorted by their association to the local pattern captured by this factor (Figure S1D(ii-iii)). At the same time, observations/subjects can also be sorted by their coefficients in the corresponding factor, that is, the lth row of H. This operation is carried out in one-to-one correspondence among columns of *W* and rows of *H*, generating *k* natural ordinations of the matrix in which features and subjects highly related in a sub-portion of the data. The set of selected rows and columns for each factor define a bicluster.

We developed a fuzzy variation of the bioNMF biclustering method named Fuzzy NMF or FNMF [1], where every column or row can belong to many biclusters or, eventually, to all of them [29, 30, 35]. In addition, our FNMF includes a strategy to identify and discard outliers from the biclusters, as in a possibilistic clustering method [35, 51, 52]. Unlike the bioNMF biclustering method, our FNMF analyses each factor by selecting the rows or columns with the highest values based on a threshold established as an input parameter. This threshold indicates the level of fuzziness, and in turn, which values will belong to a bicluster. The threshold is defined in the unit interval [0-1]. For example, the threshold for factor *i* in the matrix *H* is calculated as:

$$Threshold = max\ (\ H_i\ ) * (1 - fuzziness) \qquad \textbf{(eqn. 2)}$$

where all values above the threshold will be kept in the bicluster. The selection process for one factor takes into account only the values within that factor (that is, they are independent of the values of the rest of the matrix, Figure S1D(iii)). The fuzziness allowed in the current study was 30%. From now on, we will use the terms sub-matrix, bicluster, or set (SNP or phenotypic) as synonyms that serve to emphasize specific features of the same thing in different analytical or clinical contexts.

Decomposing the data into a multilevel family of sub-matrices (Figure S1E): The Generalized Factorization Method (GFM) applies a basic factorization method recurrently to generate multiple matrix partitions using various initializations with different maximum numbers of sub-matrices *k* (e.g., $2 \leq k \leq n$ where *n* is the number of subjects), and thus, avoids any assumption about the ideal number of sub-matrices (see [1] REF for a rationale about the use of unconstrained number of sub-matrices or clusters). Specifically, we use FNMF as described in the prior section. For each run of the basic factorization method ($2 \leq k \leq n$), all sub-matrices are selected to compose a family of genotypic SNP sets *G_k* ={G_*k*_*i*}, where $1 \leq i \leq k$. Each *G_k* family, as well as all families together *G* ={G_*k*} for all *k*, may include submatrices (i.e., sets) that are overlapping, partially redundant, and different in size.

Learning the W and H matrices of FNMF: Due to the non-deterministic nature of FNMF, it may not converge to the same solution on each run because of the random initial conditions used. Therefore, we execute the algorithm 40 times, as was originally suggested for the bioNMF algorithm [1], with different random initializations for selecting the *W* and *H* matrices that best approximate the input matrix. FNMF makes use of the convergence method described in [53, 54] to establish the stopping threshold that controls the algorithm convergence on each run. Each 10 iterations, a connectivity matrix *M* of size $C \times C$ is computed, where *C* is the number of columns of matrix *H*. Each entry M_{ij} in this matrix is set to 1 if column *i* and *j* in *H* have their maximum value for the same factor (i.e. on the same row in *H*), and 0 otherwise. If the connectivity matrix stops changing after a certain number of iterations (which equals the stopping threshold multiplied by 10), the matrices are considered as having converged and the algorithm stops the current run. The learning process of the *W* and *H* matrices is performed with projected gradient descent methods [53, 55].

Optimally assembling the families of sub-matrices. Because sub-matrices can be defined at different levels of granularity, we apply a competitive learning approach (i.e., consensus clustering [56, 57]) to select and assembly optimal, non redundant, and cohesive sub-matrices using multiobjective and multimodal optimization techniques. Optimal sub-matrices were obtained as a tradeoff between two opposing objectives: sensitivity and generality [29, 30, 38, 58-60]. Sensitive sub-matrices tend to be composed of few observations (i.e., subjects) described by multiple features, whereas specific sub-matrices are composed of many observations described by few features. A Pareto-optimization strategy searches for solutions that are non-dominated in the sense that there are no other solutions superior in all objectives being selected (i.e., close to the Minimum Description-Length (MDL) [61]). The dominance relationship as a minimization problem is defined as:

$$a<b\ iff\ \ \forall i\ Oia \leq Oib\ \exists j\ Oja \leq Ojb \qquad \textbf{(eqn. 3)}$$

where the O_i and O_j are either specificity or generality objectives. Optimization of small sets of sub-matrices was exhaustively implemented, whereas evaluation of large sets is approached by Genetic Algorithms, as described in [35, 43, 62]. Another indirect objective considered for the evaluation of sub-matrices is the generation of diverse patterns that completely describe objects (subjects). Therefore, our approach evaluates the sensitivity and generality objectives described above in a local niche [30, 58-60]. Both sensitivity and specificity measurements are based on counting objects within a sub-matrix without distinguishing among them (e.g., # subjects). However, diversity differentiates which objects are within a sub-matrix, and thus, sub-matrices harboring distinct objects are allocated in different niches. These niches are calculated using Jaccard's metric between sub-matrices [43, 62] (i.e., inclusion of subjects):

$$Niching(Bi,Bj)= S(Bi) \cap S(Bj) S(Bi) \cup S(Bj) > \gamma \qquad \textbf{(eqn. 4)}$$

where B_i and B_j were two different sub-matrices, the S functional retrieves the subjects in the sub-matrices in a particular niche, and γ is the size of the niche determined by the degree of overlap (i.e., intersection) between sub-matrices. Here the assumption is that the niches are equivalence classes dictated by the degree of overlap/inclusion between subjects in the sub-matrices. In sum, sub-matrices compete with each other if and only if they are in the same niche. For example, given two sub-matrices where one of them has the same or even worst sensitivity and generality than the other but correspond to different sets of subjects, both sub-matrices will be preserved because they are in different niches.

(ii) Perform a statistical analysis of SNP sets (accessed via the PGMRA web server [1]). Use the R-project package SKAT [11, 12] to evaluate the significance of each SNP set. We used identity-by-state (IBS) as a kernel because the analyzed variants are not rare but common, and therefore using the "weighted IBS" kernel would not be adequate [11, 12]. Since the SNP sets can overlap, we run each one separately. The gender and ancestry (3 PCs) of the subjects were used as covariates (see above), and the default remaining parameters were utilized. To run the SKAT test, we transformed the unsupervised SNP sets into a supervised form by labeling their subjects *a posteriori*. The ***global labeling*** of each subject assigns his or her case/control status, which in this case is the well-being or ill-being status. Then, the SKAT method evaluates the ability of each SNP set to classify the status of all subjects based on the SNPs included in such set. Another approach is termed ***local labeling***, which for each SNP set tags the subject within and outside that set with two different labels. Then, the SKAT method evaluates the probability of each SNP set to differentiate from the other sets. This is done because we hypothesize that all optimal SNPs are required to explain the distributed heritability [7].

The analysis of the SKAT that evaluates the ability of a SNP set to differentiate the

well-being and the ill-being status of all subjects is presented in the main text (Tables S4 and S5). In addition, for each SNP set we tested the probability of finding its component subjects and SNPs together. In other words, we evaluated how well the SNPs contained in a SNP set distinguished their subjects from the other subjects outside the SNP set or how different is a particular SNP set from the others. 95% of the SNP sets associated with the Temperament phenotype exhibited a 5E-08 > p-value > 5E-73 (13% < 5E-05). 95% of the SNP sets associated with the Character phenotype exhibited a 5E-11 > p-value > 5E-77 (5% < 5E-07). These results strongly support the importance of each individual SNP set as a contributor to explain the total distributed heritability.

(iii) Calculate the frequency of the subject status within a SNP or a phenotypic set

Once the health status of the subjects is incorporated *a posteriori*, the frequency of that status is calculated as a weighted average function of its observed epidemiological occurrence among all subjects in a particular set. Here, the probability of well- or ill-being status is defined as:

$$PX\_k\_i = i \in STStatusiQii \in STS \ \ dus \ \ i \qquad \textbf{(eqn. 5)}$$

where X_*k*_*i* is a SNP or phenotypic set, *Status* is the status of the instances, and *Q* is the weights given by the observed epidemiological risk of that status in each SNP set (e.g., 0 and 1 for controls and cases; 0.01, 0.1 and 1 for cases, relatives and controls, respectively) [1]. The frequency of the status for all SNP sets is interpolated as a surface using the *tgp* and *latticeExtra* packages in R-project, respectively.

(iv) Discover and encode relations among SNP sets into topologically organized multilocus networks

Co-cluster all SNP sets by calculating the pairwise probability of intersection among them using the Hypergeometric statistics [30, 36] (PI_{hyp}) on intersected SNPs: PI_{hyp} (G_*e*_*q*, G_*r*_*w*) (see below, ***eqn. 6***), where *q* and *w* are SNP sets generated in runs with a

maximum of *e* and *r* number of sub-matrices with the FNMF method. Two types of intersections are evaluated: SNPs and/or subjects. Connected and disjoint associations are organized into multilocus networks connected by sharing observations (subjects) and/or features (SNPs, symptoms), where SNPs shared by two SNP sets may differ in the allele values and have distinct genomic consequences [7]. (See multilocus networks in [63, 64].)

(v) Identify optimal and significant phenotypic sets (Implemented in the PGMRA web server [1]).

Next, we created a phenotype database by collecting the TCI temperament and character measurements at the sub-scale level encoded in the Likert scale. For efficiency and interpretability, in our case, each sub-scale variable was decomposed into two variables: the original variable *x* and the complementary *Lx* (Low x), which is 5-*x* in the Likert scale. The phenotype data was codified in a [phenotype features x subjects] matrix, where the columns and rows correspond to subjects and phenotypic features, respectively. To identify phenotype sets (as implemented in the PGMRA web server [1]) we apply the FNMF method with the phenotype database–instead of genotype database— as described above in Step 1, where a phenotypic set is a sub-matrix [1] harboring subjects described by a set of phenotypic features sharing similar values (i.e., *P_h_j*, where *j* is a phenotypic set generated in a run with a maximum of *h* number of sub-matrices). To select the optimal phenotypic sets, we applied the competitive learning process to the phenotypic sub-matrices as described above in Step 2.

The statistical evaluation of the phenotypic sets was performed in a fashion similar to that performed on the genotype. However, the SKAT test was replaced by a Chi-square test that evaluates a logistic regression with respect to the null model to get a p-value that reflects the ability of a phenotypic set to discriminate subjects by their status

(see *lrm* function of the R-package *rms*). In other words, the temperament phenotypic sets distinguish health of subjects (in terms of well-being versus) using a global logistic test: ~60% of the Temperament sets were significant discriminators of health status (1E-03 > p-value > 2E-20, and the remaining ~40% < 1E-02). Indeed, ~60% of the Character sets were significant discriminators of health status (1E-03 > p-value > 7E-120, and the remaining ~40% < 1E-02).

To encode the phenotypic sets into a manageable set of profiles or superclusters, we applied the FNMF method recurrently as a typical deep learning strategy (Figure S1E). Superclusters or profiles associate subsets of phenotypic sets, here termed temperament and character sets, at a lower level of granularity (i.e., detail) and represent semantic profiles that facilitate the communication of results without reducing the diversity of phenotypic sets encoded in such subsets. These superclusters are calculated by recurrently applying FNMF to the matrix encoding [phenotypic sets x TCI subscales]. The optimal number of sub-matrices was selected by the Cophenetic index [48] (see below deep NMF section, Figure S1E).

(vi) Identify optimal genotype-phenotype and/or phenotype-phenotype latent architectures

To identify genotypic-phenotypic relations, we co-clustered SNP sets with phenotypic sets into relations using the Hypergeometric statistics (PI_{hyp}, see below, ***eqn 6***) on intersected subjects, where $R_{i,j} = PI_{hyp}(G\_k\_i, P\_h\_j)$, G_k_i and P_h_j are SNP and phenotypic sets, respectively, and p is the intersection of subjects. Relations $R_{i,j} < T$ constitute the genotypic-phenotypic architecture of a disease. The significance of the relations (T) was established by the p-value provided by the Hypergeometric-based test [30, 36]. We proceeded in the same fashion to account for phenotype-phenotype relations between temperament and character sets.

The degree of overlap between two sets (SNP and/or phenotypic) was assessed by calculating the pairwise probability of intersection among them based on the Hypergeometric distribution [30, 36] (PI_{hyp}):

$$PI_{hyp}(P_i, G_j) = 1 - \sum_{q=0}^{p-1} \binom{h}{q}\binom{g-h}{n-q} \Big/ \binom{g}{h} \qquad \begin{array}{c} h = |P_i| \\ n = |G_j| \\ p = P_i \cap G_j \end{array} \qquad \textbf{\textit{(eqn. 6)}}$$

where *p* observations belong to a set P_i of size *h*, and also belong to a set G_j of size *n*; and *g* is the total number of observations. Therefore, the lower the $PI_{hyp,}$ the higher the overlap. The (p-value of) Hypergeometric "test" is used here as a measure of association strength. The real test (p-value) of genotypic-phenotypic relationship was provided through the permutation procedure.

The permutation test designed to evaluate the either genotypic-phenotypic or phenotypic-phenotypic relations was implemented as follows. Statistical significance values were obtained by 1000 independent permutations due to the comparisons between all possible generated sets (i.e., 1034, from 2 to n), and possibly overlapping sets were identified here as follows [1, 37]: a) assign random subjects to a genotypic cluster (set) of random size; b) assign random subjects to a phenotypic cluster of random size; c) calculate the PI_{hyp} between the two clusters and accumulate the value [36]. These values form an empirical null distribution of PI_{hyp} used to calculate the empirical p-value of an identified relation. All optimal relations had empirical p-value ≤ 5.00E-03. The phenotypic-phenotypic relationships between temperament and character sets were calculated in a similar fashion. (Note: the permutation test accounts for multiple comparisons [65].)

Here, the phenotypic-phenotypic associations were organized as networks (similar to genotypic-phenotypic associations). These associations are organized as networks by

applying the FNMF method recurrently as a typical deep learning strategy (Figure S1E). Network associate subsets of phenotypic sets, here termed temperament and character sets, at a lower level of granularity (i.e., detail) and represent semantic networks that facilitate the communication of results without reducing the diversity of phenotypic sets encoded in such subsets. The optimal number of networks was selected by the Cophenetic index [48] (see deep NMF section, Figure S1E).

(vii) Bioinformatics analysis of the SNP set-related genes, their molecular consequences, and pathways

For each SNP set, we analyzed all genes in the cluster, including the location of the SNP with respect to the gene, the type and number of genes comprising each SNP (e.g., distinguishing protein-coding genes, ncRNA genes, pseudogenes, and regulatory genes), the possible transcripts affected and the position where they are affected (e.g. coding region, distance to stop codon, splicing site, intron, UTR, etc.), and finally annotations about promoter and intergenic regions were inspected. All possible molecular consequences of each SNP in the function of the gene were considered in the analysis. A detailed analysis of SNPs and mapped genes can reveal at least three complex scenarios affecting multiple genes in different fashions (e.g. activation, repression, antisense modulation) and producing different molecular consequences, which were considered in queries of the Ensemble version 88 and NCBI databases (Entrezgene, Protein, Unigene). We evaluated whether a single SNP within a SNP set could produce different consequences in affected transcripts, whether multiple SNPs within a SNP set can jointly affect one or more genes in different ways, and finally whether multiple SNPs within different SNP sets can distinctively affect the same gene.

We investigated the regulatory and protein domain binding regions *de novo* using information available in known transcription factor databases and the sequences of the identified genes as inputs for our predictive bioinformatics algorithms [29, 30, 35, 43, 66-72], which perform novel predictions on genetic networks, RNA genes, and protein-protein interactions. Long non-coding RNA genes (lncRNA) were considered in particular because they were overrepresented among genes associated jointly with temperament and character. Omic data, including annotations about individual genes and families of genes were obtained from the Haploreg database [73], the Ensembl version 88, GeneALaCart, TRANSFAC ® release 2017.1, Pfam v30, and the NCBI web services. The RNomic analysis included the Linc2GO [74] and the LncVar [75] databases, and databases related to CircRNAs such as starBase v2.0, and circBase 0.1 [76, 77] in order to decode possible interactions among RNA genes. Once we obtained the information described above, we generated a list of relevant genes that were used to query the databases Nextbio, GeneALaCart v.4.5, DAVID v6.8, KEGG v82.0, Reactome v58, BioCyc v.21.0, WikiPathways, and Pathway Interaction Database [78-81] in order to identify pathways related to the genes. Overall, we found that the products of genes uncovered by the SNP sets are included in several well-known, relevant and interconnected signaling pathways. Annotation information obtained from Haploreg v4.1 [73], Ensembl version 88, NCBI, and GeneALaCart v.4.5, and TRANSFAC web services was manually curated.

7. Handling of missing data

The phenotypic values corresponding to the TCI scales have been calculated as the average of multiple longitudinal measurements and had no missing values. Missing values in the genotype were pre-processed using the Plink software suite [8]. The environmental variables were measured at various times during the study, as described in Supplementary Table S11. Then we grouped these variables into environmental sets composed of subjects sharing

environmental features (Table 1). Each set is not constrained to have all variables but only those that are shared by a group of subjects, and thus, do not need to be filled up by techniques to impute these values.

We carried out analyses to evaluate whether missing values in the raw data were missing at random. First, we carried out Little's Missing Completely At Random test in SPSS for all the TCI and environmental variables. This suggested that the missing values were missing at random, but we did not consider this decisive because of the restrictive assumptions of Little's test. Therefore, we created dummy variables for whether a variable is missing or not (by coding 1 = missing; 0 = observed). Then, we computed the Pearson correlations among the dummy variables. We found that the correlations among the dummy variables for missing values were low. Specifically, the average correlations of TCI dummy variables with the environmental dummy variables were between -0.1 and + 0.1, and vice versa with values less than 0.12. Given the observation of low correlations among the dummy variables for missing values, we conclude that the values missing in these variables are missing at random. Therefore the correction of these values by using corresponding measurements at other times is a reasonable imputation based on existing knowledge.

8. Replicability of results

(i) Statistical replicability of sets of markers phenotypes

PGMRA has been applied to each one of the three samples here considered: Finnish, Korean and German datasets. Replicability has been evaluated at three levels: 1) temperament sets; 2) character sets; and 3) temperament phenotypic-character phenotypic associations. SNP and temperament sets identified by PGMRA in the Finnish sample and replicated in the other samples were evaluated using Hypergeometric

statistics and the corresponding empirical distribution (see above, ***eqn 6***) with a PI_{hyp} threshold <1E-05 and <1E-02, respectively. Replication of a temperament set and character association across samples considers the replication of 1), 2), and the strength of the association (PI_{hyp}) of the homologous phenotypes sets in the target sample. Because all 3 measurements contributed to replicability, we selected the optimal replication associations by using multi-objective optimization techniques [7]. This approach is based on selecting those associations that are non-dominated. One association is non-dominated by others if there is no association that is better in all objectives (1,2, and the strength of 3) than the non-dominated association (see above, ***eqn. 3***).

(ii) Justification of the statistical replicability of sets of markers

In probability theory and statistics, the hypergeometric distribution is a discrete probability distribution that describes the probability of successes (random draws for which the object drawn has a specified feature) in draws, without replacement, from a finite population of size that contains objects with that exact feature, wherein each draw is either a success or a failure. In contrast, the binomial distribution describes the probability of successes in draws with replacement. In statistics, the Hypergeometric test uses the Hypergeometric distribution to calculate the statistical significance of having drawn a specific successes (out of total draws) from the aforementioned population. The test is often used to identify which sub-populations are over- or under-represented in a sample. This test has a wide range of applications in set theory and population studies [1, 29, 36].

The interpretation of the replication probability reflects the underlying stability of a group of observations or outcomes. In group theory and clustering in topology analysis [1, 29, 36, 82], this probability has been widely used to calculate stable clusters replicated by different methods, which helps to practically estimate the number of clusters in a data

partition, which is a theoretically unsolved problem [51, 52, 60]. Particularly in genetics, the probability of replication is used as an estimate of the probability that a gene set will be significantly expressed in a repeated study [82] or validated by sets of similar molecular information (co-clustering with enrichments [36], such as pathways and ontologies [1, 29, 36]). Both the hypergeometric (Fisher's exact test) and Wilcoxon tests are utilized to estimate the replication probability. The hypergeometric test takes the size of the overlap between a gene set and the list of differentially expressed genes as the test statistic, and reassigns labels without replacement (i.e. it keeps the marginal totals in the table constant). This is often used to calculate the significance for overrepresentation of the gene set among different sets of differentially expressed genes as a test for independence [65]. Specific examples of enrichment in human genetics include higher replication rates and consistently stronger enrichment of eQTLs [83].

The Hypergeometric analysis has served to logically focus replication efforts of SNPs that were found to be significantly associated in different samples or endophenotypes [84]. In such applications the method assumes that a given phenotype is influenced at each quantitative-trait locus (QTL) by one or more causal variant(s), whose effect(s) can be approximated (or tagged) by a linear combination of multiple semi-independent observed variants at the locus. This linear combination of SNPs is termed a multi-SNP. For example, the problems of allelic heterogeneity and imperfect tagging of multi-SNP associations and the identification of significant multi-SNP associations has been successfully addressed with the Hypergeometric test ([40], better tan Yang et al. method). These examples and others [1, 38, 85] suggest that the Hypergeometric test is a measurement that is useful for replicability analysis of sets of variables. Such analyses of sets of markers do not invalidate the use of meta-analysis of individual markers. A complementary alternative to the Hypergeometric is the permutation test (see preceding

section on Permutation test).

9. Comparison between the PGMRA and the Polygenic Risk Score models [86]

Following publication of our two articles [87, 88], a critique of the methodology of Phenotype-Genotype Many-to-Many Relations Analysis (PGMRA) [38, 87, 89] questioned the validity of our results from the perspective of polygenic risk scores (PRS) [90]. We appreciate the importance of these questions, and here provide a concise discussion of the assumptions and mathematical constraints of both approaches. We thank this commentator and others who have discussed our articles with us for their thoughtful questions and critiques.

Complex phenotypes present several challenges for genome-wide association studies (GWAS) including the presence of epistasis, pleiotropy, and heterogeneity. We approached these problems in a data-driven fashion to test the hypothesis that the heritability expected from twin studies but unexplained by genetic studies is distributed in heterogeneous partitions of a complex trait, each with distinct genotypic-phenotypic associations. We designed a machine learning algorithm termed PGMRA [38, 87, 89] to identify naturally occurring partitions in the data in an unsupervised fashion. PGMRA first dissects genome-wide data and uncovers a genotypic architecture composed of sets of SNPs shared by subsets of individuals (i.e., SNP sets [89, 91]). Next, phenotypic data are independently organized into natural sets of features such as clinical manifestations [7], voxels of neuroimages [85], or personality traits [87, 88] in a phenomic-like approach [92]. Cross-matching of the two types of sets reveals multiple associations restricted to subgroups of individuals, thereby uncovering the genotypic-phenotypic architecture of a trait and accounting for its distributed genetic risk or propensity.

Both approaches, PRS and PGMRA, rely on genome-wide markers (Figure S10).

However, PRS treat these markers as independent variables with additive effects, whereas PGMRA searches for sets of structurally connected markers, which may have interactive effects (epistasis). PRS assumes a global linear association model and relies on increasing sample size to improve performance [93, 94]. In contrast, PGMRA uncovers a family of models (i.e., SNP sets), each of which computes in a local partition of the data. Each model can be represented as either a linear combination of data (as in regression trees) or as a non-linear combination (as in some neural networks) [95]. Therefore, PGMRA uses a more complex model than PRS, focusing on incorporating more phenotypic variables rather than more individuals, but allows the use of smaller samples by reducing multiple comparisons.

PRS algorithms must reduce phenotypes to a single dependent variable because they use a linear supervised model [96]. In contrast, PGMRA uses an unbiased and unsupervised model to consider all possible phenotypic patterns common to a subset of individuals, regardless of their trait status (i.e., does not assign cases and controls *a priori*). Distinct patterns of phenotypic features can thus be associated with different SNP sets, thereby uncovering heterogeneous subtypes of the trait [38, 87, 88]. Finally, PGMRA incorporates trait status *a posteriori* to calculate the risk of such associations, and then independently tests the significance of the associations by a SNP-set Kernel Association Test [12, 91].

The validity of the replication procedure used by PGMRA was also questioned [90]. The "*gold standard*" approach used by PRS evaluates the reproducibility of an association by building a linear classifier trained in a *discovery* sample and testing it in a new sample assuming sample homogeneity [93, 94]. Homogeneity is a strong assumption that should be supported. By contrast, PGMRA uncovers genotypic-phenotypic associations for sample partitions and computes their corresponding risk or propensity *post hoc*; this process is

blindly repeated independently for each new sample without assuming homogeneity within or across samples (Figure S9). Then, similar genotypic-phenotypic associations across samples with comparable risk/propensity are uncovered using parsimonious models that balance accuracy with model complexity, thereby avoiding overfitting [95, 97, 98].

Inconsistent results obtained from applying PRS to heterogeneous samples [99, 100] has led to the suggestion of averaging scores from multiple samples [101] ignoring, at least in part, the phenotypic heterogeneity of the samples. When there is complexity derived from genetic, cultural, ethnic and environmental heterogeneity, the same global linear model is unlikely to predict across samples, especially when markers have relatively small effect [96, 99, 100]. Independently learned models from diverse samples, which allow analysis of replication across these samples, provide a more stringent test of reproducibility [102, 103].

PRS calculates heritability as an adjusted R^2 from a global linear regression, which additively estimates variance explained by the markers. In the absence of a validated estimator of variance for "sets" of markers [12, 91], PGMRA used a similar approach (Supplementary Figure S10). For example, the estimated heritability of character, without controlling for outliers and jackknife resampling, in the Finns sample [87] was 45.67%. A criticism [90] questioned the lack of application of another sampling technique such as cross-validation. As suggested, we applied cross-validation within and across samples (e.g., R^2 of 10 k-fold is 45.05% with SD 0.049) and confirmed the observed results by bootstrapping (1,000 iterations, SE <1.6%). We also found that the estimates of heritability for character in our paper [87] are conservative: the aggregation of the local variances explained by all SNP sets delivers a higher estimation of heritability ($R^2 > 15\%$) than the 45.67% described above (Figure S9, unpublished results).

Some suggest that our sample size (2,126 + 972 + 902 individuals from 3 cohorts, respectively [87, 88]) has insufficient power, even though others have calculated 80% power at nominal significance to detect heritability with the same sample size [96]. PGMRA computes genotypic-phenotypic associations based on "sets" of genotypes and "sets" of phenotypes, so the number of multiple comparisons are significantly reduced, making PGMRA less greedy of observations than PRS.

The nature of human beings embraces complex functions where every expressed gene may affect the function of any cell and their derived traits of our body in many different ways (many-to-many relationships). Complex traits are expected and known to be influenced by multiple genes acting in concert, not independently [104]. Most of the heritability in gene expression is determined by many genes far apart on the same or different chromosomes [104-106], whose effects are difficult to detect due to their small magnitude (e.g., trans eQTLs effects), as well as co-expressed genes that are vulnerable to decoherence in response to environmental perturbations [107]. PGMRA opens the door to develop new methods to explain complex genotypic-phenotypic relationships, including epistasis, pleiotropy and heterogeneous phenotypes, which present problems for PRS due to its restrictive linear model and doubtful assumption of homogeneity. Use of PGMRA would allow more thorough study of moderate-sized samples by efficient data-driven methods, which can help to bring methods of precision medicine into practice [85, 87-89, 103, 108].

10. ***Estimation of relative contributions of genotypic and environmental influences using linear regression***

Reviewers of earlier drafts of our manuscript suggested that readers would find it helpful to have a rough benchmark of the relative contributions of genotypic and environmental variables on the 3 networks we identified from the perspective of a linear regression model despite the limitations of such methods for complex phenotypes.

Therefore we have evaluated extent to which genotypic variables alone, environmental variables alone, and both genotypic and environmental variables jointly account for the variance in ill-being, well-being, and overall health status of subjects in a linear regression.

Every subject within a network has a specific set of characteristics, including their phenotypic sets (i.e, temperament set and character set), their genotypic sets, and their environmental sets. Therefore, to evaluate the influence of genotype and/or environment on phenotypic networks, we developed three linear multivariate regressions with the phenotype specified as the 3 networks ordered by the values 1, 2, and 3 in correspondence to their mean level of well-being (see results in main article where means and standard deviations for each network are given). The genotypic and environmental variables were represented as groups of variables (i.e., genotypic sets and/or environmental sets). To standardize the unit of measurement for the different types of sets (genotypic, environmental, phenotypic), we used the average health status (well-being, ill-being, or composite health index) of their constituent sets. This provided a general and continuous characteristic of personality that could be compared for all subjects in each network. We estimated the effects of genotypic sets alone, environmental sets alone, and their joint effects using the following equations:

$N1(x)=c1\ GCx+c2GTx+c3GCTx$

$N2(x)=c4ECx+c5\ ETx+c6ECGx+c7ETGx+c8\ ECTGx$

$N3(x)=c1\ GCx+c2GTx+c3GCTx+c4ECx+c5\ ETx+c6ECGx+c7ETGx+c8\ ECTGx$

where the functions *XY()* retrieve the average group value of the ill- or well- being status, and C=character set, T=temperament set, G=genotypic SNP set, E=environmental set.

We evaluated the results of the equations by calculating the R^2 as the average of 10-fold cross-validation application of the regression model (Note: that the estimates of these regressions are not additive). From the values for R^2 in ill-being explained by G, E, and both in Table S13, we conclude that both genotypic and environmental variables influence ill-being (that is, 68% for joint genotypic and environmental effects > 58% for genotypic effects alone). However, for well-being and overall health status, the combination of genotypic and environmental effects is not substantially greater than genotypic effects alone (82% is close to 81%, 90% is close to 88%). We show detailed estimates of coefficients in the full model with both genotypic and environmental variables in Table S14 using the stepwise procedure in Matlab R2017b).

11. Prototypical vignettes for each network

(1) Emotional-unreliable Network Prototype

"Surullinen" (Finnish for SAD): Sensitive-Apathetic profile (TCI profiles C_3_2 and T_3_3) with Disorganized attachment [difficult child reared by highly educated parents without much acceptance or tolerance (E_13_10/E_152) in rural environment (E_6_2)] and associated with G-Protein-Coupled Receptor dysregulation (SNP set G_7_2)

Surullinen (Finnish for sad) is a 45- year old unmarried woman who is disabled by multiple medical and psychiatric disorders associated with marked stress reactivity and apathy. These include Borderline Personality Disorder, Persistent Depressive Disorder, Substance Use Disorder, Fibromyalgia, Obesity, type-2 Diabetes Mellitus, Hypertension, and Rheumatoid Arthritis.

Rearing Environment: Her parents are highly educated professionals with responsible jobs and good income working in a rural area. Surullinen had prominent negative emotionality as a child (i.e., she was easily upset). Her parents and teachers felt she had a difficult temperament because she was so easily distressed, irritable, distractible, hyperactive and hard to motivate. She felt inadequate and was afraid of her father's harsh discipline but was enabled to be irresponsible by her mother. Her parents divorced when she was 11 years old. She and her two older siblings stayed with her mother, but she always felt excluded by her siblings.

Psychosocial development: When she started school she made few friends and was often teased for being overweight. Her school performance was erratic and low average overall, which was inferior to that of her parents and siblings.

As a teenager and adult she continued to be emotionally unstable, impulsive, and to fear abandonment. At 17 she was hospitalized following a suicide attempt after breaking up with an abusive boyfriend. She entered college but quit after one year when she

became pregnant. The child's father took custody of their child due to her neglect. She moved to a large city where she began gambling, smoking, drinking alcohol heavily, and using illicit drugs occasionally.

Throughout her adulthood her Temperament and Character Inventory showed she had a sensitive temperament: she was very high in Harm Avoidance (pessimistic, fearful, shy, and fatigable), high in Reward Dependence (sentimental, open, and forming intense but unstable attachments), high average in Novelty Seeking (extravagant and impulsive), and low in persistence (easily discouraged, lazy, underachieving). She also consistently reported an apathetic character profile: low Self-directedness (irresponsible, aimless, helpless, hopeless, and unable to regulate her bad habits with overeating, gambling, abusive relationships), low Cooperativeness (intolerant, self-serving, hostile, revengeful, and opportunistic), and low Self-transcendence (demanding, self-preoccupied, and skeptical). Her mother managed her financial support because she gambled and spent whatever money came into her possession.

<u>Physical Health Functioning</u>: Throughout adulthood she has been obese (BMI 31 to 36) along with documented type-2 diabetes mellitus, hypertension, and rheumatoid arthritis. She complained of multiple bodily pains, gastrointestinal problems, psychogenic seizures, and painful menses, leading to extensive treatment, including a hysterectomy and cholecystectomy. She had frequent infections and slow healing following injuries.

<u>Treatment</u>: When last evaluated she was disabled by her many psychiatric and medical complaints, including objective problems with Diabetes mellitus, hypertension, rheumatoid arthritis, and impaired immune function. She has been seen often but irregularly for treatment of Borderline Personality Disorder and persistent depression with medications and supportive psychotherapy without benefit except short-term crisis-relief. She regards herself as a victim and does not accept responsibility for change.

Genetic Predisposition: She carries a large SNP set associated with G-protein-coupled receptor dysregulation (G_7_2). This SNP set was associated with 23% risk of ill-being in 211 people who carried it. The SNP set was associated with both the sensitive temperament and the apathetic character. The 88 protein-coding and 64 other genes mapped to this SNP set were distinguished by multiple processes associated with stress reactivity and apathy. Specifically, 13 genes involved GPCR dysregulation associated with stress reactivity and susceptibility to hypertension, atherosclerosis, heart failure, and diabetes. Another gene is a growth factor (PDGFB) that influences neuronal development and migration via activation of Ras-ERK, PI3K/AKT, and PKC signaling; its effects on Ras-ERK regulates neuroplasticity in the striatum, which in turn modulates interactions between frontocortical and mesolimbic signaling, including effects on dopaminergic and vagal neuronal activity known to induce anxiety and apathy. Another gene for a component of an ATPase (ATP6V1A) is involved in mTOR signaling, maintenance of homeostasis and environmental reactivity in the HPA axis, insulin receptor recycling, immune reactivity, and susceptibility to rheumatoid arthritis. Another 7 genes influence stress response via cAMP activation of PKA and reduce energy availability via RNA-mediated gene silencing of fatty acid oxidation for entry into the Krebs cycle.

(2) Organized-Reliable Network Prototype

"Ohjautuva" (Controlled in Finnish)-- Organized-conventional profile (TCI profiles C_8_7 and T_6_1) associated with SNP set for Inositol/Chemokine pathways (G_8_8) and environmental sets for high parental income (E_14_3) and high parental education (E_15_2).

Ohjautuva is a 45- year old divorced man who is an acquisitions manager for a large manufacturing company and a reserve officer in the Finnish military force. He is a

university graduate and an avid sportsman like his father. His health is slightly better than average with no major chronic illnesses. He intermittently feels stressed by work and by child-rearing issues since his divorce, but has not sought counseling. He takes a beta-blocker for mild hypertension exacerbated by stress and frustration, and a statin medication to reduce his high levels of cholesterol and triglycerides.

Rearing Environment: Ohjautuva was reared in a small city by two well-educated parents who were both white-collar workers. He has a sister who was six years older than him. He described his childhood as happy and particularly enjoyed activities with a group of several boys in his neighborhood. They liked playing outdoor sports together. His parents both worked long hours, but as a schoolboy he enjoyed skiing and fishing with his father on weekends.

Psychosocial Development: Ohjautuva's early development was normal. He was an obedient well-behaved child. He kept his room neat and orderly. In school he was a good but not exceptional student. He was avid about popular outdoor sports, including trekking, cross-country skiing, and canoeing. From the age of 7 he and his father would ski and fish together, which they continue to do.

After completing high school at age 18, he entered into the Finnish military service where he excelled. He stayed in service for 9 months for officer training, and later joined an officer club that had regular celebrations and a close-knit social network. Then he completed university with a major in business administration. He obtained employment in a large manufacturing company, and has done well there. He is resourceful in acquisition and distribution of supplies for the manufacturing company, rising to a mid-level management position because of his dependability.

Ohjautuva got his girlfriend pregnant when he was 35 years of age. She was religious and insisted on having the child. He married the mother, but she was never

happy about his zeal about hunting and military operations. She divorced him after 5 years. His son is now 10 years old and his wife disapproves of Ohjautuva wanting to take the boy skiing and fishing. This is stressful to Ohjautuva who wants to introduce his son to activities that he and his own father enjoy.

Throughout his adulthood, his TCI showed Ohjautuva had an organized character profile. He was very high in Self-directedness (i.e., responsible, purposeful, resourceful, and self-controlled), very high in Cooperativeness (tolerant, helpful, and principled), and low in Self-transcendence (defensive, individualistic, and skeptical about religious and spiritual beliefs).

Ohjautuva's temperament profile was broadly described as reliable. More specifically his temperament involved his desire to adhere to the conventions and traditions of his community. He was consistently very high in Reward Dependence (warm attachments, pleasing others), very low in Novelty Seeking (frugal, orderly, disliking breaking rules and conventions), and very low in Harm Avoidance (optimistic, outgoing). He was slightly above average in Persistence.

Genetic Predisposition: SNP set G_8_8 is comprised of genes involving Inositol/Chemokine Pathways and is associated with both his Character and Temperament sets (C_8-7 and T_6_1). It involves 661 snps in 224 subjects. These SNPs map to 291 genes, 67% of which are protein-coding. The constituent genes are distinguished by 41 genes that regulate long-term learning and memory related to intentional goal-directed behavior and social reconciliation. For example, they regulate long term learning about responses to environmental signals regarding opportunities for obtaining energy from food, including 11 genes for inositol/Calcium second-messenger signaling (e.g., ITPR2, PIK3R1, PLCB4), 5 for glucose/energy metabolism (e.g., ABCC4), 11 for hormonal signaling (e.g., CREB5, GNAO1), and 10 for calcium-mediated release of

inositol-triphosphate-derived second-messengers at cholinergic synapses (e.g., PLCB4, CHRM3). Five genes regulate energy output and promote self-confidence following stress (e.g., PLCB4, ITPR2, BMP7, SHH) via gonadotrophin-releasing hormone and hedgehog signaling pathways. SNP set G_8_8 includes another 35 genes that regulate aging, stress reactivity, pain and inflammation (e.g., COL2A1, ITGAV) via interactions of calcium signaling with chemokines and extracellular matrix proteins.

(3) Creative-Reliable Network Prototype

"Luova" (Creative in Finnish): Creative-reliable personality profile (TCI profiles of C_8_8 and T_5_1) associated with SNP sets for episodic learning (G_12_1) and enhanced memory (G_20_2) but no environmental sets.

Luova is a 45-year old married woman who works professionally as an artist (painting and sculpture). She is a university graduate with her Masters degree in art. She is in excellent health physically, emotionally, and socially.

Rearing Environment: Luova she was reared in a stable home in rural Finland by fairly well-educated parents. Her father was a skilled worker in the forestry industry and her mother was a homemaker caring for Luova and her 4 younger brothers. Luova stood out for her positive emotionality from an early age. By age 4, her parents recognized that she loved singing and music, so they helped her cultivate her natural talents. By age 7 she began studying piano and singing. She loved for her mother to read children's stories to her and her brothers. At age 8 she began to read stories to her brothers. Luova was active, inquisitive, and had many friends. She often helped and protected her friends and siblings.

Psychosocial Development: In school she did well but needed extra challenges to keep from being bored and talking too much. She excelled in art and music. Her vivid imagination stood out. For example, in her early teens she taught her friends a story-

telling game that she devised: they would observe someone carefully, describe them in detail, and then invent an imaginary life story to explain the observations. The friends had fun trying to tell the most interesting story that explained the facts.

She was not afraid to take risks, so in her late teens and early adulthood she experimented with parachuting and other thrilling activities. She gave up risky activities when she recognized the danger. She never experimented with illicit drugs. Throughout her adulthood, her TCI showed Luova was highly self-directed (resourceful, purposeful, responsible, and self-actualizing, but not unusually self-accepting). She was highly cooperative (tolerant, helpful, empathic, and compassionate). She was not unusually principled according to cultural conventions, but instead depended on her own intuitions about what was fair. She reported being easily absorbed when reading, painting, or singing (ST1). She reported recurring joyful experiences in which she felt she had an inseparable connection with nature and other people (ST2), which often inspired her art. Although she was not religious, she liked nature walks in which she would find inspiration and often experienced a spiritual connection that felt sacred to her (ST3). Luova's temperament throughout her adulthood was broadly described as reliable because she reported being very low Harm Avoidance (i.e., optimistic, unafraid of uncertainty, outgoing, highly energetic and resilient), high in Reward Dependence (i.e., sentimental with warm attachments, but not approval seeking), and high in Persistence (i.e., eager, determined). In addition, she was high in some aspects of Novelty Seeking (i.e., exploratory, inquisitive, extravagant, but not impulsive or disorderly). Consequently, her temperament can be described as both reliable and exploratory.

Luova has been stably and happily married for 16 years and has two teenage children. She and her husband settled in an urban area where they had attended university. She is a successful artist noted for the way her paintings communicate a story.

She has a rich spiritual life with a regular meditation practice. She is very engaged in her community and regularly mentors students in art.

Genetic Predisposition: Luova carried both the SNP sets G_12_1 for episodic learning (G_12_1) and enhanced memory (G_20_2), which are associated with both her temperament and character profiles. SNP set G_12_1 involves 189 SNPs in 146 subjects, and maps to 64 genes, 61% of which are protein-coding. The genes are distinguished by their regulating gene expression in the brain regions making up the circuit for episodic learning and memory. Specifically, 5 genes regulate gene transcription and energy production in the brain circuit for autobiographical learning (CAMTA1, CCDC39, IDS, NRXN1, and SLC14A2). Two of these genes and 6 others regulate neurogenesis in the hippocampal formation (i.e., PGLRP4, RUNX1, CDKL1, DCC, MAGI2, PGLRP4).

The second SNP set for enhanced memory (G_20_2) has 80 SNPs in 25 subjects. Its SNPs mapped to 19 genes, 79% of which are protein coding. It is distinguished by all of its 15 protein-coding genes being involved in enhancement and protection of learning and memory, including 10 genes overexpressed in brain. These genes include 6 that regulate synaptic plasticity and enhance learning and memory: cAMP responsive binding protein (CREB) is the primary hub of gene transcription for neurotransmitter-activity-driven programs controlling synaptic plasticity, neurogenesis and survival. In neurons, CREB regulates synaptic plasticity to enhance excitatory synaptic transmission (with FRMPDR, KCNG3, MS4A3, OXER1) and short-term synaptic plasticity (with SHISA6). In astrocytes, CREB is neuroprotective, influencing mitochondrial function and gene expression. Memory is also enhanced by coordination of histone and nucleosome remodeling (KDM4D, SETBP1, MTA3). Further neuroprotection is provided by energy production in brain regions for autobiographical memory (COX7A2L) promoted by mitochondrial biogenesis from AKT signaling (BMPR1B) and protected from DNA damage by SMG1.

This SNP set also has genes influencing axon guidance (UNC5C), neurite outgrowth (BMPR1B), microtubule assembly dynamics influencing learning and memory (EML4), and post-translational modification reported to facilitate social identification (PARP8).

References

1. Arnedo J, del Val C, de Erausquin GA, Romero-Zaliz R, Svrakic D, Cloninger CR *et al.* PGMRA: a web server for (phenotype x genotype) many-to-many relation analysis in GWAS. *Nucleic Acids Res* 2013; **41**(Web Server issue)**:** W142-149.

2. Raitakari OT, Juonala M, Ronnemaa T, Keltikangas-Jarvinen L, Rasanen L, Pietikainen M *et al.* Cohort profile: the cardiovascular risk in Young Finns Study. *Int J Epidemiol* 2008; **37**(6)**:** 1220-1226.

3. Ravaja N, Keltikangas-Jarvinen K. Cloninger's temperament and character dimensions in young adulthood and their relation to characteristics of parental alcohol use and smoking. *J Stud Alcohol* 2001; **62**(1)**:** 98-104.

4. Brandstrom S, Richter J, Nylander P-O. Further development of the Temperament and Character Inventory. *Psychological Reports* 2003; **93:** 995-1002.

5. Sung SM, Kim JH, Yang E, Abrams KY, Lyoo IK. Reliability and validity of the Korean version of the Temperament and Character Inventory. *Compr Psychiatry* 2002; **43**(3)**:** 235-243.

6. Yang SS, Sung J, Kim J-H, Song Y-M, Lee K, Kim H-N *et al.* Some personality traits converse gradually by long-term partnership through the lifecourse: Genetic and environmental structure of Cloninger's temperament and character dimensions. *Journal of Psychiatric Research* 2015; **63:** 43-49.

7. Arnedo J, Svrakic DM, del Val C, Romero-Zaliz R, Hernández-Cuervo H, Molecular Genetics of Schizophrenia Consortium *et al.* Uncovering the Hidden Risk Architecture of the Schizophrenias: Confirmation in Three Independent Genome--Wide Association Studies. *The American journal of psychiatry* 2015; **172**(2)**:** 139-153.

8. Purcell S, Neale B, Todd-Brown K, Thomas L, Ferreira MA, Bender D *et al.* PLINK: a tool set for whole-genome association and population-based linkage analyses. *American journal of human genetics* 2007; **81**(3)**:** 559-575.

9. Prado-Martinez J, Sudmant PH, Kidd JM, Li H, Kelley JL, Lorente-Galdos B *et al.* Great ape genetic diversity and population history. *Nature* 2013; **499**(7459)**:** 471-475.

10. Price AL, Patterson NJ, Plenge RM, Weinblatt ME, Shadick NA, Reich D. Principal components analysis corrects for stratification in genome-wide association studies. *Nat Genet* 2006; **38**(8)**:** 904-909.

11. Wu MC, Kraft P, Epstein MP, Taylor DM, Chanock SJ, Hunter DJ *et al.* Powerful SNP-set analysis for case-control genome-wide association studies. *American journal of human genetics* 2010; **86**(6)**:** 929-942.

12. Wu MC, Lee S, Cai T, Li Y, Boehnke M, Lin X. Rare-variant association testing for sequencing data with the sequence kernel association test. *American journal of human genetics* 2011; **89**(1)**:** 82-93.

13. Pirinen M, Donnelly P, Spencer CC. Including known covariates can reduce power to detect genetic effects in case-control studies. *Nat Genet* 2012; **44**(8)**:** 848-851.

14. Zwir I, Arnedo J, Del-Val C, Pulkki-Råback L, Konte B, Yang SS *et al.* Uncovering the Complex Genetics of Human Character.. *Molecular Psychiatry* 2018.

15. Zwir I, Arnedo J, Del-Val C, Pulkki-Råback L, Konte B, Yang SS *et al.* Uncovering the Complex Genetics of Human Temperament.. *Molecular Psychiatry* 2018.

16. Voorman A, Lumley T, McKnight B, Rice K. Behavior of QQ-plots and genomic control in studies of gene-environment interaction. *PLoS One* 2011; **6**(5)**:** e19416.

17. Schaid DJ, Rowland CM, Tines DE, Jacobson RM, Poland GA. Score tests for association between traits and haplotypes when linkage phase is ambiguous. *American journal of human genetics* 2002; **70**(2)**:** 425-434.

18. Hunter DJ, Kraft P. Drinking from the fire hose--statistical issues in genomewide association studies. *N Engl J Med* 2007; **357**(5)**:** 436-439.

19. Liu D, Ghosh D, Lin X. Estimation and testing for the effect of a genetic pathway on a disease outcome using logistic kernel machine regression via logistic mixed models. *BMC Bioinformatics* 2008; **9:** 292.

20. Lin DY. An efficient Monte Carlo approach to assessing statistical significance in genomic studies. *Bioinformatics* 2005; **21**(6)**:** 781-787.

21. Zaykin DV, Westfall PH, Young SS, Karnoub MA, Wagner MJ, Ehm MG. Testing association of statistically inferred haplotypes with discrete and continuous traits in samples of unrelated individuals. *Hum Hered* 2002; **53**(2)**:** 79-91.

22. Chapman JM, Cooper JD, Todd JA, Clayton DG. Detecting disease associations due to linkage disequilibrium using haplotype tags: a class of tests and the determinants of statistical power. *Hum Hered* 2003; **56**(1-3)**:** 18-31.

23. Schaid DJ, McDonnell SK, Hebbring SJ, Cunningham JM, Thibodeau SN. Nonparametric tests of association of multiple genes with human disease. *American journal of human genetics* 2005; **76**(5)**:** 780-793.

24. Wessel J, Schork NJ. Generalized genomic distance-based regression methodology for multilocus association analysis. *American journal of human genetics* 2006; **79**(5)**:** 792-806.

25. Mukhopadhyay I, Feingold E, Weeks DE, Thalamuthu A. Association tests using kernel-based measures of multi-locus genotype similarity between individuals. *Genet Epidemiol* 2010; **34**(3)**:** 213-221.

26. Minichiello MJ, Durbin R. Mapping trait loci by use of inferred ancestral recombination graphs. *American journal of human genetics* 2006; **79**(5)**:** 910-922.

27. Tachmazidou I, Verzilli CJ, De Iorio M. Genetic association mapping via evolution-based clustering of haplotypes. *PLoS Genet* 2007; **3**(7)**:** e111.

28. Tzeng JY, Zhang D. Haplotype-based association analysis via variance-components score test. *American journal of human genetics* 2007; **81**(5)**:** 927-938.

29. Zwir I, Shin D, Kato A, Nishino K, Latifi T, Solomon F *et al.* Dissecting the PhoP regulatory network of Escherichia coli and Salmonella enterica. *Proc Natl Acad Sci U S A* 2005; **102**(8)**:** 2862-2867.

30. Zwir I, Huang H, Groisman EA. Analysis of differentially-regulated genes within a regulatory network by GPS genome navigation. *Bioinformatics* 2005; **21**(22)**:** 4073-4083.

31. Schachtner R, Lutter D, Knollmuller P, Tome AM, Theis FJ, Schmitz G *et al.* Knowledge-based gene expression classification via matrix factorization. *Bioinformatics* 2008; **24**(15)**:** 1688-1697.

32. Sokal RR, Michener CD. A statistical method for evaluating systematic relationships. *University of Kansas Science Bulletin* 1958; **38:** 1409-1438.

33. Hartigan J, Wong M. Algorithm AS 136: A K-means clustering algorithm. *Applied Statistics* 1979**:** 100--108.

34. Pascual-Montano A, Carazo JM, Kochi K, Lehmann D, Pascual-Marqui RD. Nonsmooth nonnegative matrix factorization (nsNMF). *IEEE transactions on pattern analysis and machine intelligence* 2006; **28:** 403-415.

35. Harari O, Park SY, Huang H, Groisman EA, Zwir I. Defining the plasticity of transcription factor binding sites by Deconstructing DNA consensus sequences: the PhoP-binding sites among gamma/enterobacteria. *PLoS computational biology* 2010; **6**(7)**:** e1000862.

36. Tavazoie S, Hughes JD, Campbell MJ, Cho RJ, Church GM. Systematic determination of genetic network architecture. *Nat Genet* 1999; **22**(3)**:** 281-285.

37. Beer MA, Tavazoie S. Predicting gene expression from sequence. *Cell* 2004; **117**(2)**:** 185-198.

38. Arnedo J, Svrakic DM, del Val C, Romero,ÄëZaliz R, Hern√°ndez-Cuervo H, Molecular Genetics of Schizophrenia Consortium *et al.* Uncovering the Hidden Risk Architecture of the Schizophrenias: Confirmation in Three Independent Genome-,ÄëWide Association Studies. *The American journal of psychiatry* 2015; **172**(2)**:** 139-153.

39. Kwee LC, Liu D, Lin X, Ghosh D, Epstein MP. A powerful and flexible multilocus association test for quantitative traits. *American journal of human genetics* 2008; **82**(2)**:** 386-397.

40. Ehret GB, Lamparter D, Hoggart CJ, Genetic Investigation of Anthropometric Traits C, Whittaker JC, Beckmann JS *et al.* A multi-SNP locus-association method reveals a substantial fraction of the missing heritability. *American journal of human genetics* 2012; **91**(5)**:** 863-871.

41. Zwir I, Zaliz RR, Ruspini EH. Automated biological sequence description by genetic multiobjective generalized clustering. *Ann N Y Acad Sci* 2002; **980:** 65-82.

42. Cordon O, Herrera F, Zwir I. Linguistic modeling by hierarchical systems of linguistic rules. *Ieee T Fuzzy Syst* 2002; **10**(1)**:** 2-20.

43. Romero-Zaliz R, C. Rubio R, Cord√≥n O, Cobb P, Herrera F, Zwir I. A multi-objective evolutionary conceptual clustering methodology for gene annotation within structural databases: a case of study on the gene ontology database. *IEEE Transactions on Evolutionary Computation*
2008; **12:6:** 679-701.

44. Hinton GE, Salakhutdinov RR. Reducing the dimensionality of data with neural networks. *Science* 2006; **313**(5786)**:** 504-507.

45. Geiger JT, Weininger F, Gemmeke JF, Wollmer M, Schuller B, Rigoll G. Memory-enhanced neural networks and NMF for robust ASR. *IEEE/ACM Transactions on audio, speech, and language processing* 2014; **22**(6)**:** 1037-1046.

46. Le Roux J, Hershey JR, Weininger F. Deep NMF for speech separation. *IEEE Internatiional Conference on Acoustics, Speech, and Signal Processing (ICASSP)*. Mitsubishi Electric Research Laboratories, Inc: Cambridge, Massachusetts, 2015.

47. Cichocki A, Zdunek R, Phan AH, Amari S-i. *Nonnegative Matrix and Tensor Factorizations: Applications to Exploratory Multi-way Data Analysis and Blind Source Separation.* John Wiley & Sons, Inc2009.

48. Mejia-Roa E, Carmona-Saez P, Nogales R, Vicente C, Vazquez M, Yang XY *et al.* bioNMF: a web-based tool for nonnegative matrix factorization in biology. *Nucleic Acids Res* 2008; **36**(Web Server issue)**:** W523-528.

49. Lee DD, Seung HS. Learning the parts of objects by non-negative matrix factorization. *Nature* 1999; **401**(6755)**:** 788-791.

50. Tamayo P, Scanfeld D, Ebert BL, Gillette MA, Roberts CW, Mesirov JP. Metagene projection for cross-platform, cross-species characterization of global transcriptional states. *Proc Natl Acad Sci U S A* 2007; **104**(14)**:** 5959-5964.

51. Bezdek JC. Pattern Analysis. In: Pedrycz W, Bonissone PP, Ruspini EH (eds). *Handbook of Fuzzy Computation*. Institute of Physics Publishing. Oxford University Press: Bristol, 1998, pp F6.1.1-F6.6.20.

52. Bezdek JC, Pal SK, IEEE Neural Networks Council. *Fuzzy models for pattern recognition : methods that search for structures in data*. IEEE Press: New York, 1992, xi, 539pp.

53. Brunet JP, Tamayo P, Golub TR, Mesirov JP. Metagenes and molecular pattern discovery using matrix factorization. *Proc Natl Acad Sci U S A* 2004; **101**(12)**:** 4164-4169.

54. Chagoyen M, Carmona-Saez P, Gil C, Carazo JM, Pascual-Montano A. A literature-based similarity metric for biological processes. *BMC Bioinformatics* 2006; **7:** 363.

55. Chagoyen M, Carmona-Saez P, Shatkay H, Carazo JM, Pascual-Montano A. Discovering semantic features in the literature: a foundation for building functional associations. *BMC Bioinformatics* 2006; **7:** 41.

56. Senbabaoglu Y, Michailidis G, Li JZ. Critical limitations of consensus clustering in class discovery. *Sci Rep* 2014; **4:** 6207.

57. Saeed F, Salim N, Abdo A. Voting-based consensus clustering for combining multiple clusterings of chemical structures. *J Cheminform* 2012; **4**(1)**:** 37.

58. Deb K. *Multi-objective optimization using evolutionary algorithms*. 1st edn. John Wiley & Sons: Chichester ; New York, 2001, xix, 497pp.

59. Deb K. Nonlinear goal programming using multi-objective genetic algorithms. *J Oper Res Soc* 2001; **52**(3)**:** 291-302.

60. Ruspini EH, Zwir I. Automated generation of qualitative representations of complex objects by hybrid soft-computing methods. In: Pal SK, Pal A (eds). *Pattern recognition : from classical to modern approaches*. World Scientific: New Jersey., 2002, pp 454-474.

61. Rissanen J. *Stochastic complexity in statistical inquiry*. World Scientific: Singapore, 1989, 177pp.

62. Romero-Zaliz R, Del Val C, Cobb JP, Zwir I. Onto-CC: a web server for identifying Gene Ontology conceptual clusters. *Nucleic Acids Res* 2008; **36**(Web Server issue)**:** W352-357.

63. Zwir I, Arnedo J, Del-Val C, Pulkki-Råback L, Konte B, Yang SS *et al.* Three gene-environment networks for human personality. *American Journal of Psychiatry* 2018.

64. Goeman JJ, Buhlmann P. Analyzing gene expression data in terms of gene sets: methodological issues. *Bioinformatics* 2007; **23**(8)**:** 980-987.

65. Yu H, Luscombe NM, Lu HX, Zhu X, Xia Y, Han JD *et al.* Annotation transfer between genomes: protein-protein interologs and protein-DNA regulogs. *Genome research* 2004; **14**(6)**:** 1107-1118.

66. A. Mehrle HR, I. Schupp, C. del Val, D. Arlt, F. Hahne, S. Bechtel, J. Simpson, O. Hofman, W. Hide, KH. Glatting, W. Huber, R. Pepperkok, A. Poustka, S. Wiemann. LIFEDB2006. *Nucleic Acid Research* 2006.

67. Previti C, Harari O, Zwir I, del Val C. Profile analysis and prediction of tissue-specific CpG island methylation classes. *BMC Bioinformatics* 2009; **10:** 116.

68. De Smet R, Marchal K. Advantages and limitations of current network inference methods. *Nat Rev Microbiol* 2010; **8**(10)**:** 717-729.

69. Zwir I, Latifi T, Perez JC, Huang H, Groisman EA. The promoter architectural landscape of the Salmonella PhoP regulon. *Mol Microbiol* 2012; **84**(3)**:** 463-485.

70. Zwir I, Yeo WS, Shin D, Latifi T, Huang H, Groisman EA. Bacterial nucleoid-associated protein uncouples transcription levels from transcription timing. *MBio* 2014; **5**(5)**:** e01485-01414.

71. Arnedo J, Romero-Zaliz R, Zwir I, Del Val C. A multiobjective method for robust identification of bacterial small non-coding RNAs. *Bioinformatics* 2014; **30**(20)**:** 2875-2882.

72. Ward LD, Kellis M. HaploReg: a resource for exploring chromatin states, conservation, and regulatory motif alterations within sets of genetically linked variants. *Nucleic Acids Res* 2012; **40**(Database issue)**:** D930-934.

73. Liu K, Yan Z, Li Y, Sun Z. Linc2GO: a human LincRNA function annotation resource based on ceRNA hypothesis. *Bioinformatics* 2013; **29**(17)**:** 2221-2222.

74. Chen X, Hao Y, Cui Y, Fan Z, He S, Luo J *et al.* LncVar: a database of genetic variation associated with long non-coding genes. *Bioinformatics* 2016.

75. Glazar P, Papavasileiou P, Rajewsky N. circBase: a database for circular RNAs. *RNA* 2014; **20**(11)**:** 1666-1670.

76. Li JH, Liu S, Zhou H, Qu LH, Yang JH. starBase v2.0: decoding miRNA-ceRNA, miRNA-ncRNA and protein-RNA interaction networks from large-scale CLIP-Seq data. *Nucleic Acids Res* 2014; **42**(Database issue)**:** D92-97.

77. Huang da W, Sherman BT, Lempicki RA. Systematic and integrative analysis of large gene lists using DAVID bioinformatics resources. *Nat Protoc* 2009; **4**(1)**:** 44-57.

78. Du J, Yuan Z, Ma Z, Song J, Xie X, Chen Y. KEGG-PATH: Kyoto encyclopedia of genes and genomes-based pathway analysis using a path analysis model. *Mol Biosyst* 2014; **10**(9)**:** 2441-2447.

79. Karp PD, Billington R, Holland TA, Kothari A, Krummenacker M, Weaver D *et al.* Computational Metabolomics Operations at BioCyc.org. *Metabolites* 2015; **5**(2)**:** 291-310.

80. Kupershmidt I, Su QJ, Grewal A, Sundaresh S, Halperin I, Flynn J *et al.* Ontology-based meta-analysis of global collections of high-throughput public data. *PLoS One* 2010; **5**(9).

81. Jaffe AE, Storey JD, Ji H, Leek JT. Gene set bagging for estimating the probability a statistically significant result will replicate. *BMC Bioinformatics* 2013; **14:** 360.

82. Lu Q, Yao X, Hu Y, Zhao H. GenoWAP: GWAS signal prioritization through integrated analysis of genomic functional annotation. *Bioinformatics* 2016; **32**(4)**:** 542-548.

83. Stone JL, Merriman B, Cantor RM, Geschwind DH, Nelson SF. High density SNP association study of a major autism linkage region on chromosome 17. *Hum Mol Genet* 2007; **16**(6)**:** 704-715.

84. Arnedo J, Mamah D, Baranger DA, Harms MP, Barch DM, Svrakic DM *et al.* Decomposition of brain diffusion imaging data uncovers latent schizophrenias with distinct patterns of white matter anisotropy. *NeuroImage* 2015; **120:** 43-54.

85. Zwir I, Mishra P, Del Val C, Gu CC, de Erausquin GA, Lehtimäki T *et al.* Uncovering the Complex Genetics of Human Personality: Response from authors on the PGMRA. *Mol Psychiatry* 2019.

86. Zwir I, Arnedo J, Del-Val C, Pulkki-Raback L, Konte B, Yang SS *et al.* Uncovering the complex genetics of human character. *Mol Psychiatry* 2018.

87. Zwir I, Arnedo J, Del-Val C, Pulkki-Raback L, Konte B, Yang SS *et al.* Uncovering the complex genetics of human temperament. *Mol Psychiatry* 2018.

88. Arnedo J, del Val C, de Erausquin GA, Romero-Zaliz R, Svrakic D, Cloninger CR *et al.* PGMRA: A web server for (Phenotype X Genotype) many-to-many relation analysis in GWAS. *Nucleic acids research* 2013; (Web Server issue).

89. Derringer J. Explaining heritable variance in human character. *bioRxiv* 2018**:** 446518.

90. Wu MC, Kraft P, Epstein MP, Taylor DM, Chanock SJ, Hunter DJ *et al.* Powerful SNP-set analysis for case-control genome-wide association studies. *American journal of human genetics*; **86**(6)**:** 929-942.

91. Houle D, Govindaraju DR, Omholt S. Phenomics: the next challenge. *Nature reviews* 2011; **11**(12)**:** 855-866.

92. International Schizophrenia C, Purcell SM, Wray NR, Stone JL, Visscher PM, O'Donovan MC *et al.* Common polygenic variation contributes to risk of schizophrenia and bipolar disorder. *Nature* 2009; **460**(7256)**:** 748-752.

93. Lango Allen H, Estrada K, Lettre G, Berndt SI, Weedon MN, Rivadeneira F *et al.* Hundreds of variants clustered in genomic loci and biological pathways affect human height. *Nature* 2010; **467**(7317)**:** 832-838.

94. Russell SJ, Norvig P. *Artificial intelligence : a modern approach*. 3rd edn. Prentice Hall: Upper Saddle River, N.J., 2010, xviii, 1,132 p.pp.

95. Dudbridge F. Power and predictive accuracy of polygenic risk scores. *PLoS Genet* 2013; **9**(3)**:** e1003348.

96. Brunton SL, Proctor JL, Kutz JN. Discovering governing equations from data by sparse identification of nonlinear dynamical systems. *Proc Natl Acad Sci U S A* 2016; **113**(15)**:** 3932-3937.

97. Deb K. *Multi-objective optimization using evolutionary algorithms*. 1st edn. John Wiley & Sons: Chichester ; New York, 2001, xix, 497 p.pp.

98. Machiela MJ, Chen CY, Chen C, Chanock SJ, Hunter DJ, Kraft P. Evaluation of polygenic risk scores for predicting breast and prostate cancer risk. *Genet Epidemiol* 2011; **35**(6)**:** 506-514.

99. Feldman MW, Ramachandran S. Missing compared to what? Revisiting heritability, genes and culture. *Philos Trans R Soc Lond B Biol Sci* 2018; **373**(1743).

100. Krapohl E, Patel H, Newhouse S, Curtis CJ, von Stumm S, Dale PS *et al.* Multi-polygenic score approach to trait prediction. *Mol Psychiatry* 2018; **23**(5)**:** 1368-1374.

101. Selzam S, Krapohl E, von Stumm S, O'Reilly PF, Rimfeld K, Kovas Y *et al.* Predicting educational achievement from DNA. *Mol Psychiatry* 2017; **22**(2)**:** 267-272.

102. Torkamani A, Wineinger NE, Topol EJ. The personal and clinical utility of polygenic risk scores. *Nature reviews* 2018; **19**(9)**:** 581-590.

103. Boyle EA, Li YI, Pritchard JK. An Expanded View of Complex Traits: From Polygenic to Omnigenic. *Cell* 2017; **169**(7)**:** 1177-1186.

104. Võsa U, Claringbould A, Westra H-J, Bonder MJ, Deelen P, Zeng B *et al.* Unraveling the polygenic architecture of complex traits using blood eQTL meta-analysis. *bioRxiv* 2018**:** 447367.

105. Boyle EA, Li YI, Pritchard JK. The Omnigenic Model: Response from the Authors. *J Psychiatry Brain Sci* 2017; **2**(5)**:** s8.

106. Lea A, Subramaniam M, Ko A, Lehtimäki T, Raitoharju E, Kähönen M *et al.* Genetic and environmental perturbations lead to regulatory decoherence. *bioRxiv* 2018**:** 369306.

107. Wray NR, Yang J, Hayes BJ, Price AL, Goddard ME, Visscher PM. Pitfalls of predicting complex traits from SNPs. *Nature reviews* 2013; **14**(7)**:** 507-515.

108. Kolesnikov N, Hastings E, Keays M, Melnichuk O, Tang YA, Williams E *et al.* ArrayExpress update--simplifying data submissions. *Nucleic Acids Res* 2015; **43**(Database issue)**:** D1113-1116.

C. Supplemental Tables

Table S1. Descriptors for high and low scorers on TCI subscales.

Table S2. Distinguishing features of three subtypes or clusters of temperament (adapted from Thomas and Chess, 1977 [109] and Cloninger et al, The Complex Genetics and Biology of Human Temperament, A review of traditional concepts in relation to modern molecular findings. *Molecular Psychiatry* 2019)

Table S3. (A) Comparison of physical, emotional, social, cognitive indicators of health of people in 3 personality networks in Young Finns Study (n = 2126). (B) Calculation of the each subject's level on previously validated indicators of ill-being and well-being. Ill-being is indicated by low scores on Self-directness and Cooperativeness (SD + CO). Well-being is the product SD x CO x ST (Self-transcendence). The Boolean risk of ill-being is the bottom decile of its indicator for 1 and 0 otherwise. The Boolean classification of well-being is the top decile of its indicator for 1 and 0 otherwise. Semi-continuous ratings from 1 to 6 are also given by separating subjects into 6 equal classes based on their normalized (percentile) rank from 0 to 1. Longitudinal physical, emotional, and social indices are described. Higher scores means better health.

Table S4. Character and Temperament Sets comprised of TCI measurements at the subscale level were identified by PGMRA and associated as Relationships (see Tables S4 and S5). Supersets consist of low granularity groups of Character and Temperament Sets with a linguistic profile. The health risk of these associations is shown (well or ill extremes in the unit scale, see Supplementary Table S2B).

Table S5. Comparison of the size of the 3 networks in terms of number of subjects and other components.

Table S6. SNP sets mapped into the three phenotypic networks. SNP sets are described by their labels and associated Temperament and Character profiles.

Table S7. Genotypic-phenotypic Relationships among Temperament, Character, and SNP Sets identified by PGMRA. SNP Sets (p-value <1E-05) associated with Temperament

and Character phenotypes, their significance compared to that of the averaged, best, and worst values of the individual SNPs, their size (#Subjects, #SNPs), and corresponding health risk (well or ill extremes in the unit scale, see Supplementary Table S2B) are shown. (Many-to-many optimal relationships were identified using Multi-objective optimization techniques, see Methods.)

Table S8. Percentage of overlapping among SNP sets related to Temperament and Character sets, respectively, across networks.

Table S9. 972 genes belonging to different networks. *indicates genes mapped by a large SNP set G_3_1, and #indicates genes that are recognized by just one SNP set.

Table S10. Description of the different types and sub-types of genes based on their possible molecular consequences.

Table S11. Definition of environmental variables.

Table S12. Summary of the associations among Environmental sets and the phenotypic Temperament and Character relationships. Environmental sets related directly to the phenotypic associations between Temperament and Character sets are summarized and compared among all relationships, as well as within each network. Environmental sets indirectly associated with Temperament and Character relationships through SNP sets are also described. "Not related" indicates that there is no Environmental set related to any component of the relationship: Temp set - Char set or the SNP sets associated with a

Temp set and a Char set. "Related" indicates that both Char and Temp sets, or their related SNP sets in the indirect fashion, are associated with an Environmental set.

Table S13: Estimation of variance explained (R^2) by genotype alone (regression N_1), environment alone (regression N_2), and both jointly (regression N_3) for 3 measures of health status (ill-being, well-being, and overall health)

Table S14: Estimated coefficients for the model corresponding to regression N3 (full model), with number of observations: 1804; error degrees of freedom: 1787; Root Mean Squared Error: 0.253; R^2-squared: 0.90; Adjusted R^2 0.896; F-statistic vs. constant model: 972; p-value < 1E-130 (red highlighted values indicate variables discarded by the Stepwise procedure, Matlab R2017b)

Table S15. Relationships between Temperament and Character Sets identified in the Finnish sample and replicated in the Korean (80%) sample. The replication score was calculated using Hypergeometric statistics and Multi-objective optimization techniques (see Pareto values in Table S17).

Table S16. Relationships between Temperament and Character Sets identified in the Finnish sample and replicated in the German (64%) sample. The replication score was calculated using Hypergeometric statistics and Multi-objective optimization techniques (see Pareto values in Table S18).

D. Supplemental Figures

Figure S1. Schematic of the PGMRA method applied to multiple domains of knowledge (see Supplementary Method). (A) Deep learning network developed by PGMRA fusing different domain of knowledge in a semi-supervised fashion including: unsupervised autoencoding, multiobjective optimization and pooling, interpretable association of types of knowledge, labeling the associations, and developing a classifier. Each layer has its own learning process and constitutes the input of the next layer. (B) Schematic of the genotypic-phenotypic associations identified by PGMRA in an unsupervised fashion. (C) Flow chart of the PGMRA process. (D) PGMRA performs a deep unsupervised NMF learning process: (i) NMF is implemented based on decomposing an input dataset, encoded as a matrix (or a tensor) composed of features and observations/subjects, into smaller factors. The learning process is a mirror process because it consists of comparing the original matrix with that reconstructed from the factors, and adjust those factors by the error. Factors are derived by combining matrix W and H. (iii) NMF can be transformed into a supervised method by moving matrix W to the other side of the equation applying the pseudoinverse of a product. (iii) Illustration of the process carried out by the NMF method to learn one factor: ordering the columns of W, as well as the rows of H, and multiply them. (E) Deep NMF process systematically applied (convolutive) using different number of maximum clusters or granularity levels (Consensus clustering). Optimal submatrices (factors) are selected from all levels by a multiobjective optimization process. This image can also illustrate the recurrent application of NMF to identify high level profiles. (F) Schematic that exemplifies how PGMRA sees the biomedical datasets (GWAS, DTI images, etc.). 6 patients have a deficit (value = 20) in different regions. Typically, the average of each cell is calculated, and, as a consequence, there is no region with a particular deficit (all values = 87) in all patients.

Because averaging the cells conceals the differences among patients, PGMRA is focused on segmentation of patients and features.

Figure S2. Composition of the phenotypic networks. (A) The Creative-Reliable is primarily composed of reliable (violet) and creative SNP sets, but also by sensitive (black) and organized SNP sets. (B) The Organized-Reliable is primarily composed of reliable (violet) and organized SNP sets, but also displays a small proportion of associations that include sensitive (black) and antisocial (orange) SNP sets. (C) The Emotional-Unreliable network is primarily composed of dependent and apathetic SNP sets associated with sensitive (black) and antisocial (orange) SNP sets. (D) The three dimensional view of the Temperament and Character profiles that compose the phenotypic and genotypic networks.

Figure S3. Evaluation of probability of health in Temperament and Character Sets and their relationships with SNP Sets using ANOVA statistics. (A) Health risk evaluated for Genotypic-phenotypic Relationships between Temperament and Character Sets and Supersets associated with SNP Sets (compare with Figure 3B). (B) Ill risk evaluated for Genotypic-phenotypic Relationships between Temperament and Character Sets and Supersets associated with SNP Sets (compare with Figure 3E). Adding genotypic information allows a better discrimination among Phenotypic Supersets (see (A) and (B)).

Figure S4. (A) Histogram representing the chromosomal location of genes corresponding to SNP Sets associated with Temperament and Character Relationships. The bars revealed differences between genes related only to Temperament Sets (red color), Character Sets (blue color), and Temperament and Character Relationships (green color).

(B) Sub-types of genetic variants mapped by SNP sets associated with character: Specific molecular consequences [Genes related only to character sets (red) were less often protein coding and more often RNA genes than those also associated with temperament sets (blue color), or genes related to both character and temperament exhibit higher proportion of protein coding genes]

Figure S5. Relationships among key genes associated with Temperament and Character Sets that constitute AND/OR relationships that discriminate the three networks shown in Figure 1: self-awareness (violet), self-control (blue) and emotional-reactivity (orange).

Figure S6. Relationships among environmental sets associated with Temperament and Character Sets subnetworks (Figure 1). Environmental sets can belong to one or more networks.

Figure S7. Surfaces representing the health function of the uncovered Genotypic-phenotypic Relationships between SNP Sets and Temperament and Character associations. The probability of health (z-axis; red high; green: low) was calculated based on the distribution of the status of subjects within each relationship, and the surface was plotted interpolating the relation domains. The order adopted for plotting SNP Sets is calculated based on clustering shared subjects in SNP Sets (x-axis) and in Temperament and Character Relationships (y-axis) using Hypergeometric statistics (see Method). (Close-located SNP or Temperament and Character associations in an edge share more subjects than those located far away.) (A) Well-being surface. (B) Ill-being surface.

Figure S8. The German sample was screened to exclude psychopathology, including personality disorders, so it had a reduced number of individuals with unhealthy personalities, as it is seen by the distribution/histogram of well-being in the Finnish and German Samples. First, we estimated the 3rd quartile in the Finnish normal distribution that suggests a value of 0.71 (SD+CO/Max in the unit interval). Second, we calculated the proportion of the Finnish that are equal or higher than this value: 24%. Third, we calculated the number of Germans that are above that reference value: 89%. Fourth, we calculated the Wilcoxon rank test from these two populations >0.71 and the p-value is 1.13E-81.

Figure S9. The expression of genes associated with temperament and character in multiple organ systems. Expression in particular organs was systematically identified using ArrayExpress [110].

Figure S10. Flow chart describing the common, as well as the different roads followed by methods developed to build polygenic scores and the PGMRA method.
