# Supplementary figures and images for: Three genetic–environmental networks for human personality
Source: Mol Psychiatry. 2019 Nov 21;26(8):3858–75. doi: 10.1038/s41380-019-0579-x (PMC8550959; doi:10.1038/s41380-019-0579-x)

A

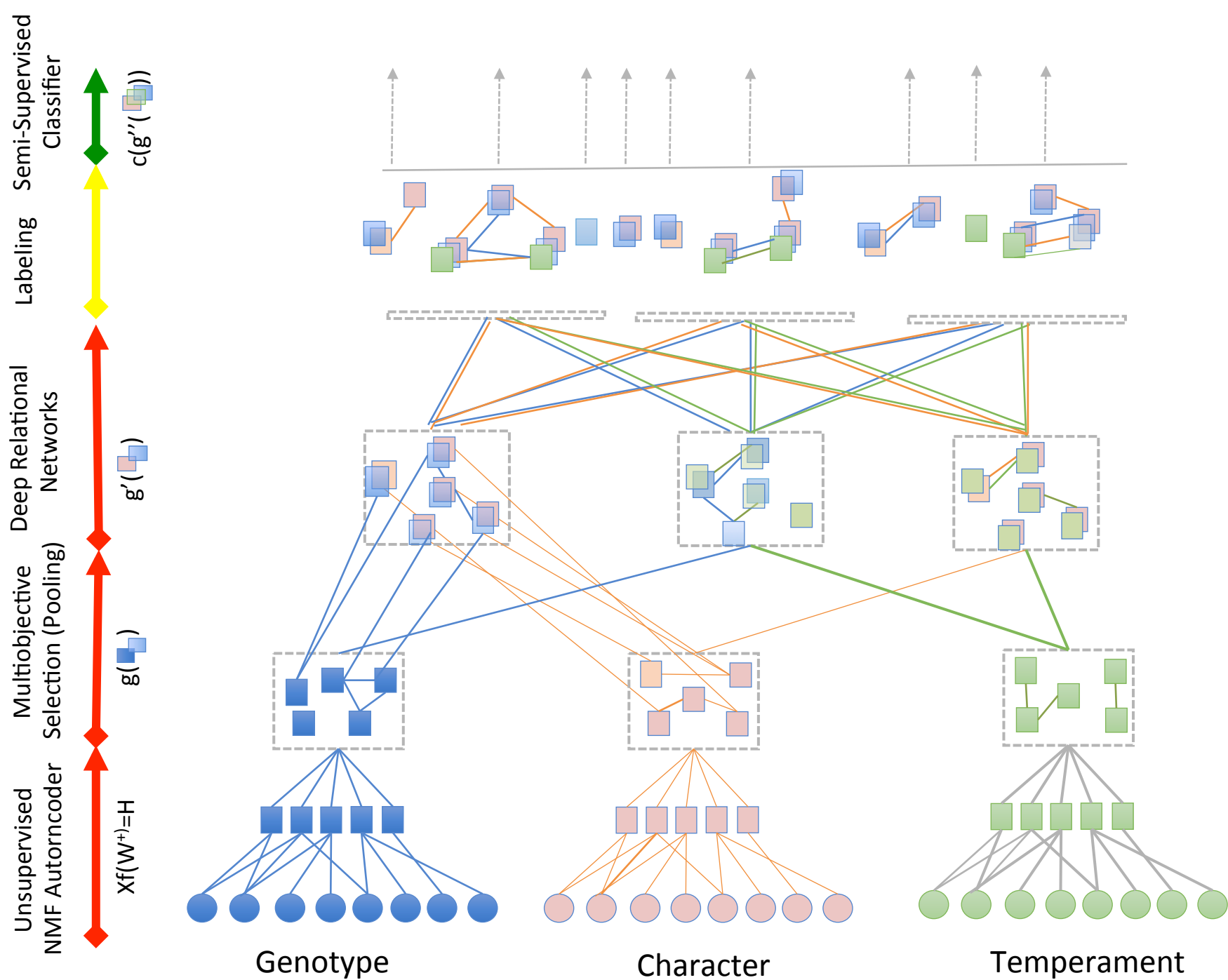

B

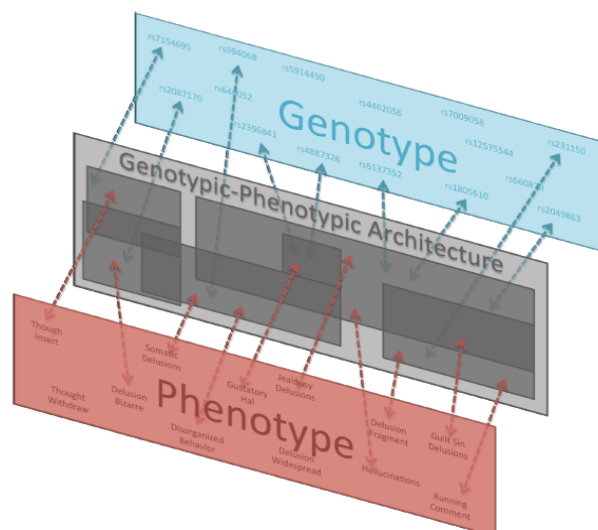

C

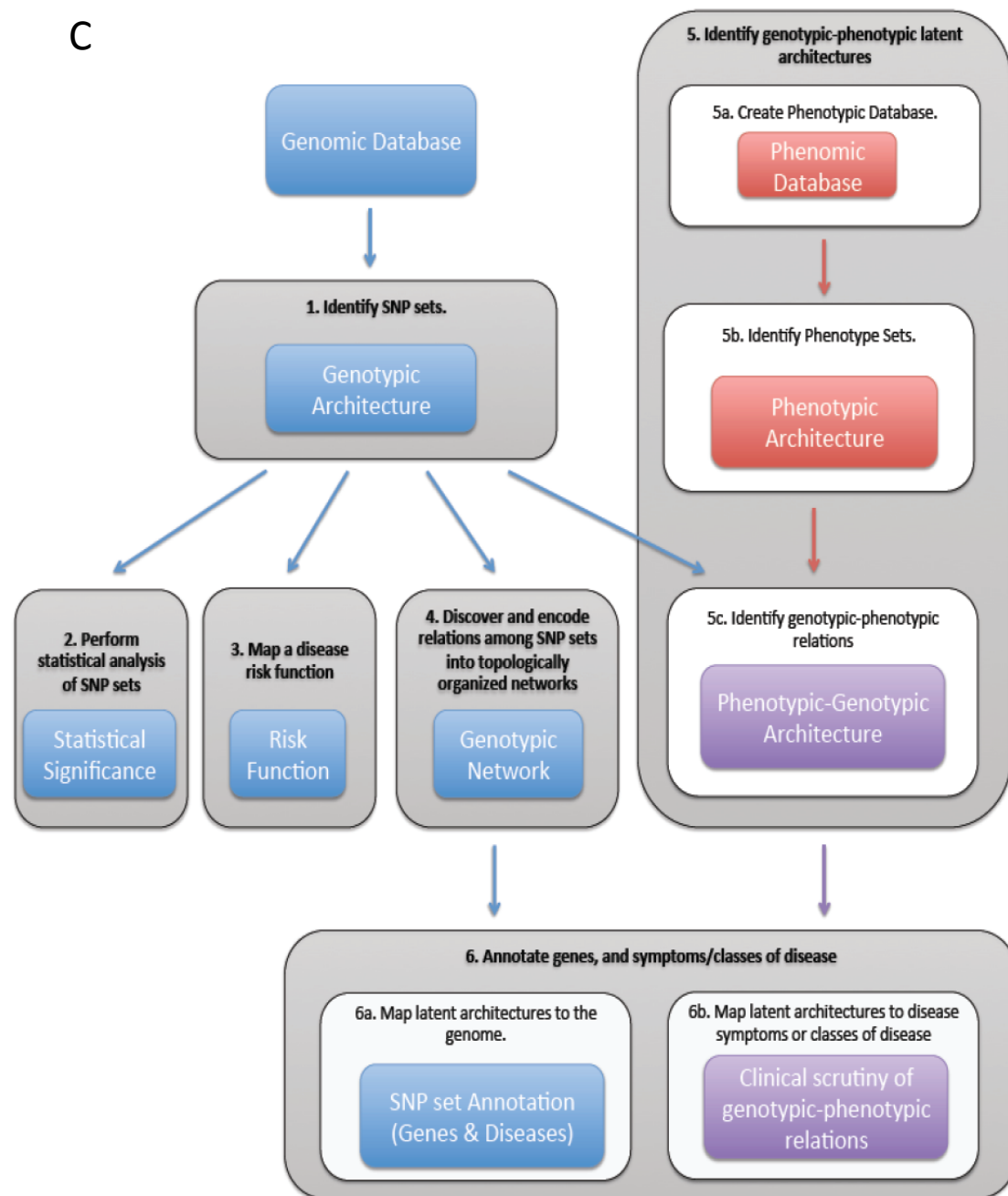

D

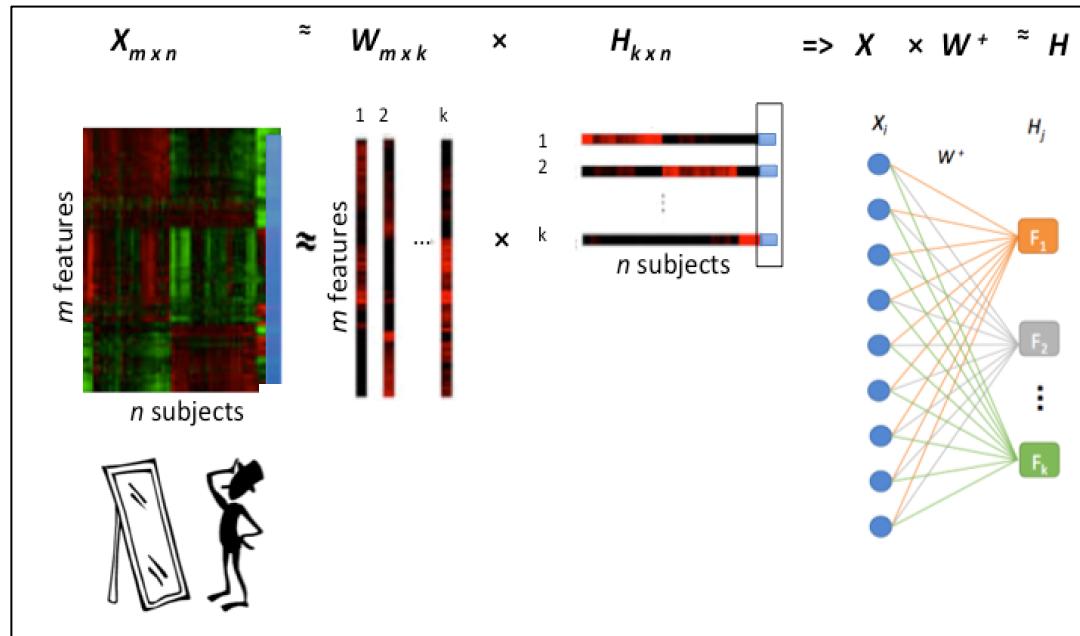

F

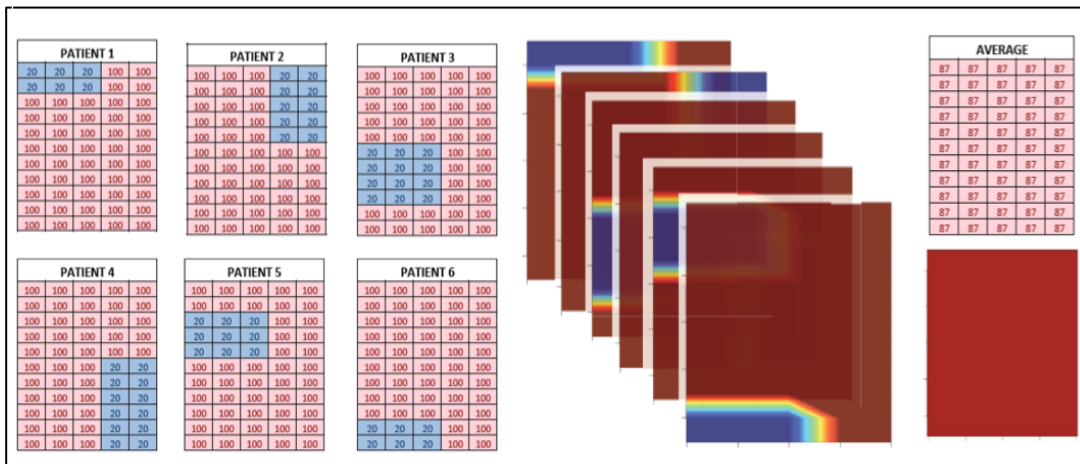

E

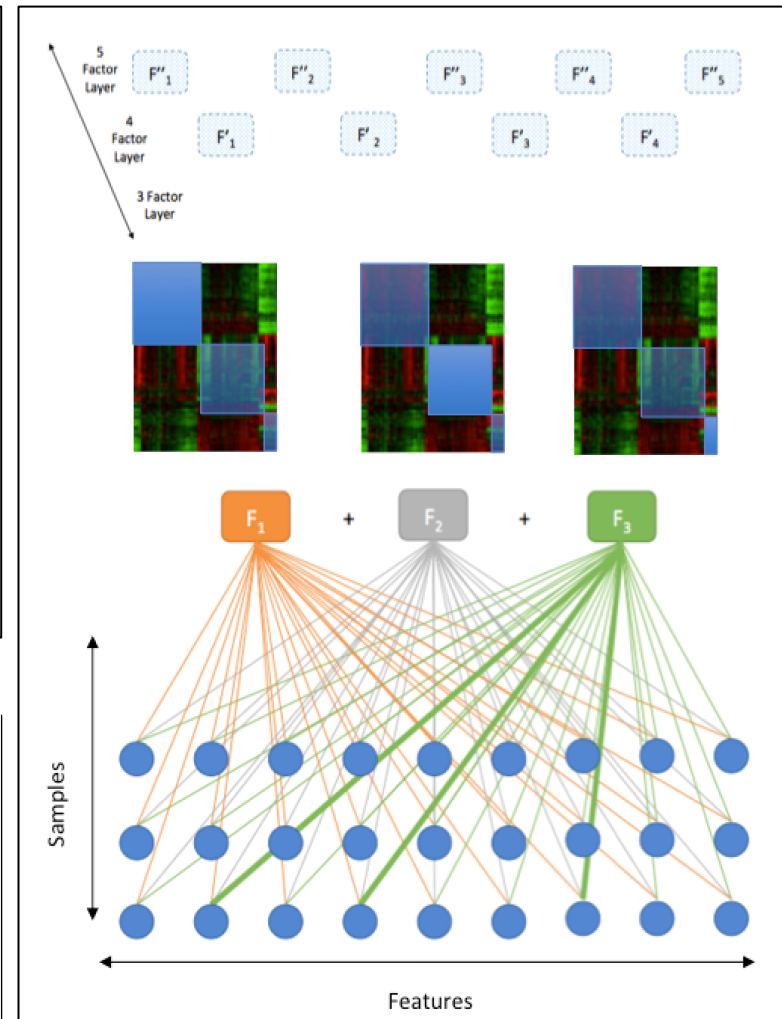

Figure S1

Supplement: Supplementary file 2 — Supplementary Figure S1 [file 41380_2019_579_MOESM2_ESM.pdf]

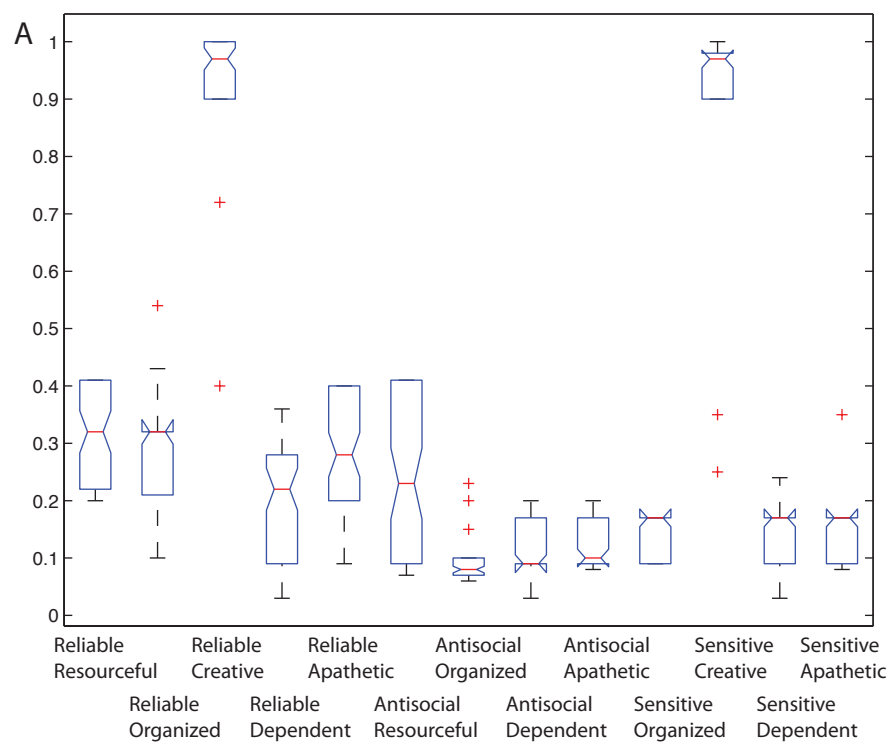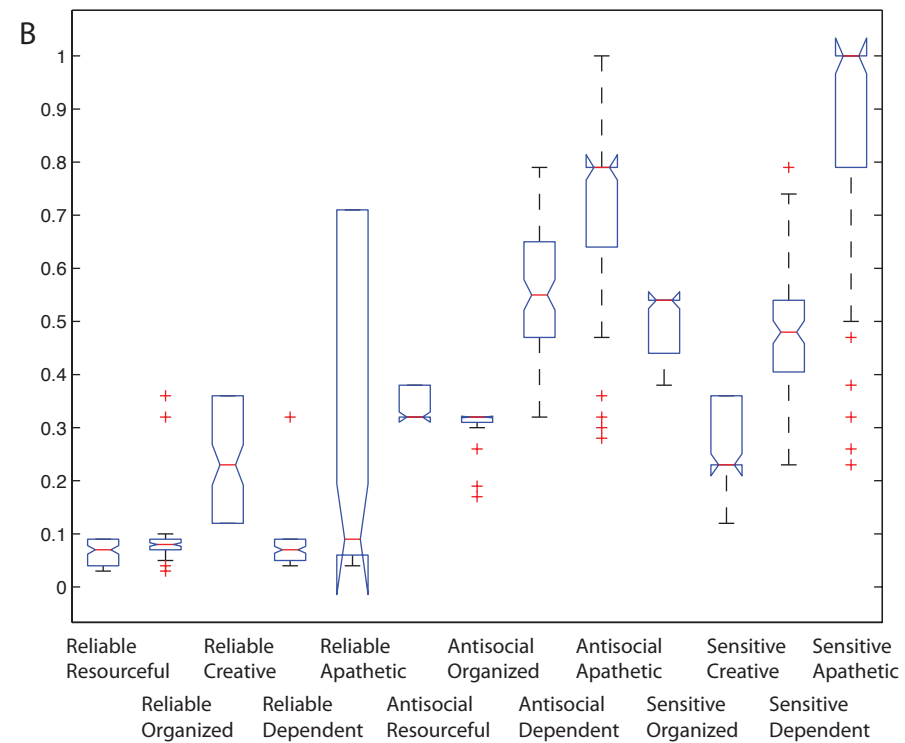

Figure S3

Supplement: Supplementary file 4 — Supplementary Figure S3 [file 41380_2019_579_MOESM4_ESM.pdf]

A

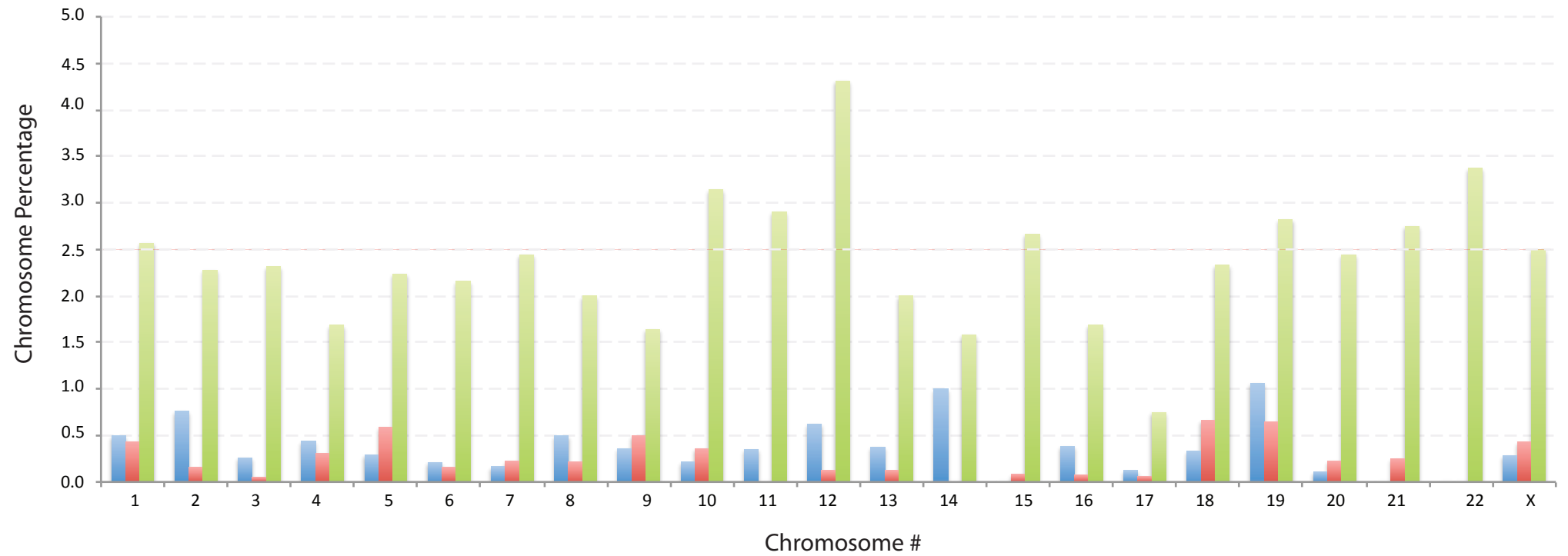

B

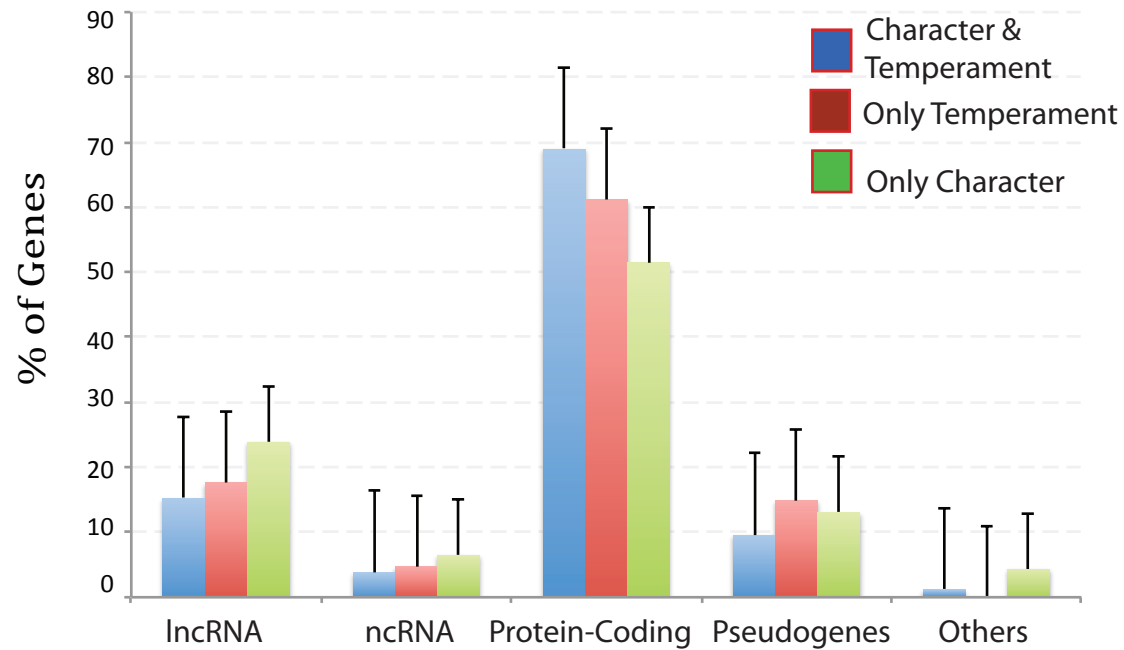

Figure S4

Supplement: Supplementary file 5 — Supplementary Figure S4 [file 41380_2019_579_MOESM5_ESM.pdf]

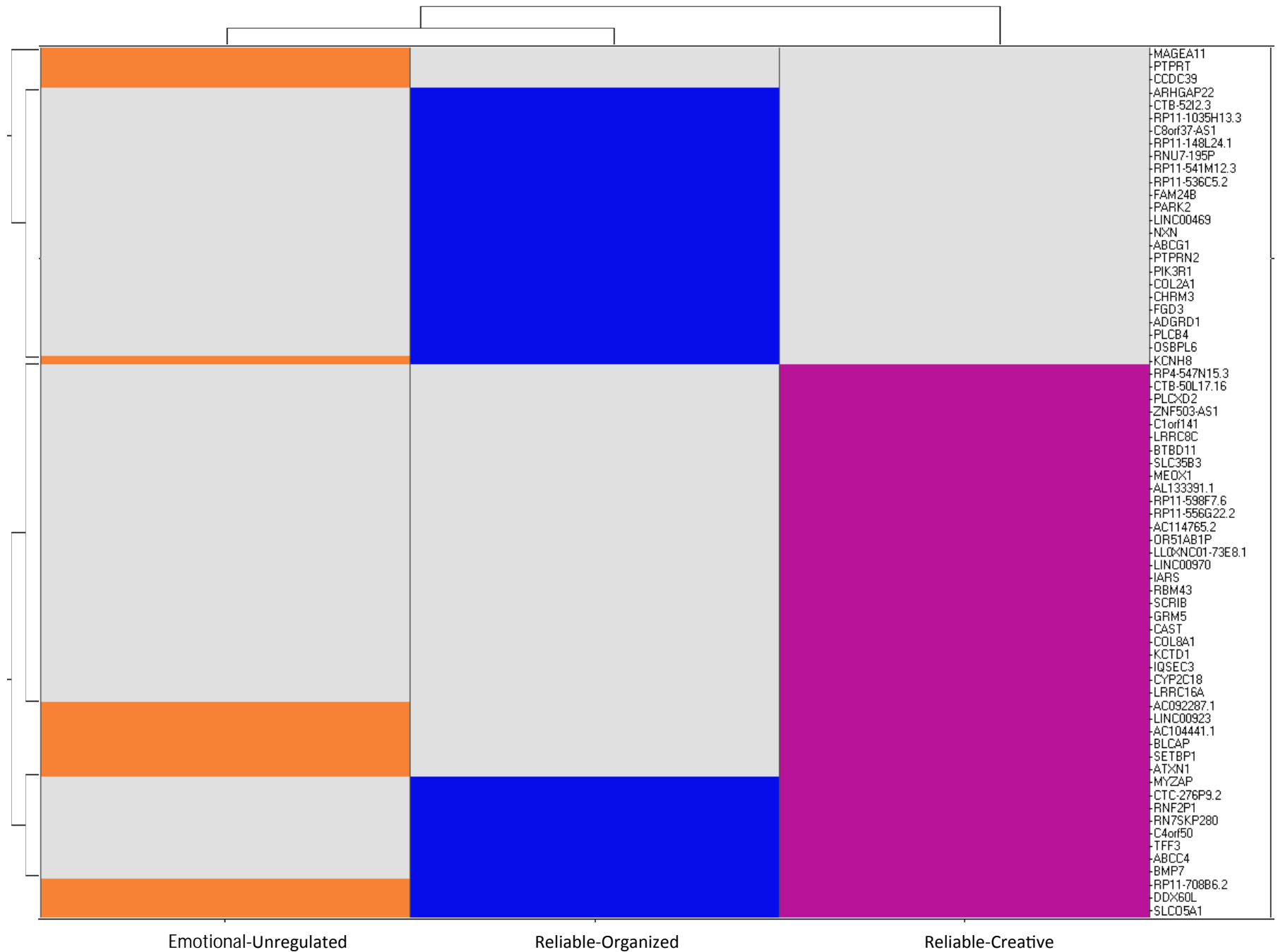

Figure S5

Supplement: Supplementary file 6 — Supplementary Figure S5 [file 41380_2019_579_MOESM6_ESM.pdf]

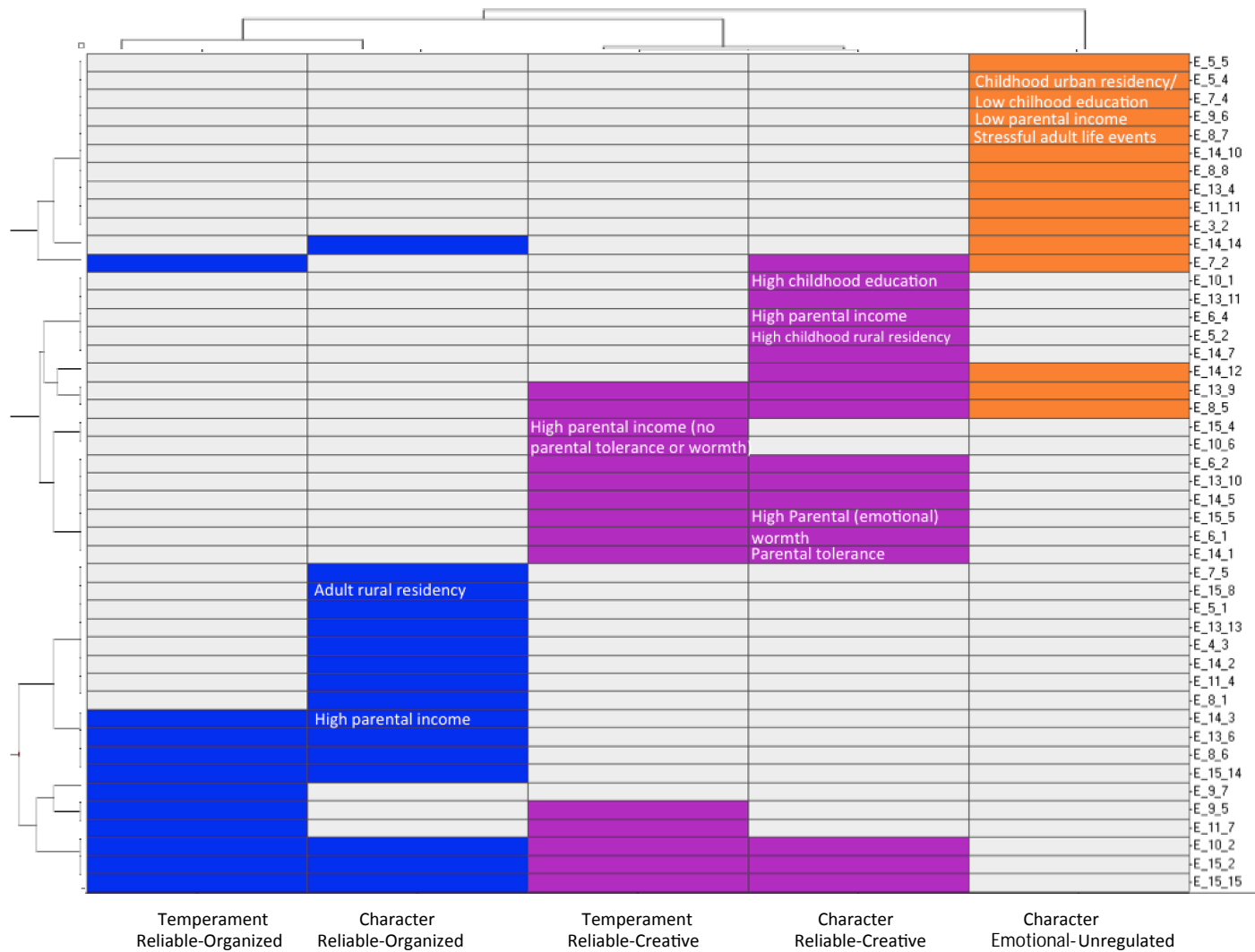

Figure S6

Supplement: Supplementary file 7 — Supplementary Figure S6 [file 41380_2019_579_MOESM7_ESM.pdf]

A

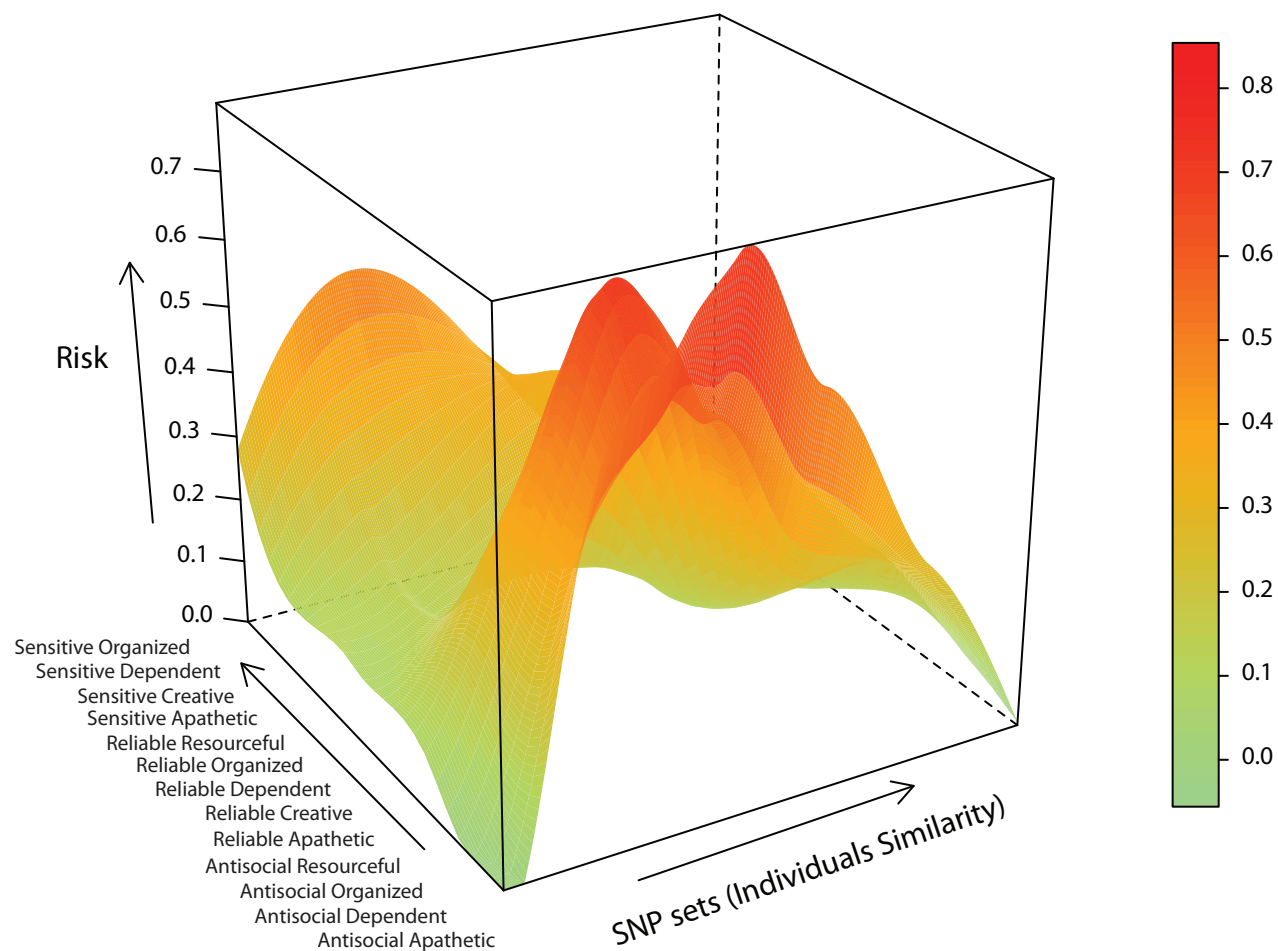

B

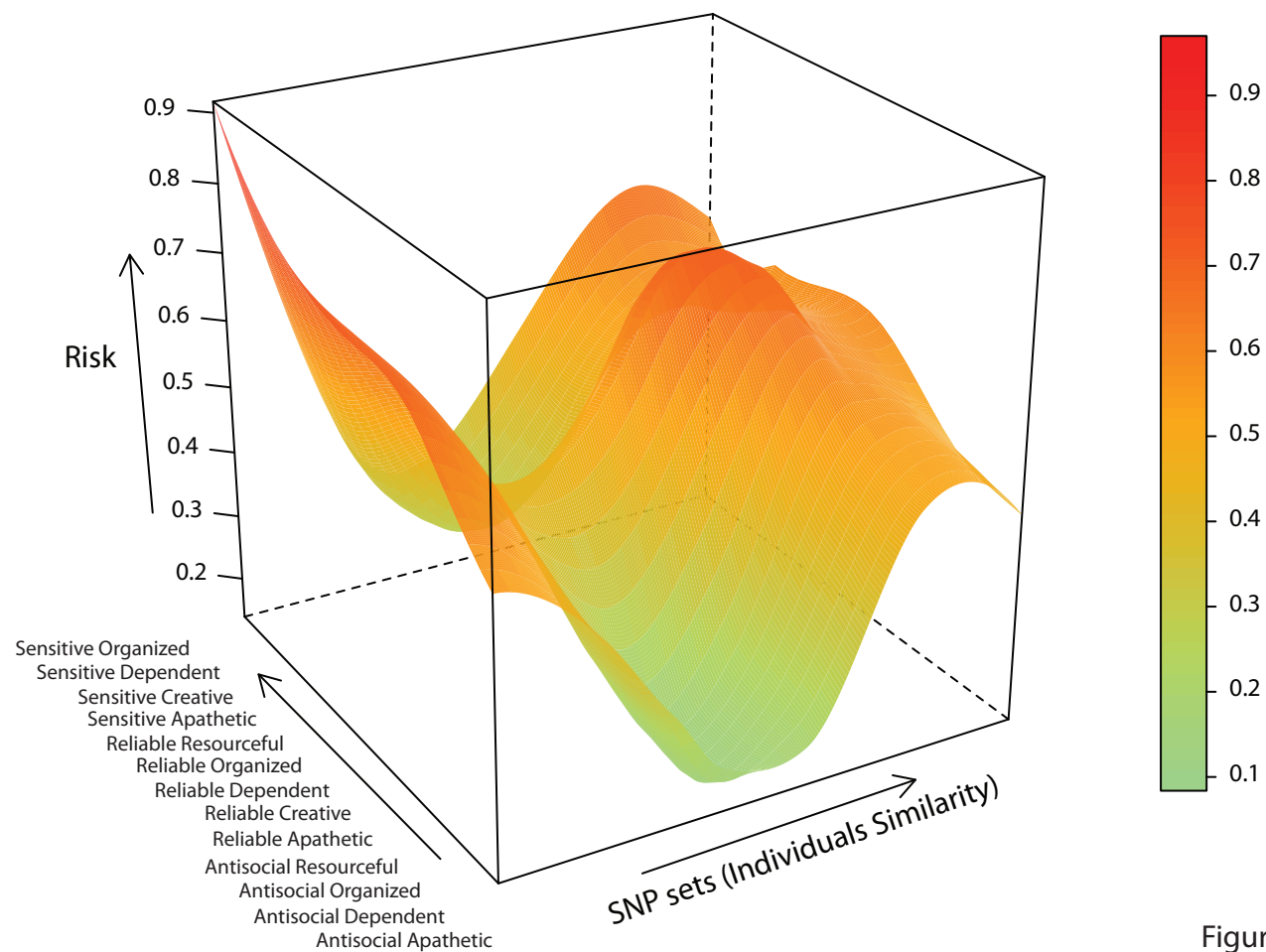

Figure S7

Supplement: Supplementary file 8 — Supplementary Figure S7 [file 41380_2019_579_MOESM8_ESM.pdf]

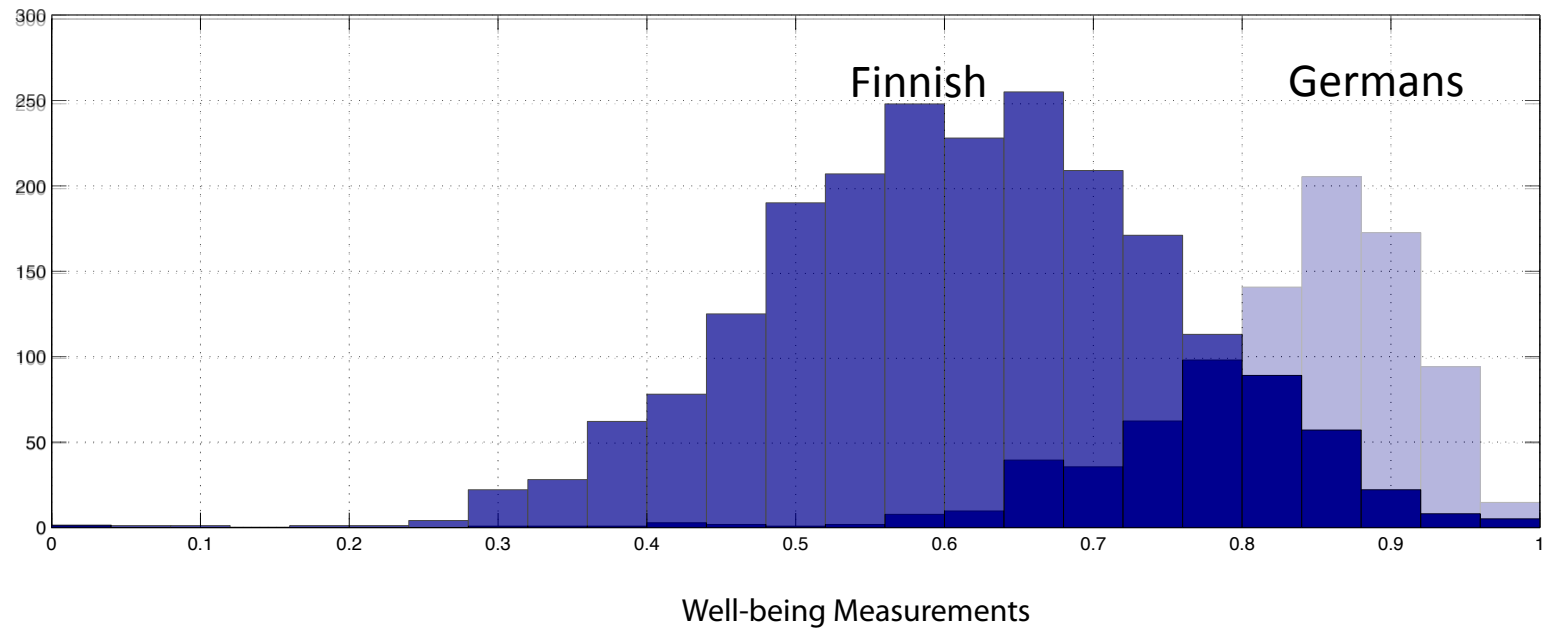

Figure S8

Supplement: Supplementary file 9 — Supplementary Figure S8 [file 41380_2019_579_MOESM9_ESM.pdf]

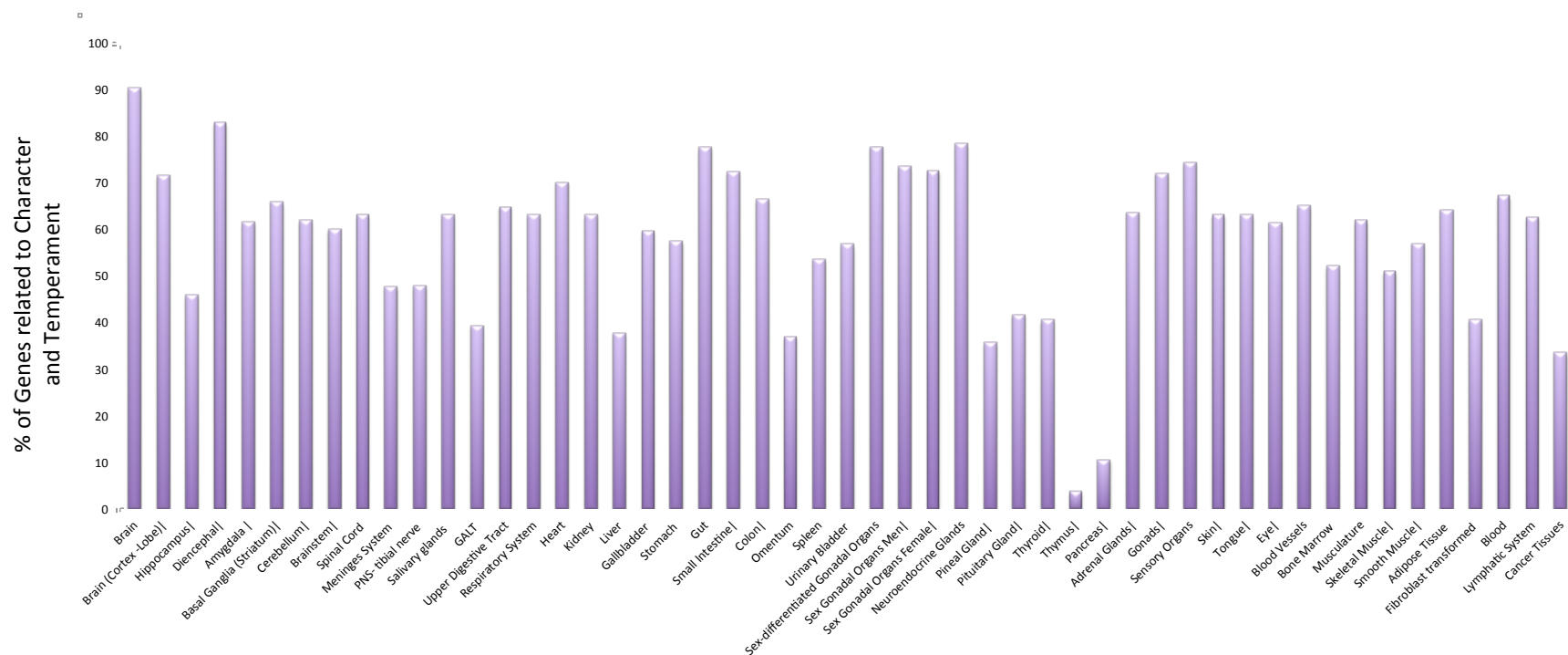

Organ/Tissue ( | indicates a sub-category)

Figure S9

Supplement: Supplementary file 10 — Supplementary Figure S9 [file 41380_2019_579_MOESM10_ESM.pdf]

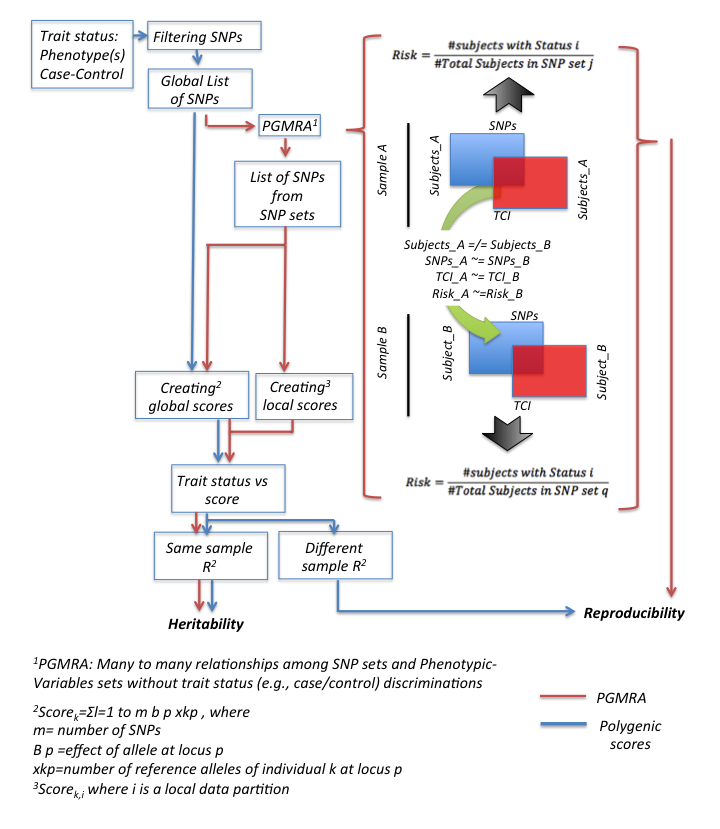

Supplement: Supplementary file 11 — Supplementary Figure S10 [file 41380_2019_579_MOESM11_ESM.tif]
