# Supplementary material for: Three genetic–environmental networks for human personality
Source: Mol Psychiatry. 2019 Nov 21;26(8):3858–75. doi: 10.1038/s41380-019-0579-x (PMC8550959; doi:10.1038/s41380-019-0579-x)
Supplement: Supplementary file 3 — Supplementary Figure S2 [file 41380_2019_579_MOESM3_ESM.pdf]

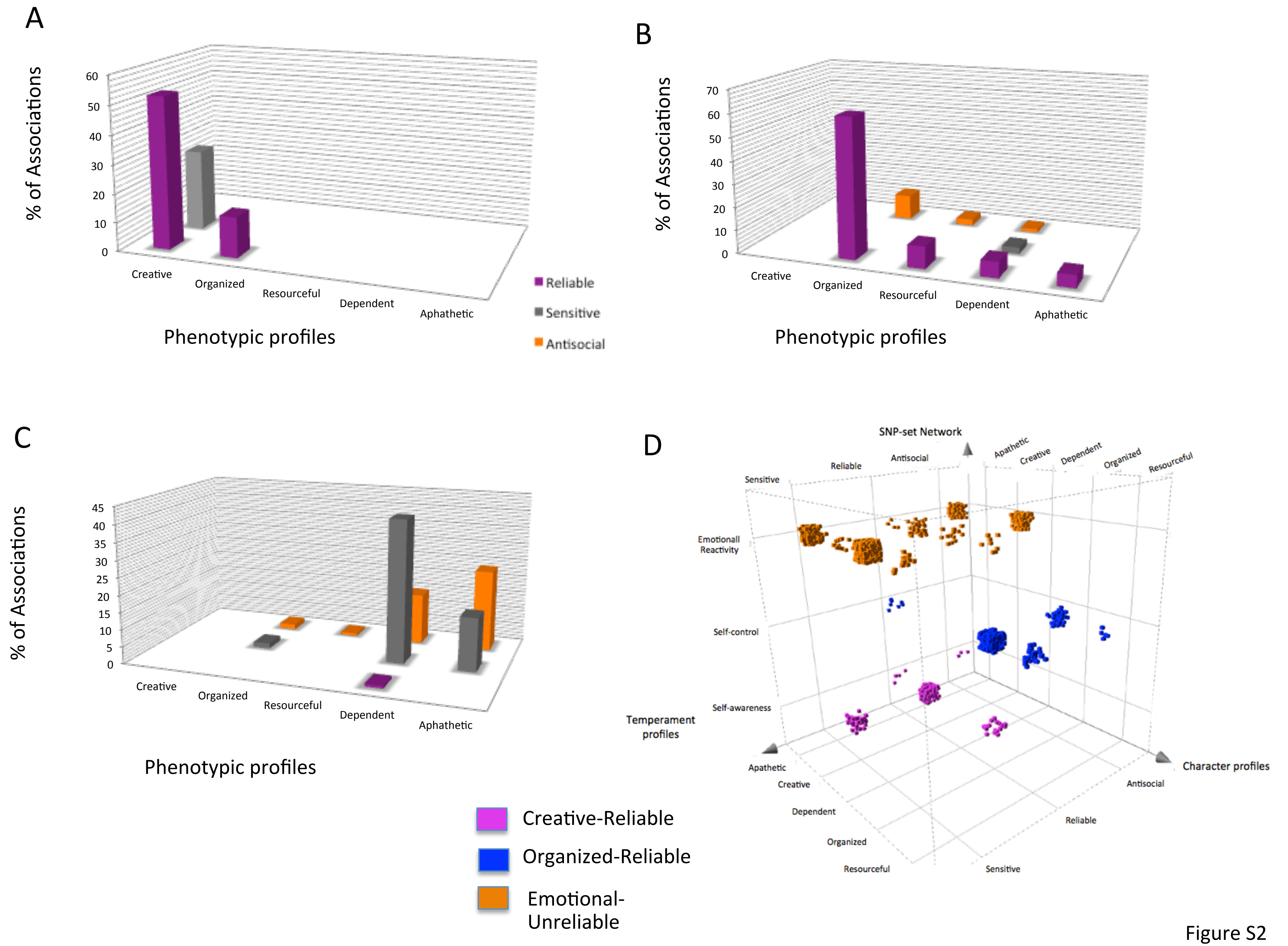

A
% of Associations
60
50
40
30
20
10
0
Creative
Organized
Resourceful
Dependent
Aphathetic
Phenotypic profiles
Reliable
Sensitive
Antisocial
B
% of Associations
70
60
50
40
30
20
10
0
Creative
Organized
Resourceful
Dependent
Aphathetic
Phenotypic profiles
C
% of Associations
45
40
35
30
25
20
15
10
5
0
Creative
Organized
Resourceful
Dependent
Aphathetic
Phenotypic profiles
Creative-Reliable
Organized-Reliable
Emotional-Unreliable
D
SNP-set Network
Sensitive
Reliable
Antisocial
Apathetic
Creative
Dependent
Organized
Resourceful
Emotionall Reactivity
Self-control
Self-awareness
Temperament profiles
Apathetic
Creative
Dependent
Organized
Resourceful
Sensitive
Reliable
Antisocial
Character profiles

Figure S2
