# Supplementary material for: Three genetic–environmental networks for human personality
Source: Mol Psychiatry. 2019 Nov 21;26(8):3858–75. doi: 10.1038/s41380-019-0579-x (PMC8550959; doi:10.1038/s41380-019-0579-x)
Supplement: Supplementary file 13 — Supplementary Table S2 [file 41380_2019_579_MOESM13_ESM.docx]

**Supplementary Table S2**. Distinguishing features of three subtypes or clusters of temperament (adapted from Thomas and Chess, 1977 (6) and Cloninger et al, The Complex Genetics and Biology of Human Temperament, A review of traditional concepts in relation to modern molecular findings. 2019, in review)

| Dimensions of Temperament | **Easy**  **Subtype** | **Difficult Subtype** | **Slow-to-Warm-Up**  **Subtype** |
| --- | --- | --- | --- |
| **Thomas & Chess** |  |  |  |
| Activity Level | variable | variably high | low to moderate |
| Rhythmicity  (sleep, eating,  toileting) | highly regular | irregular | variable |
| Adaptability to  changes in  routine | rapid | slow | slow |
| Approachability | positive approach | withdrawal | initial withdrawal |
| Intensity of  emotional  arousal | low or mild | intense | mild |
| Valence of  Mood | positive | negative | indifferent or slightly negative |
| **Cloninger TCI** |  |  |  |
| Harm Avoidance | Low | High | variable |
| Novelty Seeking | Low | High | High |
| Reward  Dependence | High | High | Low |
| Persistence | High | variable | Low |
