# Supplementary material for: Three genetic–environmental networks for human personality
Source: Mol Psychiatry. 2019 Nov 21;26(8):3858–75. doi: 10.1038/s41380-019-0579-x (PMC8550959; doi:10.1038/s41380-019-0579-x)
Supplement: Supplementary file 14 — Supplementary Table S3A [file 41380_2019_579_MOESM14_ESM.docx]

Supplementary Table S3A. Comparison of physical, emotional, social, cognitive indicators of health of people in 3 personality networks in Young Finns Study

(n = 2126)

| Health Indicator | Creative  reliable network | Organized reliable network | Emotional unreliable network | ANOVA  F-stats | overall  p-value  (Tukey HSD Tests* ) |
| --- | --- | --- | --- | --- | --- |
| **Physical** |  |  |  |  |  |
| Cardio- vascular Health 2007 | 3.90 | 3.61 | 3.30 | 6.71 | <.0001  (1, 2) |
| Cardio-vascular Health 2012 | 4.10 | 3.93 | 3.66 | 4.1 | <0.03  (1, 3) |
|  |  |  |  |  |  |
| **Emotional** |  |  |  |  |  |
| Positive Affect 2012 | 4.02 | 3.96 | 3.45 | 95.92 | 0.0001  (1, 3) |
| Negative Affect 2012 | 2.21 | 2.02 | 2.53 | 10.93 | <.0001  (1, 2) |
|  |  |  |  |  |  |
| **Social** |  |  |  |  |  |
| Support 2001 | 4.44 | 4.32 | 3.78 | 2.84 | 0.05  (1, 3) |
| Support 2007 | 4.42 | 4.28 | 3.70 | 4.17 | 0.01  (1,3) |
|  |  |  |  |  |  |
| **Cognitive** |  |  |  |  |  |
| **(SD+CO)** |  |  |  |  |  |
| Normalized | 0.67 | 0.73 | 0.49 | 143.14 | <.0001  (2,3) |
| **(SDxCOxST)** |  |  |  |  |  |
| Normalized | 0.48 | 0.33 | 0.23 | 220.23 | <.0001  (2,3) |

*Tukey tests: 1 Creative

2 Organized

3 Emotional
