# Supplementary material for: Three genetic–environmental networks for human personality
Source: Mol Psychiatry. 2019 Nov 21;26(8):3858–75. doi: 10.1038/s41380-019-0579-x (PMC8550959; doi:10.1038/s41380-019-0579-x)
Supplement: Supplementary file 17 — Supplementary Table S5 [file 41380_2019_579_MOESM17_ESM.docx]

Supplementary Table S5. Comparison of the size of the 3 networks in terms of number of subjects and other components.

| **Characteristic** | **Network 1** | **Network 2** | **Network 3** |
| --- | --- | --- | --- |
| **Phenotypic** | Emotional-unreliable | Organized-reliable | Creative-reliable |
| # Subjects | 674 | 801 | 603 |
| # Temperament sets | 23 | 13 | 12 |
| # Character sets | 15 | 12 | 14 |
| **Genotypic** | Emotional  Reactivity | Intentional  Self-control | Creative  Self-awareness |
| # Subjects | 1486 | 1158 | 1141 |
| # SNP sets | 47 | 23 | 22 |
| # SNPs | 2490 | 2639 | 1704 |
| # genes mapped to SNPs | 251 | 442 | 578 |
