# Supplementary material for: Three genetic–environmental networks for human personality
Source: Mol Psychiatry. 2019 Nov 21;26(8):3858–75. doi: 10.1038/s41380-019-0579-x (PMC8550959; doi:10.1038/s41380-019-0579-x)
Supplement: Supplementary file 25 — Supplementary Table S13 [file 41380_2019_579_MOESM25_ESM.docx]

Supplementary Table S13: Estimation of variance explained (R^2^) by genotype alone (regression N_1_), environment alone (regression N_2_), and both jointly (regression N_3_) for 3 measures of health status (ill-being, well-being, and overall health)

| Status/R^2^ | Regression N_1_ | Regression N_2_ | Regression N_3_ |
| --- | --- | --- | --- |
| Ill-being | 0.58 | 0.27 | 0.68 |
| Well-being | 0.81 | 0.23 | 0.82 |
| Overall Health | 0.88 | 0.28 | 0.90 |
