# Supplementary material for: Three genetic–environmental networks for human personality
Source: Mol Psychiatry. 2019 Nov 21;26(8):3858–75. doi: 10.1038/s41380-019-0579-x (PMC8550959; doi:10.1038/s41380-019-0579-x)
Supplement: Supplementary file 26 — Supplementary Table S14 [file 41380_2019_579_MOESM26_ESM.docx]

**Supplementary Table S14:** Estimated coefficients for the model corresponding to regression N3 (full model), with number of observations: 1804; error degrees of freedom: 1787; Root Mean Squared Error: 0.253; R^2^-squared: 0.90; Adjusted R^2^ 0.896; F-statistic vs. constant model: 972; p-value < 1E-130 (red highlighted values indicate variables discarded by the Stepwise procedure, Matlab R2017b)

| **Regression Coefficient** | **Estimate** | **SE** | **t-statistic** | **p-value** |
| --- | --- | --- | --- | --- |
| intercept | 1.571 | 0.029 | 52.694 | <1e-130 |
| c1 | 0.850 | 0.164 | 5.183 | 2.43e-07 |
| c2 | -1.576 | 0.072 | -22.011 | 3.32e-95 |
| c3 | 0.087 | 0.157 | 0.556 | 0.58 |
| c4 | -1.738 | 0.098 | -17.647 | 2.26e-64 |
| c5 | 1.282 | 0.212 | 6.049 | 1.77e-09 |
| c6 | 1.668 | 0.122 | 13.632 | 2.50e-40 |
| c7 | -0.867 | 0.141 | - 6.147 | 9.42e-10 |
| c8 | 0.464 | 0.221 | 2.102 | 0.036 |
| c9 | 5.096 | 1.067 | 4.778 | 1.91e-06 |
| c10 | -6.216 | 1.264 | - 4.917 | 9.58e-07 |
| c11 | -0.922 | 0.243 | - 3.789 | 0.00016 |
| c12 | 1.112 | 0.315 | 3.533 | 0.00042 |
| c13 | 0.344 | 0.194 | 1.780 | 0.075 |
| c14 | -0.704 | 0.260 | - 2.707 | 0.0068 |
| c15 | 4.417 | 4.343 | 1.017 | 0.31 |
| c16 | -5.032 | 4.400 | - 1.149 | 0.25 |
